# Supplementary material for: The United Kingdom Research study into Ethnicity And COVID-19 outcomes in Healthcare workers (UK-REACH): protocol for a prospective longitudinal cohort study of healthcare and ancillary workers in UK healthcare settings
Source: BMJ Open. 2021 Sep 17;11(9):e050647. doi: 10.1136/bmjopen-2021-050647 (PMC8450967; doi:10.1136/bmjopen-2021-050647)
Supplement: Supplementary data [file bmjopen-2021-050647supp002.pdf]

Confidential

UK-REACH Questionnaire

Introduction

token ______________________

Thank you for taking part in the UK-REACH study on understanding Covid-19 outcomes for ethnic minority healthcare workers (https://uk-reach.org/).
The study is interested in healthcare workers from all backgrounds, and particularly in healthcare workers from ethnic minorities.
This questionnaire asks about your work and about your ethnic and cultural background. It also asks questions that will help the research team understand how work fits into your life more broadly, and how work affects your life. These include questions about:

• your age and other background information;
• your work in healthcare;
• your ethnicity, culture, religion, languages and education;
• your home environment and living circumstances;
• your health, both physical and mental;
• your attitudes and values in life and at work;
• your occupation and working life;
• your possible exposure to COVID-19;
• your experience of events before and during the UK national lockdown

Some questions may seem unusual and it will not always seem obvious why they are being asked. They are however important, and it would be helpful if you could answer as many as possible.

Some of the questions ask about sensitive topics, and if you cannot or do not wish to answer them then simply click on “Prefer not to answer”. Please be reassured that answers will be treated in accordance with strict research governance procedures, and the study has been reviewed by the Brighton and Sussex Research Ethics Committee.

Most people should be able to answer this questionnaire in about 30 minutes or so. Your answers will be stored as you go along, so you can pause the questionnaire and resume it later if you want. To do this, please select Save & Return Later. You can then continue the questionnaire from where you left off by selecting the Resume button in your UK REACH profile. You can return to your profile using the Return to Profile button provided.

If you need any further information about the study, or you have problems with any part of it, then the study team can be reached via email at uk-reach@leicester.ac.uk or by telephone on 07425611865.
If you are affected by any of the issues raised in this questionnaire or are looking for information on COVID-19 (coronavirus) please visit:
• Government guidelines: www.gov.uk/coronavirus
• NHS advice: https://www.nhs.uk/conditions/coronavirus-covid-19/symptoms/
• NHS – Where to get urgent help for mental health:
https://www.nhs.uk/using-the-nhs/nhs-services/mental-health-services/where-to-get-urgent-help-for-mental-health/
• Covid-19 Workforce Wellbeing: https://www.practitionerhealth.nhs.uk/covid-19-workforce-wellbeing
• Victim Support – support for people affected by crime or traumatic events, including hate crime:
https://www.victimsupport.org.uk/
• Samaritans – Emotional support for everyone: www.samaritans.org
• Mind – Advice and support for anyone with a mental health problem: www.mind.org.uk

UK-REACH ID
This has been autocompleted. This ID is used for research purposes only. ______________________

Your UK-REACH ID is Empty!
Please return to your user profile and use the link provided there to access the questionnaire.

Return to User Profile

Introduction

If you wish to go back to an earlier question, please use the Previous Page button at the bottom of each page. Please do not use the back button on your browser as that will mean that you leave the questionnaire.

Section 1/10: Background information

0% complete
.

These questions ask for some simple information about you.

There will be a separate section on your ethnic and cultural background later in the questionnaire.

Confidential

Section 1/10: Background information

0% complete
.

Which of the following best describes you?	◯ Man<br>◯ Woman<br>◯ I use another term to describe my gender<br>◯ Prefer not to answer
Please enter the term you use to describe your gender, or enter "Prefer not to answer".	________________________________
What was your sex assigned at birth?	◯ Male<br>◯ Female<br>◯ Other<br>◯ Prefer not to answer
Which of the following best describes your marital status?<br><br>You will be asked more about who you live with later in the questionnaire.	◯ Single (never married or in civil partnership)<br>◯ Cohabiting with a partner<br>◯ Married (including those in civil partnerships)<br>◯ Divorced (including formerly in a civil partnership which is now legally dissolved)<br>◯ Separated (but still legally married or in a civil partnership)<br>◯ Widowed (including surviving partner from a civil partnership)<br>◯ Prefer not to answer

Confidential

Section 2/10: Your Job

1% complete
.

There are many different types of healthcare jobs, and the study needs to know some things about your job.

Some people have more than one job or several roles within their job. These questions are principally about your main role/job. This is usually the role/job you spend most time in per week. If you have roles/jobs in both clinical and non-clinical settings, please think about the main role/job you have in a clinical setting, even if it is not the job you spend most time in each week.

You will also be asked about personal protective equipment (PPE).

Woolf K, *et al. BMJ Open* 2021; 11:e050647. doi: 10.1136/bmjopen-2021-050647

Section 2/10: Your job

1% complete

.

Woolf K, *et al. BMJ Open* 2021; 11:e050647. doi: 10.1136/bmjopen-2021-050647

Confidential

What is your main job/role? Please choose the best fit and specify further if you wish.

If you are not currently working, please answer about your most recent role.

- ◯ Allied Health Professional - Arts therapist
- ◯ Allied Health Professional - Biomedical scientist
- ◯ Allied Health Professional - Chiropodist/Podiatrist
- ◯ Allied Health Professional - Clinical scientist
- ◯ Allied Health Professional - Dietician
- ◯ Allied Health Professional - Hearing aid dispenser
- ◯ Allied Health Professional - Occupational therapist
- ◯ Allied Health Professional - Operating department practicioner
- ◯ Allied Health Professional - Orthoptist
- ◯ Allied Health Professional - Physiotherapist
- ◯ Allied Health Professional - Practitioner psychologist
- ◯ Allied Health Professional - Prosthetist / Orthotist
- ◯ Allied Health Professional - Radiographer
- ◯ Allied Health Professional - Speech and language therapist
- ◯ Allied Health Professional - Other Allied Health Professional role (please specify)
- ◯ Ambulance - Emergency medical technician
- ◯ Ambulance - Paramedic
- ◯ Ambulance - Other ambulance role (please specify)
- ◯ Clinical support staff - OT Support Worker
- ◯ Clinical support staff - Phlebotomist
- ◯ Clinical support staff - Physiotherapy Assistant
- ◯ Clinical support staff - Radiography Assistant
- ◯ Clinical support staff - Other clinical support role (please specify)
- ◯ Dental - Clinical dental technician
- ◯ Dental - Dental Hygienist
- ◯ Dental - Dental nurse
- ◯ Dental - Dental technician
- ◯ Dental - Dentist
- ◯ Dental - Other dental role (please specify)
- ◯ Doctors - Doctor
- ◯ Medical associates - Advanced Critical Care Practitioner
- ◯ Medical associates - Anaesthesia associate
- ◯ Medical associates - Physician Associate
- ◯ Medical associates - Surgical Care Practitioner
- ◯ Medical associates - Other medical associate (please specify)
- ◯ Nursing and midwifery - Advanced Nurse Practitioner
- ◯ Nursing and midwifery - Healthcare assistant
- ◯ Nursing and midwifery - Maternity support worker
- ◯ Nursing and midwifery - Midwife
- ◯ Nursing and midwifery - Nurse
- ◯ Nursing and midwifery - Nursing Associate
- ◯ Nursing and midwifery - Other nursing and midwifery role (please specify)
- ◯ Pharmacy - Pharmacist
- ◯ Pharmacy - Pharmacy technician
- ◯ Pharmacy - Other pharmacy role (please specify)
- ◯ Optical - Dispensing optician
- ◯ Optical - Optometrist
- ◯ Other Optical role (please specify)
- ◯ Wider healthcare role - Administration
- ◯ Wider healthcare role - Catering services
- ◯ Wider healthcare role - Domestic services
- ◯ Wider healthcare role - Estates services
- ◯ Wider healthcare role - Porter
- ◯ Wider healthcare role - Other (Please specify)
- ◯ Any other role (please specify)
- ◯ Prefer not to answer

Confidential

Please specify your wider healthcare role: __________________________________

Please specify your Allied Health Professional role: __________________________________

Please specify your ambulance role: __________________________________

Please specify your clinical support staff role: __________________________________

Please specify your dental role: __________________________________

Please specify your medical associates role: __________________________________

Please specify your nursing and midwifery role: __________________________________

Please specify your pharmacy role: __________________________________

Please specify your optical role: __________________________________

Please specify your job role: __________________________________

Please tell us about your working status now, and in the first month after the start of the UK national lockdown on 23rd March 2020:

	Not working	Working	Prefer not to answer
Now	○	○	○
In the first month after the start of the UK national lockdown on 23rd March 2020	○	○	○

Please indicate the reason(s) you are not working now (Select all that apply):

By 'shielding' we mean taking extra steps to protect yourself, by minimising interactions between yourself and others because you are at high risk of severe illness from coronavirus (COVID-19).

By 'On furlough', we mean paid by your employer through the Job Support Scheme or Job Retention Scheme and not allowed to work.

- ☐ Shielding due to own health
- ☐ Shielding due to household member's health
- ☐ On furlough
- ☐ On sick leave
- ☐ On carer's leave
- ☐ Unemployed
- ☐ Retired
- ☐ Other (please specify)
- ☐ Prefer not to answer

Please specify why you are not currently working: __________________________________

Woolf K, *et al. BMJ Open* 2021; 11:e050647. doi: 10.1136/bmjopen-2021-050647

Please indicate the reason(s) you were not working at the start of the UK national lockdown on 23rd March 2020 (select all that apply):<br><br>By 'shielding' we mean taking extra steps to protect yourself, by minimising interactions between yourself and others because you are at high risk of severe illness from coronavirus (COVID-19).<br><br>By 'On furlough', we mean paid by your employer through the Job Support Scheme or Job Retention Scheme and not allowed to work.	☐ Shielding due to own health<br>☐ Shielding due to household member's health<br>☐ On furlough<br>☐ On sick leave<br>☐ On carer's leave<br>☐ Unemployed<br>☐ Retired<br>☐ Other (please specify)<br>☐ Prefer not to answer
Please specify why you were not working at the start of the UK national lockdown on 23rd March 2020:	__________________________
In which of the following sectors is your current main job/ role?<br><br>If not currently working, please answer for your most recent main job/role.<br><br>Select all that apply.	☐ NHS<br>☐ Other public sector (e.g. local or national government)<br>☐ Private sector<br>☐ Private facility temporarily used by the NHS<br>☐ University / higher education<br>☐ Prefer not to answer
It would be helpful to us to know where in the UK your main job/role is located. Please type the first part of the postcode (e.g. W1G, CF24, BT12 or EH16). If you can't remember the number at the end, just type the first letters (e.g. CF, BT or EH).<br><br>If not currently working, please answer for your most recent main job/role.<br><br>Please note: We cannot directly identify your place of work from the first part of the postcode, but it does give a good indication of geographical location, such that place of work may be inferred.<br><br>You may enter "Prefer not to answer" if you do not wish to provide this information.	__________________________
In which of the following sectors was your main job/role in the first month after the start of the UK national lockdown on 23rd March 2020?<br><br>Select all that apply.	☐ NHS<br>☐ Other public sector (e.g. local or national government)<br>☐ Private sector<br>☐ Private facility temporarily used by the NHS<br>☐ University / higher education<br>☐ Prefer not to answer

Confidential

What is your current or most recent grade?	◯ Doctor in training post - Foundation level<br>◯ Doctor in training post - Core level<br>◯ Doctor in training post - Specialty level<br>◯ Locally employed / trust doctor - Foundation level<br>◯ Locally employed / trust doctor - Core level<br>◯ Locally employed / trust doctor - Specialty level<br>◯ GP<br>◯ Consultant<br>◯ SAS<br>◯ Other (please specify)<br>◯ Prefer not to answer
Please specify your current or most recent grade:	__________________________________
What was your grade at the start of the UK national lockdown on 23rd March 2020?	◯ Doctor in training post - Foundation level<br>◯ Doctor in training post - Core level<br>◯ Doctor in training post - Specialty level<br>◯ Locally employed / trust doctor - Foundation level<br>◯ Locally employed / trust doctor - Core level<br>◯ Locally employed / trust doctor - Specialty level<br>◯ GP<br>◯ Consultant<br>◯ SAS<br>◯ Other (please specify)<br>◯ Prefer not to answer
Please specify your grade:	__________________________________

Woolf K, *et al. BMJ Open* 2021; 11:e050647. doi: 10.1136/bmjopen-2021-050647

Confidential

What is your current or most recent specialty?

- ◯ Acute internal medicine
- ◯ Allergy
- ◯ Anaesthetics
- ◯ Audio vestibular medicine
- ◯ Aviation and space medicine
- ◯ Cardio-thoracic surgery
- ◯ Cardiology
- ◯ Chemical pathology
- ◯ Child and adolescent psychiatry
- ◯ Child mental health
- ◯ Clinical genetics
- ◯ Clinical neurophysiology
- ◯ Clinical oncology
- ◯ Clinical pharmacology and therapeutics
- ◯ Clinical radiology
- ◯ Community child health
- ◯ Community sexual and reproductive health
- ◯ Congenital cardiac surgery
- ◯ Cytopathology
- ◯ Dermatology
- ◯ Diagnostic neuropathology
- ◯ Emergency medicine
- ◯ Endocrinology and diabetes mellitus
- ◯ Forensic histopathology
- ◯ Forensic psychiatry
- ◯ Gastroenterology
- ◯ General (internal) medicine
- ◯ General practice
- ◯ General psychiatry
- ◯ General surgery
- ◯ Genitourinary medicine
- ◯ Geriatric medicine
- ◯ Gynaecological oncology
- ◯ Haematology
- ◯ Hepatology
- ◯ Histopathology
- ◯ Immunology
- ◯ Infectious diseases
- ◯ Intensive care medicine
- ◯ Interventional radiology
- ◯ Liaison psychiatry
- ◯ Maternal and fetal medicine
- ◯ Medical microbiology
- ◯ Medical oncology
- ◯ Medical ophthalmology
- ◯ Medical psychotherapy
- ◯ Medical virology
- ◯ Metabolic medicine
- ◯ Neonatal medicine
- ◯ Neurology
- ◯ Neurosurgery
- ◯ Nuclear medicine
- ◯ Obstetrics and gynaecology
- ◯ Occupational medicine
- ◯ Old age psychiatry
- ◯ Ophthalmology
- ◯ Oral and maxillofacial surgery
- ◯ Otolaryngology
- ◯ Paediatric allergy, immunology and infectious diseases
- ◯ Paediatric clinical pharmacology and therapeutics
- ◯ Paediatric diabetes and endocrinology
- ◯ Paediatric emergency medicine
- ◯ Paediatric gastroenterology, hepatology and nutrition
- ◯ Paediatric inherited metabolic medicine
- ◯ Paediatric intensive care medicine
- ◯ Paediatric nephrology
- ◯ Paediatric neurodisability

◯ Paediatric neurology
◯ Paediatric oncology
◯ Paediatric palliative medicine
◯ Paediatric respiratory medicine
◯ Paediatric rheumatology
◯ Paediatric and perinatal pathology
◯ Paediatric cardiology
◯ Paediatric surgery
◯ Paediatrics
◯ Palliative medicine
◯ Pharmaceutical medicine
◯ Plastic surgery
◯ Pre-hospital emergency medicine
◯ Psychiatry of learning disability
◯ Public health medicine
◯ Rehabilitation medicine
◯ Rehabilitation psychiatry
◯ Renal medicine
◯ Reproductive medicine
◯ Respiratory medicine
◯ Rheumatology
◯ Sport and exercise medicine
◯ Stroke medicine
◯ Substance misuse psychiatry
◯ Trauma and orthopaedic surgery
◯ Tropical medicine
◯ Urogynaecology
◯ Urology
◯ Vascular surgery
◯ Prefer not to answer

What was your specialty in the first month after the start of the UK national lockdown on 23rd March 2020?	◯ Acute internal medicine<br>◯ Allergy<br>◯ Anaesthetics<br>◯ Audio vestibular medicine<br>◯ Aviation and space medicine<br>◯ Cardio-thoracic surgery<br>◯ Cardiology<br>◯ Chemical pathology<br>◯ Child and adolescent psychiatry<br>◯ Child mental health<br>◯ Clinical genetics<br>◯ Clinical neurophysiology<br>◯ Clinical oncology<br>◯ Clinical pharmacology and therapeutics<br>◯ Clinical radiology<br>◯ Community child health<br>◯ Community sexual and reproductive health<br>◯ Congenital cardiac surgery<br>◯ Cytopathology<br>◯ Dermatology<br>◯ Diagnostic neuropathology<br>◯ Emergency medicine<br>◯ Endocrinology and diabetes mellitus<br>◯ Forensic histopathology<br>◯ Forensic psychiatry<br>◯ Gastroenterology<br>◯ General (internal) medicine<br>◯ General practice<br>◯ General psychiatry<br>◯ General surgery<br>◯ Genitourinary medicine<br>◯ Geriatric medicine<br>◯ Gynaecological oncology<br>◯ Haematology<br>◯ Hepatology<br>◯ Histopathology<br>◯ Immunology<br>◯ Infectious diseases<br>◯ Intensive care medicine<br>◯ Interventional radiology<br>◯ Liaison psychiatry<br>◯ Maternal and fetal medicine<br>◯ Medical microbiology<br>◯ Medical oncology<br>◯ Medical ophthalmology<br>◯ Medical psychotherapy<br>◯ Medical virology<br>◯ Metabolic medicine<br>◯ Neonatal medicine<br>◯ Neurology<br>◯ Neurosurgery<br>◯ Nuclear medicine<br>◯ Obstetrics and gynaecology<br>◯ Occupational medicine<br>◯ Old age psychiatry<br>◯ Ophthalmology<br>◯ Oral and maxillofacial surgery<br>◯ Otolaryngology<br>◯ Paediatric allergy, immunology and infectious diseases<br>◯ Paediatric clinical pharmacology and therapeutics<br>◯ Paediatric diabetes and endocrinology<br>◯ Paediatric emergency medicine<br>◯ Paediatric gastroenterology, hepatology and nutrition<br>◯ Paediatric inherited metabolic medicine<br>◯ Paediatric intensive care medicine<br>◯ Paediatric nephrology<br>◯ Paediatric neurodisability

	◯ Paediatric neurology<br>◯ Paediatric oncology<br>◯ Paediatric palliative medicine<br>◯ Paediatric respiratory medicine<br>◯ Paediatric rheumatology<br>◯ Paediatric and perinatal pathology<br>◯ Paediatric cardiology<br>◯ Paediatric surgery<br>◯ Paediatrics<br>◯ Palliative medicine<br>◯ Pharmaceutical medicine<br>◯ Plastic surgery<br>◯ Pre-hospital emergency medicine<br>◯ Psychiatry of learning disability<br>◯ Public health medicine<br>◯ Rehabilitation medicine<br>◯ Rehabilitation psychiatry<br>◯ Renal medicine<br>◯ Reproductive medicine<br>◯ Respiratory medicine<br>◯ Rheumatology<br>◯ Sport and exercise medicine<br>◯ Stroke medicine<br>◯ Substance misuse psychiatry<br>◯ Trauma and orthopaedic surgery<br>◯ Tropical medicine<br>◯ Urogynaecology<br>◯ Urology<br>◯ Vascular surgery<br>◯ Prefer not to answer
What is your current or most recent NHS band?	◯ Band 1<br>◯ Band 2<br>◯ Band 3<br>◯ Band 4<br>◯ Band 5<br>◯ Band 6<br>◯ Band 7<br>◯ Band 8a<br>◯ Band 8b<br>◯ Band 8c<br>◯ Band 8d<br>◯ Band 9<br>◯ Prefer not to answer
What was your NHS band at the start of the UK national lockdown on 23rd March 2020?	◯ Band 1<br>◯ Band 2<br>◯ Band 3<br>◯ Band 4<br>◯ Band 5<br>◯ Band 6<br>◯ Band 7<br>◯ Band 8a<br>◯ Band 8b<br>◯ Band 8c<br>◯ Band 8d<br>◯ Band 9<br>◯ Prefer not to answer

Confidential

What is your registered field of nursing?	◯ Adult<br>◯ Children's<br>◯ Learning disability<br>◯ Mental health<br>◯ Dual registration (please specify)<br>◯ Prefer not to answer
Please specify the two fields in which you practice:	__________________________________

Confidential

Section 2/10: Your job

7% complete
.

Please indicate which areas you work in a typical week now, and which areas you worked in a typical week during the first month after the UK national lockdown on 23rd March 2020.

If you work in the same area(s) now as you were working in the first month after the UK national lockdown, please tick the “now” and “UK national lockdown” boxes.

Please leave blank any areas you have not worked in since the UK national lockdown.

	Now	UK national lockdown	Prefer not to answer
Ambulance (inc air ambulance)	☐	☐	☐
Armed forces	☐	☐	☐
Community clinical / primary care setting	☐	☐	☐
Community non-clinical settings	☐	☐	☐
Hospitals - Emergency Department	☐	☐	☐
Hospital - Intensive Care Unit	☐	☐	☐
Hospital - other inpatient setting	☐	☐	☐
Hospital - outpatients	☐	☐	☐
Hospital - other clinical setting	☐	☐	☐
Hospital - other non-clinical setting	☐	☐	☐
Hospital - public / communal areas	☐	☐	☐
Laboratory	☐	☐	☐
Maternity	☐	☐	☐
Mobile across areas	☐	☐	☐
Nursing or care home	☐	☐	☐
Prison	☐	☐	☐
Psychiatric hospital or inpatient unit	☐	☐	☐
University	☐	☐	☐
Your home	☐	☐	☐
Other (Please specify)	☐	☐	☐

Please specify the workplace for which you selected "Other": ________________________________

At present, how many hours do you work in a typical week? ________________________________

Woolf K, *et al. BMJ Open* 2021; 11:e050647. doi: 10.1136/bmjopen-2021-050647

In the first month after the start of the UK national lockdown on 23rd March 2020, how many hours did you work in a typical week? ________________________________

How often do you work night shifts now, and did you typically work in the early months after the start of the UK national lockdown on 23rd March 2020?

If these are on call shifts, please answer based on how often you are actually required to work.
If you were not working during the specified time frame please select "Not applicable".
If you are answering this questionnaire on a smartphone, you may find it easier to view by rotating the screen.

	Not applicable	Never	Less than once a month	Once a month or more, but not every week	Once a week or more, but not every shift	I always work nights	Prefer not to answer
Now	○	○	○	○	○	○	○
In the early months following the start of the UK national lockdown on 23rd March 2020	○	○	○	○	○	○	○

Confidential

Section 2/10: Your job

8% complete
.

How many people did you talk with at work last week from each of the following groups:

If you are answering this questionnaire on a smartphone, you may find it easier to view by rotating the screen.

Remotely (e.g. over the phone or via video media)

	0	1-5	6-20	21-50	51+	Prefer not to answer
Number of patients	○	○	○	○	○	○
Number of colleagues	○	○	○	○	○	○
Number of others (not patients or colleagues)	○	○	○	○	○	○

Face to face with social distancing

	0	1-5	6-20	21-50	51+	Prefer not to answer
Number of patients with confirmed or suspected COVID-19	○	○	○	○	○	○
Number of other patients	○	○	○	○	○	○
Number of colleagues	○	○	○	○	○	○
Number of others (not patients or colleagues)	○	○	○	○	○	○

With physical contact

	0	1-5	6-20	21-50	51+	Prefer not to answer
Number of patients with confirmed or suspected COVID-19	○	○	○	○	○	○
Number of other patients	○	○	○	○	○	○
Number of colleagues	○	○	○	○	○	○
Number of others (not patients or colleagues)	○	○	○	○	○	○

Woolf K, *et al. BMJ Open* 2021; 11:e050647. doi: 10.1136/bmjopen-2021-050647

Setion 2/10: Your job

8% complete
.

How many people did you talk with at work in a typical week during the first month after the start of the UK national lockdown on 23rd March 2020 from each of the following groups:
If you are answering this questionnaire on a smartphone, you may find it easier to view by rotating the screen.

Remotely (e.g. over the phone, or via video media)

	0	1-5	6-20	21-50	51+	Prefer not to answer
Number of patients	○	○	○	○	○	○
Number of colleagues	○	○	○	○	○	○
Number of others (not patients or colleagues)	○	○	○	○	○	○

Face to face with social distancing

	0	1-5	6-20	21-50	51+	Prefer not to answer
Number of patients with confirmed or suspected COVID-19	○	○	○	○	○	○
Number of other patients	○	○	○	○	○	○
Number of colleagues	○	○	○	○	○	○
Number of others (not patients or colleagues)	○	○	○	○	○	○

With physical contact

	0	1-5	6-20	21-50	51+	Prefer not to answer
Number of patients with confirmed or suspected COVID-19	○	○	○	○	○	○
Number of other patients	○	○	○	○	○	○
Number of colleagues	○	○	○	○	○	○
Number of others (not patients or colleagues)	○	○	○	○	○	○

Confidential

Section 2/10: Your job

9% complete
.

When working in your main role/job, how long do you spend travelling to and from work per day?

You may select 'Not at all' if you work exclusively from home.

If you are answering this questionnaire on a smartphone, you may find it easier to view by rotating the screen.

	Not at all	Less than 10 minutes	10 minutes to 1 hour	1 to 2 hours	Over 2 hours	Prefer not to answer
Typical working day over the past month	○	○	○	○	○	○
Typical working day during the first month after the UK national lockdown on 23rd March 2020	○	○	○	○	○	○

Which of the following modes of transport do you use to commute on a typical working day over the past month?
Please select all that apply.

- ☐ Car, alone or with member of household
- ☐ Car share, with a small pool of people outside of household
- ☐ Taxi or private hire vehicle
- ☐ Public transport (e.g. bus, train, tram, underground)
- ☐ Motorcycle, scooter or moped
- ☐ Bicycle
- ☐ On foot
- ☐ Other

Please enter the mode of transport for which you selected 'Other': __________________________________

Which of the following modes of transport did you use to commute on a typical working day, during the first month after the start of the UK national lockdown on 23 March 2020?
Please select all that apply.

- ☐ Car, alone or with member of household
- ☐ Car share, with a small pool of people outside of household
- ☐ Taxi or private hire vehicle
- ☐ Public transport (e.g. bus, train, tram, underground)
- ☐ Motorcycle, scooter or moped
- ☐ Bicycle
- ☐ On foot
- ☐ Other

Please enter the mode of transport for which you selected 'Other': __________________________________

Section 2/10: Your job

11% complete
.

This question is about your access to appropriate personal protective equipment (PPE) at work.

If you have more than one role or job, please consider your access to personal protective equipment (PPE) in general, across all of your jobs.

	Not applicable	Not at all	Rarely	Some of the time	Yes, most of the time	Yes, all of the time	Prefer not to answer
At present, do you have access to appropriate personal protective equipment (PPE) at work?	○	○	○	○	○	○	○
In the first month after the start of the UK national lockdown on 23rd March 2020, did you have access to appropriate personal protective equipment (PPE) at work?	○	○	○	○	○	○	○

This question is about any training you may have received in the use of personal protective equipment (PPE) in your main job/role.

Select all that apply.

	Not applicable	Formal training in person	Formal training online	Informal training	No training	Prefer not to answer
Have you received training in the use of personal protective equipment (PPE) for your current work?	☐	☐	☐	☐	☐	☐
In the first month after the start of the UK national lockdown on 23rd March 2020, did you receive training in the use of personal protective equipment (PPE) for your work?	☐	☐	☐	☐	☐	☐

This question is about your exposure to aerosol-generating procedures at work.

Aerosol generating procedures include intubation, extubation, bronchoscopy, non-invasive ventilation, respiratory suctioning, and dental procedures using high speed drills.

	Not applicable	Never	Once a month or less	A few times a month	Once a week	A few times a week	Every day	Prefer not to answer
At present, how often are you in a room where aerosol-generating procedures are performed?	○	○	○	○	○	○	○	○
In the first month after the start of the UK national lockdown on 23rd March 2020, how often were you in a room where aerosol-generating procedures are performed?	○	○	○	○	○	○	○	○

Section 2/10: Your job

13% complete
.

Have you been offered an NHS COVID-19 risk assessment at work?

- ◯ Not applicable
- ◯ No, I have not heard of COVID-19 risk assessments
- ◯ No, I have not been offered a COVID-19 risk assessment
- ◯ Yes, I have been offered a COVID-19 risk assessment but I chose not to complete it
- ◯ Yes, I have been offered a COVID-19 risk assessment and I completed it
- ◯ Prefer not to answer

Did your work change as a result of the NHS COVID-19 risk assessment result? (Select the best answer)

- ◯ No, because it did not need to
- ◯ No, because I did not want it to
- ◯ No, but I did want it to
- ◯ Yes, different duties (e.g. advised not to conduct aerosol-generating procedures)
- ◯ Yes, reduced patient contact
- ◯ Yes, removed patient contact
- ◯ Yes, advised to shield
- ◯ Yes, other (please specify)
- ◯ Prefer not to answer

Please specify how your work changed as a result of the NHS COVID-19 risk assessment result: __________________________________

Thinking about where you work in your current main job/role, to what extent do you agree with the following statements?
If you are answering this questionnaire on a smartphone, you may find it easier to view by rotating the screen.

	Strongly disagree	Disagree	Neither agree or disagree	Agree	Strongly agree	Not applicable	Prefer not to answer
I would feel secure raising concerns about unsafe clinical practice	◯	◯	◯	◯	◯	◯	◯
I am confident that my organisation would address my concern	◯	◯	◯	◯	◯	◯	◯

Section 2/10: Your job

14% complete
.

During the UK national lockdown that began on 23rd March 2020, were you redeployed to a different role because of the pandemic?	◯ No<br>◯ Yes, I was redeployed but continued to work in my role prior to the UK national lockdown more than half the time<br>◯ Yes, I was redeployed but continued to work in my role prior to the UK national lockdown for less than half the time<br>◯ Yes, I was redeployed all of the time<br>◯ Prefer not to answer
Compared to your role before the start of UK national lockdown on 23 March 2020, how much direct patient contact is there or was there in your redeployed role?	◯ Much less<br>◯ Somewhat less<br>◯ About the same<br>◯ Somewhat more<br>◯ Much more<br>◯ Prefer not to answer
Did you have any of the following in your redeployment?<br><br>Select all that apply	☐ Formal training face to face<br>☐ Formal training online<br>☐ Informal training<br>☐ No training<br>☐ Prefer not to answer
Did you have any of the following types of supervision in your redeployment?<br>Select all that apply	☐ Formal supervision<br>☐ Informal supervision<br>☐ No supervision<br>☐ Prefer not to answer

Section 3/10: Your ethnic, cultural and national identity and background

17% complete
.

Ethnicity relates to a range of different factors. Most studies of ethnicity in healthcare workers use the standard UK Census question on ethnicity with its broad but limited ethnic groupings.

We will start with the UK Census ethnicity question but we recognise its limitations in describing the subtleties of how people vary in relation to migration, social identity, and experiences of them and their families. We hope this more detailed information will help in understanding how COVID-19 affects different groups.

This section asks about:

• Ethnic group and place of birth of you and your parents
• English and other languages spoken
• The role of religion in your life
• Your sense of identity
• The ethnicity of those you work with

Please remember that if you prefer not to answer any question then simply click on Prefer not to answer, but remember that there are good reasons for asking these questions

BMJ Publishing Group Limited (BMJ) disclaims all liability and responsibility arising from any reliance placed on this supplemental material which has been supplied by the author(s)

Confidential

Section 3/10: Your ethnic, cultural and national identity and background

17% complete
.

What is your ethnic group?

Select the one that best describes your ethnic group or background.
The categories are the ethnic groups used in the UK National Census.

- ◯ Asian/Asian British - Indian
- ◯ Asian/Asian British - Pakistani
- ◯ Asian/Asian British - Bangladeshi
- ◯ Asian/Asian British - Chinese
- ◯ Asian/Asian British - Any other Asian background
- ◯ Black/African/Caribbean/Black British - African
- ◯ Black/African/Caribbean/Black British - Caribbean
- ◯ Black/African/Caribbean/Black British - Any other Black/African/Caribbean background
- ◯ Mixed/Multiple ethnic groups - White and Black Caribbean
- ◯ Mixed/Multiple ethnic groups - White and Black African
- ◯ Mixed/Multiple ethnic groups - White and Asian
- ◯ Mixed/Multiple ethnic groups - Any other Mixed/multiple ethnic background
- ◯ White - English/Welsh/Scottish/Northern Irish/British
- ◯ White - Irish
- ◯ White - Gypsy or Irish Traveller
- ◯ White - Any other white background
- ◯ Other ethnic group - Arab
- ◯ Other ethnic group - Any other ethnic background
- ◯ Prefer not to say

Please specify your ethnic group:

____

Were you born in the UK?

- ◯ No
- ◯ Yes
- ◯ Prefer not to answer

Woolf K, *et al. BMJ Open* 2021; 11:e050647. doi: 10.1136/bmjopen-2021-050647

In which country were you born?

◯ Afghanistan
◯ Albania
◯ Algeria
◯ Andorra
◯ Angola
◯ Antigua and Barbuda
◯ Argentina
◯ Armenia
◯ Australia
◯ Austria
◯ Azerbaijan
◯ Bahamas, The
◯ Bahrain
◯ Bangladesh
◯ Barbados
◯ Belarus
◯ Belgium
◯ Belize
◯ Benin
◯ Bhutan
◯ Bolivia
◯ Bosnia and Herzegovina
◯ Botswana
◯ Brazil
◯ Brunei
◯ Bulgaria
◯ Burkina Faso
◯ Burundi
◯ Cambodia
◯ Cameroon
◯ Canada
◯ Cape Verde
◯ Central African Republic
◯ Chad
◯ Chile
◯ China
◯ Colombia
◯ Comoros
◯ Congo
◯ Congo (Democratic Republic)
◯ Costa Rica
◯ Croatia
◯ Cuba
◯ Cyprus
◯ Czechia
◯ Denmark
◯ Djibouti
◯ Dominica
◯ Dominican Republic
◯ East Timor
◯ Ecuador
◯ Egypt
◯ El Salvador
◯ Equatorial Guinea
◯ Eritrea
◯ Estonia
◯ Eswatini
◯ Ethiopia
◯ Fiji
◯ Finland
◯ France
◯ Gabon
◯ Gambia, The
◯ Georgia
◯ Germany
◯ Ghana
◯ Greece
◯ Grenada
◯ Guatemala

◯ Guinea Republic of Guinea
◯ Guinea-Bissau
◯ Guyana
◯ Haiti
◯ Honduras
◯ Hungary
◯ Iceland
◯ India
◯ Indonesia
◯ Iran
◯ Iraq
◯ Ireland
◯ Israel
◯ Italy
◯ Ivory Coast
◯ Jamaica
◯ Japan
◯ Jordan
◯ Kazakhstan
◯ Kenya
◯ Kiribati
◯ Korea (North)
◯ Korea (South)
◯ Kosovo
◯ Kuwait
◯ Kyrgyzstan
◯ Laos
◯ Latvia
◯ Lebanon
◯ Lesotho
◯ Liberia
◯ Libya
◯ Liechtenstein
◯ Lithuania
◯ Luxembourg
◯ Madagascar
◯ Malawi
◯ Malaysia
◯ Maldives
◯ Mali
◯ Malta
◯ Marshall Islands
◯ Mauritania
◯ Mauritius
◯ Mexico
◯ Micronesia
◯ Moldova
◯ Monaco
◯ Mongolia
◯ Montenegro
◯ Morocco
◯ Mozambique
◯ Myanmar (Burma)
◯ Namibia
◯ Nauru
◯ Nepal
◯ Netherlands
◯ New Zealand
◯ Nicaragua
◯ Niger
◯ Nigeria
◯ North Macedonia
◯ Norway
◯ Oman
◯ Pakistan
◯ Palau
◯ Panama
◯ Papua New Guinea
◯ Paraguay
◯ Peru
◯ Philippines

◯ Poland
◯ Portugal
◯ Qatar
◯ Romania
◯ Russia
◯ Rwanda
◯ St Kitts and Nevis
◯ St Lucia
◯ St Vincent
◯ Samoa
◯ San Marino
◯ Sao Tome and Principe
◯ Saudi Arabia
◯ Senegal
◯ Serbia
◯ Seychelles
◯ Sierra Leone
◯ Singapore
◯ Slovakia
◯ Slovenia
◯ Solomon Islands
◯ Somalia
◯ South Africa
◯ South Sudan
◯ Spain
◯ Sri Lanka
◯ Sudan
◯ Suriname
◯ Sweden
◯ Switzerland
◯ Syria
◯ Taiwan
◯ Tajikistan
◯ Tanzania
◯ Thailand
◯ Togo
◯ Tonga
◯ Trinidad and Tobago
◯ Tunisia
◯ Turkey
◯ Turkmenistan
◯ Tuvalu
◯ Uganda
◯ Ukraine
◯ United Arab Emirates
◯ United States
◯ Uruguay
◯ Uzbekistan
◯ Vanuatu
◯ Vatican City
◯ Venezuela
◯ Vietnam
◯ Yemen
◯ Zambia
◯ Zimbabwe
◯ Other (Please specify)
◯ Prefer not to answer

Please specify the country in which you were born: __________________________

In which year did you move to the UK?

If you are unsure, please give your best estimate. __________________________ ((Please enter in format YYYY, e.g. 1967))

For which countries do you hold a passport?

We have provided boxes to select up to three nationalities. Please leave the boxes for nationality 2 and nationality 3 blank if they do not apply to you.

Woolf K, *et al. BMJ Open* 2021; 11:e050647. doi: 10.1136/bmjopen-2021-050647

Confidential

Nationality 1:

◯ United Kingdom ◯ Afghanistan
◯ Albania ◯ Algeria ◯ Andorra
◯ Angola ◯ Antigua and Barbuda
◯ Argentina ◯ Armenia
◯ Australia ◯ Austria
◯ Azerbaijan ◯ Bahamas, The
◯ Bahrain ◯ Bangladesh
◯ Barbados ◯ Belarus
◯ Belgium ◯ Belize ◯ Benin
◯ Bhutan ◯ Bolivia ◯ Bosnia and Herzegovina ◯ Botswana
◯ Brazil ◯ Brunei ◯ Bulgaria
◯ Burkina Faso ◯ Burundi
◯ Cambodia ◯ Cameroon
◯ Canada ◯ Cape Verde
◯ Central African Republic
◯ Chad ◯ Chile ◯ China
◯ Colombia ◯ Comoros
◯ Congo ◯ Congo (Democratic Republic)
◯ Costa Rica ◯ Croatia
◯ Cuba ◯ Cyprus ◯ Czechia
◯ Denmark ◯ Djibouti
◯ Dominica ◯ Dominican Republic
◯ East Timor ◯ Ecuador
◯ Egypt ◯ El Salvador
◯ Equatorial Guinea ◯ Eritrea
◯ Estonia ◯ Eswatini
◯ Ethiopia ◯ Fiji ◯ Finland
◯ France ◯ Gabon ◯ Gambia, The
◯ Georgia ◯ Germany
◯ Ghana ◯ Greece ◯ Grenada
◯ Guatemala ◯ Guinea Republic of Guinea
◯ Guinea-Bissau ◯ Guyana
◯ Haiti ◯ Honduras ◯ Hungary
◯ Iceland ◯ India ◯ Indonesia
◯ Iran ◯ Iraq ◯ Ireland
◯ Israel ◯ Italy ◯ Ivory Coast
◯ Jamaica ◯ Japan ◯ Jordan
◯ Kazakhstan ◯ Kenya
◯ Kiribati ◯ Korea (North)
◯ Korea (South) ◯ Kosovo
◯ Kuwait ◯ Kyrgyzstan
◯ Laos ◯ Latvia ◯ Lebanon
◯ Lesotho ◯ Liberia
◯ Libya ◯ Liechtenstein
◯ Lithuania ◯ Luxembourg
◯ Madagascar ◯ Malawi
◯ Malaysia ◯ Maldives
◯ Mali ◯ Malta ◯ Marshall Islands
◯ Mauritania ◯ Mauritius
◯ Mexico ◯ Micronesia
◯ Moldova ◯ Monaco
◯ Mongolia ◯ Montenegro
◯ Morocco ◯ Mozambique
◯ Myanmar (Burma) ◯ Namibia
◯ Nauru ◯ Nepal ◯ Netherlands
◯ New Zealand ◯ Nicaragua
◯ Niger ◯ Nigeria ◯ North Macedonia
◯ Norway ◯ Oman ◯ Pakistan
◯ Palau ◯ Panama ◯ Papua New Guinea
◯ Paraguay ◯ Peru ◯ Philippines
◯ Poland ◯ Portugal
◯ Qatar ◯ Romania ◯ Russia
◯ Rwanda ◯ St Kitts and Nevis
◯ St Lucia ◯ St Vincent
◯ Samoa ◯ San Marino
◯ Sao Tome and Principe
◯ Saudi Arabia ◯ Senegal
◯ Serbia ◯ Seychelles

◯ Sierra Leone ◯ Singapore
◯ Slovakia ◯ Slovenia
◯ Solomon Islands ◯ Somalia
◯ South Africa ◯ South Sudan
◯ Spain ◯ Sri Lanka
◯ Sudan ◯ Suriname
◯ Sweden ◯ Switzerland
◯ Syria ◯ Taiwan ◯ Tajikistan
◯ Tanzania ◯ Thailand
◯ Togo ◯ Tonga ◯ Trinidad and Tobago
◯ Tunisia ◯ Turkey ◯ Turkmenistan
◯ Tuvalu ◯ Uganda ◯ Ukraine
◯ United Arab Emirates
◯ United States ◯ Uruguay
◯ Uzbekistan ◯ Vanuatu
◯ Vatican City ◯ Venezuela
◯ Vietnam ◯ Yemen
◯ Zambia ◯ Zimbabwe
◯ Other (Please specify)
◯ Prefer not to answer

Please specify nationality 1: ______________________________

Confidential

Nationality 2:

◯ United Kingdom ◯ Afghanistan
◯ Albania ◯ Algeria ◯ Andorra
◯ Angola ◯ Antigua and Barbuda
◯ Argentina ◯ Armenia
◯ Australia ◯ Austria
◯ Azerbaijan ◯ Bahamas, The
◯ Bahrain ◯ Bangladesh
◯ Barbados ◯ Belarus
◯ Belgium ◯ Belize ◯ Benin
◯ Bhutan ◯ Bolivia ◯ Bosnia and Herzegovina ◯ Botswana
◯ Brazil ◯ Brunei ◯ Bulgaria
◯ Burkina Faso ◯ Burundi
◯ Cambodia ◯ Cameroon
◯ Canada ◯ Cape Verde
◯ Central African Republic
◯ Chad ◯ Chile ◯ China
◯ Colombia ◯ Comoros
◯ Congo ◯ Congo (Democratic Republic)
◯ Costa Rica ◯ Croatia
◯ Cuba ◯ Cyprus ◯ Czechia
◯ Denmark ◯ Djibouti
◯ Dominica ◯ Dominican Republic
◯ East Timor ◯ Ecuador
◯ Egypt ◯ El Salvador
◯ Equatorial Guinea ◯ Eritrea
◯ Estonia ◯ Eswatini
◯ Ethiopia ◯ Fiji ◯ Finland
◯ France ◯ Gabon ◯ Gambia, The
◯ Georgia ◯ Germany
◯ Ghana ◯ Greece ◯ Grenada
◯ Guatemala ◯ Guinea Republic of Guinea
◯ Guinea-Bissau ◯ Guyana
◯ Haiti ◯ Honduras ◯ Hungary
◯ Iceland ◯ India ◯ Indonesia
◯ Iran ◯ Iraq ◯ Ireland
◯ Israel ◯ Italy ◯ Ivory Coast
◯ Jamaica ◯ Japan ◯ Jordan
◯ Kazakhstan ◯ Kenya
◯ Kiribati ◯ Korea (North)
◯ Korea (South) ◯ Kosovo
◯ Kuwait ◯ Kyrgyzstan
◯ Laos ◯ Latvia ◯ Lebanon
◯ Lesotho ◯ Liberia
◯ Libya ◯ Liechtenstein
◯ Lithuania ◯ Luxembourg
◯ Madagascar ◯ Malawi
◯ Malaysia ◯ Maldives
◯ Mali ◯ Malta ◯ Marshall Islands
◯ Mauritania ◯ Mauritius
◯ Mexico ◯ Micronesia
◯ Moldova ◯ Monaco
◯ Mongolia ◯ Montenegro
◯ Morocco ◯ Mozambique
◯ Myanmar (Burma) ◯ Namibia
◯ Nauru ◯ Nepal ◯ Netherlands
◯ New Zealand ◯ Nicaragua
◯ Niger ◯ Nigeria ◯ North Macedonia
◯ Norway ◯ Oman ◯ Pakistan
◯ Palau ◯ Panama ◯ Papua New Guinea
◯ Paraguay ◯ Peru ◯ Philippines
◯ Poland ◯ Portugal
◯ Qatar ◯ Romania ◯ Russia
◯ Rwanda ◯ St Kitts and Nevis
◯ St Lucia ◯ St Vincent
◯ Samoa ◯ San Marino
◯ Sao Tome and Principe
◯ Saudi Arabia ◯ Senegal
◯ Serbia ◯ Seychelles

◯ Sierra Leone ◯ Singapore
◯ Slovakia ◯ Slovenia
◯ Solomon Islands ◯ Somalia
◯ South Africa ◯ South Sudan
◯ Spain ◯ Sri Lanka
◯ Sudan ◯ Suriname
◯ Sweden ◯ Switzerland
◯ Syria ◯ Taiwan ◯ Tajikistan
◯ Tanzania ◯ Thailand
◯ Togo ◯ Tonga ◯ Trinidad and Tobago
◯ Tunisia ◯ Turkey ◯ Turkmenistan
◯ Tuvalu ◯ Uganda ◯ Ukraine
◯ United Arab Emirates
◯ United States ◯ Uruguay
◯ Uzbekistan ◯ Vanuatu
◯ Vatican City ◯ Venezuela
◯ Vietnam ◯ Yemen
◯ Zambia ◯ Zimbabwe
◯ Other (Please specify)
◯ Prefer not to answer

Please specify nationality 2: ______________________________

Nationality 3:	◯ United Kingdom ◯ Afghanistan ◯ Albania ◯ Algeria ◯ Andorra ◯ Angola ◯ Antigua and Barbuda ◯ Argentina ◯ Armenia ◯ Australia ◯ Austria ◯ Azerbaijan ◯ Bahamas, The ◯ Bahrain ◯ Bangladesh ◯ Barbados ◯ Belarus ◯ Belgium ◯ Belize ◯ Benin ◯ Bhutan ◯ Bolivia ◯ Bosnia and Herzegovina ◯ Botswana ◯ Brazil ◯ Brunei ◯ Bulgaria ◯ Burkina Faso ◯ Burundi ◯ Cambodia ◯ Cameroon ◯ Canada ◯ Cape Verde ◯ Central African Republic ◯ Chad ◯ Chile ◯ China ◯ Colombia ◯ Comoros ◯ Congo ◯ Congo (Democratic Republic) ◯ Costa Rica ◯ Croatia ◯ Cuba ◯ Cyprus ◯ Czechia ◯ Denmark ◯ Djibouti ◯ Dominica ◯ Dominican Republic ◯ East Timor ◯ Ecuador ◯ Egypt ◯ El Salvador ◯ Equatorial Guinea ◯ Eritrea ◯ Estonia ◯ Eswatini ◯ Ethiopia ◯ Fiji ◯ Finland ◯ France ◯ Gabon ◯ Gambia, The ◯ Georgia ◯ Germany ◯ Ghana ◯ Greece ◯ Grenada ◯ Guatemala ◯ Guinea Republic of Guinea ◯ Guinea-Bissau ◯ Guyana ◯ Haiti ◯ Honduras ◯ Hungary ◯ Iceland ◯ India ◯ Indonesia ◯ Iran ◯ Iraq ◯ Ireland ◯ Israel ◯ Italy ◯ Ivory Coast ◯ Jamaica ◯ Japan ◯ Jordan ◯ Kazakhstan ◯ Kenya ◯ Kiribati ◯ Korea (North) ◯ Korea (South) ◯ Kosovo ◯ Kuwait ◯ Kyrgyzstan ◯ Laos ◯ Latvia ◯ Lebanon ◯ Lesotho ◯ Liberia ◯ Libya ◯ Liechtenstein ◯ Lithuania ◯ Luxembourg ◯ Madagascar ◯ Malawi ◯ Malaysia ◯ Maldives ◯ Mali ◯ Malta ◯ Marshall Islands ◯ Mauritania ◯ Mauritius ◯ Mexico ◯ Micronesia ◯ Moldova ◯ Monaco ◯ Mongolia ◯ Montenegro ◯ Morocco ◯ Mozambique ◯ Myanmar (Burma) ◯ Namibia ◯ Nauru ◯ Nepal ◯ Netherlands ◯ New Zealand ◯ Nicaragua ◯ Niger ◯ Nigeria ◯ North Macedonia ◯ Norway ◯ Oman ◯ Pakistan ◯ Palau ◯ Panama ◯ Papua New Guinea ◯ Paraguay ◯ Peru ◯ Philippines ◯ Poland ◯ Portugal ◯ Qatar ◯ Romania ◯ Russia ◯ Rwanda ◯ St Kitts and Nevis ◯ St Lucia ◯ St Vincent ◯ Samoa ◯ San Marino ◯ Sao Tome and Principe ◯ Saudi Arabia ◯ Senegal ◯ Serbia ◯ Seychelles

	◯ Sierra Leone ◯ Singapore<br>◯ Slovakia ◯ Slovenia<br>◯ Solomon Islands ◯ Somalia<br>◯ South Africa ◯ South Sudan<br>◯ Spain ◯ Sri Lanka<br>◯ Sudan ◯ Suriname<br>◯ Sweden ◯ Switzerland<br>◯ Syria ◯ Taiwan ◯ Tajikistan<br>◯ Tanzania ◯ Thailand<br>◯ Togo ◯ Tonga ◯ Trinidad and Tobago<br>◯ Tunisia ◯ Turkey ◯ Turkmenistan<br>◯ Tuvalu ◯ Uganda ◯ Ukraine<br>◯ United Arab Emirates<br>◯ United States ◯ Uruguay<br>◯ Uzbekistan ◯ Vanuatu<br>◯ Vatican City ◯ Venezuela<br>◯ Vietnam ◯ Yemen<br>◯ Zambia ◯ Zimbabwe<br>◯ Other (Please specify)<br>◯ Prefer not to answer
Please specify nationality 3:	__________________________________
What is or was your partner's ethnic group?<br><br>The following categories are the ethnic groups used in the UK National Census.	◯ Asian/Asian British - Indian<br>◯ Asian/Asian British - Pakistani<br>◯ Asian/Asian British - Bangladeshi<br>◯ Asian/Asian British - Chinese<br>◯ Asian/Asian British - Any other Asian background<br>◯ Black/African/Caribbean/Black British - African<br>◯ Black/African/Caribbean/Black British - Caribbean<br>◯ Black/African/Caribbean/Black British - Any other Black/African/Caribbean background<br>◯ Mixed/Multiple ethnic groups - White and Black Caribbean<br>◯ Mixed/Multiple ethnic groups - White and Black African<br>◯ Mixed/Multiple ethnic groups - White and Asian<br>◯ Mixed/Multiple ethnic groups - Any other Mixed/multiple ethnic background<br>◯ White - English/Welsh/Scottish/Northern Irish/British<br>◯ White - Irish<br>◯ White - Gypsy or Irish Traveller<br>◯ White - Any other white background<br>◯ Other ethnic group - Arab<br>◯ Other ethnic group - Any other ethnic background (please specify)<br>◯ Prefer not to say
Please specify your partner's ethnic group:	__________________________________

Confidential

What is or was your mother's ethnic group?<br><br>The following categories are the ethnic groups used in the UK National Census.	◯ Asian/Asian British - Indian<br>◯ Asian/Asian British - Pakistani<br>◯ Asian/Asian British - Bangladeshi<br>◯ Asian/Asian British - Chinese<br>◯ Asian/Asian British - Any other Asian background<br>◯ Black/African/Caribbean/Black British - African<br>◯ Black/African/Caribbean/Black British - Caribbean<br>◯ Black/African/Caribbean/Black British - Any other Black/African/Caribbean background<br>◯ Mixed/Multiple ethnic groups - White and Black Caribbean<br>◯ Mixed/Multiple ethnic groups - White and Black African<br>◯ Mixed/Multiple ethnic groups - White and Asian<br>◯ Mixed/Multiple ethnic groups - Any other Mixed/multiple ethnic background<br>◯ White - English/Welsh/Scottish/Northern Irish/British<br>◯ White - Irish<br>◯ White - Gypsy or Irish Traveller<br>◯ White - Any other white background<br>◯ Other ethnic group - Arab<br>◯ Other ethnic group - Any other ethnic background (please specify)<br>◯ Prefer not to say
Please specify your mother's ethnic group:	__________________________________
Was your mother born in the UK?	◯ No<br>◯ Yes<br>◯ Do not know<br>◯ Prefer not to answer

Woolf K, *et al. BMJ Open* 2021; 11:e050647. doi: 10.1136/bmjopen-2021-050647

Confidential

In which country was your mother born?

◯ Afghanistan
◯ Albania
◯ Algeria
◯ Andorra
◯ Angola
◯ Antigua and Barbuda
◯ Argentina
◯ Armenia
◯ Australia
◯ Austria
◯ Azerbaijan
◯ Bahamas, The
◯ Bahrain
◯ Bangladesh
◯ Barbados
◯ Belarus
◯ Belgium
◯ Belize
◯ Benin
◯ Bhutan
◯ Bolivia
◯ Bosnia and Herzegovina
◯ Botswana
◯ Brazil
◯ Brunei
◯ Bulgaria
◯ Burkina Faso
◯ Burundi
◯ Cambodia
◯ Cameroon
◯ Canada
◯ Cape Verde
◯ Central African Republic
◯ Chad
◯ Chile
◯ China
◯ Colombia
◯ Comoros
◯ Congo
◯ Congo (Democratic Republic)
◯ Costa Rica
◯ Croatia
◯ Cuba
◯ Cyprus
◯ Czechia
◯ Denmark
◯ Djibouti
◯ Dominica
◯ Dominican Republic
◯ East Timor
◯ Ecuador
◯ Egypt
◯ El Salvador
◯ Equatorial Guinea
◯ Eritrea
◯ Estonia
◯ Eswatini
◯ Ethiopia
◯ Fiji
◯ Finland
◯ France
◯ Gabon
◯ Gambia, The
◯ Georgia
◯ Germany
◯ Ghana
◯ Greece
◯ Grenada
◯ Guatemala

◯ Guinea Republic of Guinea
◯ Guinea-Bissau
◯ Guyana
◯ Haiti
◯ Honduras
◯ Hungary
◯ Iceland
◯ India
◯ Indonesia
◯ Iran
◯ Iraq
◯ Ireland
◯ Israel
◯ Italy
◯ Ivory Coast
◯ Jamaica
◯ Japan
◯ Jordan
◯ Kazakhstan
◯ Kenya
◯ Kiribati
◯ Korea (North)
◯ Korea (South)
◯ Kosovo
◯ Kuwait
◯ Kyrgyzstan
◯ Laos
◯ Latvia
◯ Lebanon
◯ Lesotho
◯ Liberia
◯ Libya
◯ Liechtenstein
◯ Lithuania
◯ Luxembourg
◯ Madagascar
◯ Malawi
◯ Malaysia
◯ Maldives
◯ Mali
◯ Malta
◯ Marshall Islands
◯ Mauritania
◯ Mauritius
◯ Mexico
◯ Micronesia
◯ Moldova
◯ Monaco
◯ Mongolia
◯ Montenegro
◯ Morocco
◯ Mozambique
◯ Myanmar (Burma)
◯ Namibia
◯ Nauru
◯ Nepal
◯ Netherlands
◯ New Zealand
◯ Nicaragua
◯ Niger
◯ Nigeria
◯ North Macedonia
◯ Norway
◯ Oman
◯ Pakistan
◯ Palau
◯ Panama
◯ Papua New Guinea
◯ Paraguay
◯ Peru
◯ Philippines

◯ Poland
◯ Portugal
◯ Qatar
◯ Romania
◯ Russia
◯ Rwanda
◯ St Kitts and Nevis
◯ St Lucia
◯ St Vincent
◯ Samoa
◯ San Marino
◯ Sao Tome and Principe
◯ Saudi Arabia
◯ Senegal
◯ Serbia
◯ Seychelles
◯ Sierra Leone
◯ Singapore
◯ Slovakia
◯ Slovenia
◯ Solomon Islands
◯ Somalia
◯ South Africa
◯ South Sudan
◯ Spain
◯ Sri Lanka
◯ Sudan
◯ Suriname
◯ Sweden
◯ Switzerland
◯ Syria
◯ Taiwan
◯ Tajikistan
◯ Tanzania
◯ Thailand
◯ Togo
◯ Tonga
◯ Trinidad and Tobago
◯ Tunisia
◯ Turkey
◯ Turkmenistan
◯ Tuvalu
◯ Uganda
◯ Ukraine
◯ United Arab Emirates
◯ United States
◯ Uruguay
◯ Uzbekistan
◯ Vanuatu
◯ Vatican City
◯ Venezuela
◯ Vietnam
◯ Yemen
◯ Zambia
◯ Zimbabwe
◯ Other (Please specify)
◯ Prefer not to answer

Please specify the country in which your mother was born: __________________________

Confidential

What is or was your father's ethnic group?<br><br>The following categories are the ethnic groups used in the UK National Census.	◯ Asian/Asian British - Indian<br>◯ Asian/Asian British - Pakistani<br>◯ Asian/Asian British - Bangladeshi<br>◯ Asian/Asian British - Chinese<br>◯ Asian/Asian British - Any other Asian background<br>◯ Black/African/Caribbean/Black British - African<br>◯ Black/African/Caribbean/Black British - Caribbean<br>◯ Black/African/Caribbean/Black British - Any other Black/African/Caribbean background<br>◯ Mixed/Multiple ethnic groups - White and Black Caribbean<br>◯ Mixed/Multiple ethnic groups - White and Black African<br>◯ Mixed/Multiple ethnic groups - White and Asian<br>◯ Mixed/Multiple ethnic groups - Any other Mixed/multiple ethnic background<br>◯ White - English/Welsh/Scottish/Northern Irish/British<br>◯ White - Irish<br>◯ White - Gypsy or Irish Traveller<br>◯ White - Any other white background<br>◯ Other ethnic group - Arab<br>◯ Other ethnic group - Any other ethnic background (please specify)<br>◯ Prefer not to say
Please specify your father's ethnic group:	__________________________________
Was your father born in the UK?	◯ No<br>◯ Yes<br>◯ Do not know<br>◯ Prefer not to answer

Woolf K, *et al. BMJ Open* 2021; 11:e050647. doi: 10.1136/bmjopen-2021-050647

In which country was your father born?

◯ Afghanistan
◯ Albania
◯ Algeria
◯ Andorra
◯ Angola
◯ Antigua and Barbuda
◯ Argentina
◯ Armenia
◯ Australia
◯ Austria
◯ Azerbaijan
◯ Bahamas, The
◯ Bahrain
◯ Bangladesh
◯ Barbados
◯ Belarus
◯ Belgium
◯ Belize
◯ Benin
◯ Bhutan
◯ Bolivia
◯ Bosnia and Herzegovina
◯ Botswana
◯ Brazil
◯ Brunei
◯ Bulgaria
◯ Burkina Faso
◯ Burundi
◯ Cambodia
◯ Cameroon
◯ Canada
◯ Cape Verde
◯ Central African Republic
◯ Chad
◯ Chile
◯ China
◯ Colombia
◯ Comoros
◯ Congo
◯ Congo (Democratic Republic)
◯ Costa Rica
◯ Croatia
◯ Cuba
◯ Cyprus
◯ Czechia
◯ Denmark
◯ Djibouti
◯ Dominica
◯ Dominican Republic
◯ East Timor
◯ Ecuador
◯ Egypt
◯ El Salvador
◯ Equatorial Guinea
◯ Eritrea
◯ Estonia
◯ Eswatini
◯ Ethiopia
◯ Fiji
◯ Finland
◯ France
◯ Gabon
◯ Gambia, The
◯ Georgia
◯ Germany
◯ Ghana
◯ Greece
◯ Grenada
◯ Guatemala

◯ Guinea Republic of Guinea
◯ Guinea-Bissau
◯ Guyana
◯ Haiti
◯ Honduras
◯ Hungary
◯ Iceland
◯ India
◯ Indonesia
◯ Iran
◯ Iraq
◯ Ireland
◯ Israel
◯ Italy
◯ Ivory Coast
◯ Jamaica
◯ Japan
◯ Jordan
◯ Kazakhstan
◯ Kenya
◯ Kiribati
◯ Korea (North)
◯ Korea (South)
◯ Kosovo
◯ Kuwait
◯ Kyrgyzstan
◯ Laos
◯ Latvia
◯ Lebanon
◯ Lesotho
◯ Liberia
◯ Libya
◯ Liechtenstein
◯ Lithuania
◯ Luxembourg
◯ Madagascar
◯ Malawi
◯ Malaysia
◯ Maldives
◯ Mali
◯ Malta
◯ Marshall Islands
◯ Mauritania
◯ Mauritius
◯ Mexico
◯ Micronesia
◯ Moldova
◯ Monaco
◯ Mongolia
◯ Montenegro
◯ Morocco
◯ Mozambique
◯ Myanmar (Burma)
◯ Namibia
◯ Nauru
◯ Nepal
◯ Netherlands
◯ New Zealand
◯ Nicaragua
◯ Niger
◯ Nigeria
◯ North Macedonia
◯ Norway
◯ Oman
◯ Pakistan
◯ Palau
◯ Panama
◯ Papua New Guinea
◯ Paraguay
◯ Peru
◯ Philippines

◯ Poland
◯ Portugal
◯ Qatar
◯ Romania
◯ Russia
◯ Rwanda
◯ St Kitts and Nevis
◯ St Lucia
◯ St Vincent
◯ Samoa
◯ San Marino
◯ Sao Tome and Principe
◯ Saudi Arabia
◯ Senegal
◯ Serbia
◯ Seychelles
◯ Sierra Leone
◯ Singapore
◯ Slovakia
◯ Slovenia
◯ Solomon Islands
◯ Somalia
◯ South Africa
◯ South Sudan
◯ Spain
◯ Sri Lanka
◯ Sudan
◯ Suriname
◯ Sweden
◯ Switzerland
◯ Syria
◯ Taiwan
◯ Tajikistan
◯ Tanzania
◯ Thailand
◯ Togo
◯ Tonga
◯ Trinidad and Tobago
◯ Tunisia
◯ Turkey
◯ Turkmenistan
◯ Tuvalu
◯ Uganda
◯ Ukraine
◯ United Arab Emirates
◯ United States
◯ Uruguay
◯ Uzbekistan
◯ Vanuatu
◯ Vatican City
◯ Venezuela
◯ Vietnam
◯ Yemen
◯ Zambia
◯ Zimbabwe
◯ Other (Please specify)
◯ Prefer not to answer

Please specify the country in which your father was born: __________________________________

Were your grandparents born in the UK?

Confidential

	NOT born in the UK	Born in the UK	Do not know	Prefer not to answer
Your mother's mother	◯	◯	◯	◯
Your mother's father	◯	◯	◯	◯
Your father's mother	◯	◯	◯	◯
Your father's father	◯	◯	◯	◯

Woolf K, *et al. BMJ Open* 2021; 11:e050647. doi: 10.1136/bmjopen-2021-050647

Section 3/10: Your ethnic, cultural and national identity and background

24% complete
.

How old were you when you first learned English?	◯ Always spoken it<br>◯ 2-5 years<br>◯ 6-10 years<br>◯ 11-17 years<br>◯ 18+<br>◯ Prefer not to answer

What language or languages do you speak most often at home?

If you only speak one language at home, please leave the box for "Language 2" blank.

Woolf K, *et al. BMJ Open* 2021; 11:e050647. doi: 10.1136/bmjopen-2021-050647

Language 1	◯ English<br>◯ Afrikaans<br>◯ Akan<br>◯ Arabic<br>◯ Bengali<br>◯ Bulgarian<br>◯ Burmese<br>◯ Cebuano<br>◯ Chinese<br>◯ Czech<br>◯ Dutch<br>◯ French<br>◯ German<br>◯ Greek<br>◯ Gujarati<br>◯ Hausa<br>◯ Hindi<br>◯ Hungarian<br>◯ Igbo<br>◯ Irish<br>◯ Italian<br>◯ Kurdish<br>◯ Latvian<br>◯ Lithuanian<br>◯ Malay<br>◯ Malayalam<br>◯ Maltese<br>◯ Maori<br>◯ Ndebele<br>◯ Nepali<br>◯ Persian/Farsi<br>◯ Polish<br>◯ Portuguese<br>◯ Punjabi<br>◯ Romanian<br>◯ Russian<br>◯ Shona<br>◯ Sinhala<br>◯ Slovakian<br>◯ Somali<br>◯ Spanish<br>◯ Swahili<br>◯ Swedish<br>◯ Tagalog<br>◯ Tamil<br>◯ Turkish<br>◯ Twi<br>◯ Ukrainian<br>◯ Urdu<br>◯ Welsh<br>◯ Xhosa<br>◯ Yoruba<br>◯ Zulu<br>◯ Other (Please specify)<br>◯ Prefer not to answer
Please specify language 1:	__________________________________

Language 2	◯ English<br>◯ Afrikaans<br>◯ Akan<br>◯ Arabic<br>◯ Bengali<br>◯ Bulgarian<br>◯ Burmese<br>◯ Cebuano<br>◯ Chinese<br>◯ Czech<br>◯ Dutch<br>◯ French<br>◯ German<br>◯ Greek<br>◯ Gujarati<br>◯ Hausa<br>◯ Hindi<br>◯ Hungarian<br>◯ Igbo<br>◯ Irish<br>◯ Italian<br>◯ Kurdish<br>◯ Latvian<br>◯ Lithuanian<br>◯ Malay<br>◯ Malayalam<br>◯ Maltese<br>◯ Maori<br>◯ Ndebele<br>◯ Nepali<br>◯ Persian/Farsi<br>◯ Polish<br>◯ Portuguese<br>◯ Punjabi<br>◯ Romanian<br>◯ Russian<br>◯ Shona<br>◯ Sinhala<br>◯ Slovakian<br>◯ Somali<br>◯ Spanish<br>◯ Swahili<br>◯ Swedish<br>◯ Tagalog<br>◯ Tamil<br>◯ Turkish<br>◯ Twi<br>◯ Ukrainian<br>◯ Urdu<br>◯ Welsh<br>◯ Xhosa<br>◯ Yoruba<br>◯ Zulu<br>◯ Other (Please specify)<br>◯ Prefer not to answer
Please specify language 2:	__________________________________

Woolf K, *et al. BMJ Open* 2021; 11:e050647. doi: 10.1136/bmjopen-2021-050647

What language was spoken in your home when you were a child?<br>If more than one language was spoken, please give them all.	☐ English<br>☐ Afrikaans<br>☐ Akan<br>☐ Arabic<br>☐ Bengali<br>☐ Bulgarian<br>☐ Burmese<br>☐ Cebuano<br>☐ Chinese<br>☐ Czech<br>☐ Dutch<br>☐ French<br>☐ German<br>☐ Greek<br>☐ Gujarati<br>☐ Hausa<br>☐ Hindi<br>☐ Hungarian<br>☐ Igbo<br>☐ Irish<br>☐ Italian<br>☐ Kurdish<br>☐ Latvian<br>☐ Lithuanian<br>☐ Malay<br>☐ Malayalam<br>☐ Maltese<br>☐ Maori<br>☐ Ndebele<br>☐ Nepali<br>☐ Persian/Farsi<br>☐ Polish<br>☐ Portuguese<br>☐ Punjabi<br>☐ Romanian<br>☐ Russian<br>☐ Shona<br>☐ Sinhala<br>☐ Slovakian<br>☐ Somali<br>☐ Spanish<br>☐ Swahili<br>☐ Swedish<br>☐ Tagalog<br>☐ Tamil<br>☐ Turkish<br>☐ Twi<br>☐ Ukrainian<br>☐ Urdu<br>☐ Welsh<br>☐ Xhosa<br>☐ Yoruba<br>☐ Zulu<br>☐ Other (Please specify)<br>☐ Prefer not to answer
Please specify the language spoken in your home when you were a child for which you selected "Other":	________________________________

Confidential

Section 3/10: Your ethnic, cultural and national identity and background

26% complete
.

What is your religion?<br><br>This is the question that was asked in the 2011 UK Census.	◯ No religion<br>◯ Christian (including Church of England, Catholic, Protestant and all other Christian denominations)<br>◯ Buddhist<br>◯ Hindu<br>◯ Jewish<br>◯ Muslim<br>◯ Sikh<br>◯ Any other religion (please specify)<br>◯ Prefer not to answer
Please specify your religion:	______________________________
How important is religion to you in your everyday life?	◯ Not at all important<br>◯ Fairly important<br>◯ Very important<br>◯ Extremely important<br>◯ Prefer not to answer
How important was religion in your upbringing?	◯ Not at all important<br>◯ Fairly important<br>◯ Very important<br>◯ Extremely important<br>◯ Prefer not to answer
How often would you usually attend a holy place or a place of worship outside your home?	◯ Never<br>◯ On festive occasions only<br>◯ Once every few months<br>◯ About once a month<br>◯ Most weeks<br>◯ More than once a week<br>◯ Prefer not to answer

Section 3/10: Your ethnic, cultural and national identity and background

28% complete
.

How important is your ethnic and cultural background to your identity?<br><br>Use the scale of 0 to 10, where 0 means 'not at all important', and 10 means 'extremely important'.	◯ 0 Not at all important<br>◯ 1<br>◯ 2<br>◯ 3<br>◯ 4<br>◯ 5<br>◯ 6<br>◯ 7<br>◯ 8<br>◯ 9<br>◯ 10 Extremely important<br>◯ Prefer not to answer
How important is/was your mother's ethnic and cultural background to your identity?<br><br>Use the scale of 0 to 10, where 0 means 'not at all important', and 10 means 'extremely important'.	◯ 0 Not at all important<br>◯ 1<br>◯ 2<br>◯ 3<br>◯ 4<br>◯ 5<br>◯ 6<br>◯ 7<br>◯ 8<br>◯ 9<br>◯ 10 Extremely important<br>◯ Prefer not to answer
How important is/was your father's ethnic and cultural background to your identity?<br><br>Use the scale of 0 to 10, where 0 means 'not at all important', and 10 means 'extremely important'.	◯ 0 Not at all important<br>◯ 1<br>◯ 2<br>◯ 3<br>◯ 4<br>◯ 5<br>◯ 6<br>◯ 7<br>◯ 8<br>◯ 9<br>◯ 10 Extremely important<br>◯ Prefer not to answer
How important is/was your partner's ethnic and cultural background to your identity?<br><br>Use the scale of 0 to 10, where 0 means 'not at all important', and 10 means 'extremely important'.	◯ 0 Not at all important<br>◯ 1<br>◯ 2<br>◯ 3<br>◯ 4<br>◯ 5<br>◯ 6<br>◯ 7<br>◯ 8<br>◯ 9<br>◯ 10 Extremely important<br>◯ Prefer not to answer

Section 3/10: Your ethnic, cultural and national identity and background

30% complete

.

In which country did you gain your primary professional qualification?<br><br>(The qualification used for registration with your professional regulator, such as the GDC, GMC, GOC, GPhC, PSNI, HCPC, NMC)	◯ Not applicable - I do not hold a professional qualification<br>◯ UK<br>◯ Australia<br>◯ Bangladesh<br>◯ Bulgaria<br>◯ Canada<br>◯ China<br>◯ Czech Republic<br>◯ Egypt<br>◯ France<br>◯ Germany<br>◯ Ghana<br>◯ Greece<br>◯ Hungary<br>◯ India<br>◯ Iran<br>◯ Iraq<br>◯ Ireland<br>◯ Italy<br>◯ Jamaica<br>◯ Kenya<br>◯ Latvia<br>◯ Libya<br>◯ Lithuania<br>◯ Malaysia<br>◯ Malta<br>◯ Mauritius<br>◯ Myanmar<br>◯ Nepal<br>◯ Netherlands<br>◯ New Zealand<br>◯ Nigeria<br>◯ Pakistan<br>◯ Philippines<br>◯ Poland<br>◯ Portugal<br>◯ Romania<br>◯ Russia<br>◯ Slovakia<br>◯ Somalia<br>◯ South Africa<br>◯ Spain<br>◯ Sri Lanka<br>◯ Sudan<br>◯ Sweden<br>◯ Syria<br>◯ Trinidad and Tobago<br>◯ Turkey<br>◯ Uganda<br>◯ Ukraine<br>◯ USA<br>◯ Zimbabwe<br>◯ Other country (please specify)<br>◯ Prefer not to answer
Please specify the country in which you gained your primary professional qualification:	__________________________________
In which year did you obtain your primary professional qualification?	__________________________________<br>((Please enter in format YYYY, e.g. 1990))

What is the highest level of education you have completed?	◯ Primary (up to 11 years)<br>◯ Secondary (11 to 16 years)<br>◯ Post-secondary (16-18 years)<br>◯ Other qualifications below degree level (e.g. nursing diploma)<br>◯ Undergraduate degree or equivalent (e.g. BA, BSc, medical or nursing degree)<br>◯ Masters degree or equivalent (e.g. MSc, Ma)<br>◯ Doctorate level (e.g. PhD, MD)<br>◯ Prefer not to answer
What is the highest level of education your mother has completed?	◯ Primary (up to 11 years)<br>◯ Secondary (11 to 16 years)<br>◯ Post-secondary (16-18 years)<br>◯ Other qualifications below degree level (e.g. nursing diploma)<br>◯ Undergraduate degree or equivalent (e.g. BA, BSc, medical or nursing degree)<br>◯ Masters degree or equivalent (e.g. MSc, Ma)<br>◯ Doctorate level (e.g. PhD, MD)<br>◯ Prefer not to answer
What is the highest level of education your father has completed?	◯ Primary (up to 11 years)<br>◯ Secondary (11 to 16 years)<br>◯ Post-secondary (16-18 years)<br>◯ Other qualifications below degree level (e.g. nursing diploma)<br>◯ Undergraduate degree or equivalent (e.g. BA, BSc, medical or nursing degree)<br>◯ Masters degree or equivalent (e.g. MSc, Ma)<br>◯ Doctorate level (e.g. PhD, MD)<br>◯ Prefer not to answer

Confidential

Section 3/10: Your ethnic, cultural and national identity and background

33% complete
.

In your current main job/role, what proportion of colleagues who are senior to you are of the same ethnic group as yourself?

- ◯ None
- ◯ Hardly any
- ◯ Some
- ◯ About a half
- ◯ Most
- ◯ Almost all
- ◯ All
- ◯ Prefer not to answer

In your current main job/role, what proportion of your colleagues who are senior to you are White?

- ◯ None
- ◯ Hardly any
- ◯ Some
- ◯ About a half
- ◯ Most
- ◯ Almost all
- ◯ All
- ◯ Prefer not to answer

Thinking about where you work in your current main job/role, does your organisation act fairly with regard to career progression / promotion, regardless of ethnic background, gender, religion, sexual orientation, disability or age?

- ◯ Not at all
- ◯ Rarely
- ◯ Sometimes
- ◯ Mostly
- ◯ Definitely
- ◯ Prefer not to answer

Woolf K, *et al. BMJ Open* 2021; 11:e050647. doi: 10.1136/bmjopen-2021-050647

Confidential

Section 4/10: Your home and family life

35% complete
.

This section asks about your home and the people who live with you, to help us to understand more about how things outside work may affect COVID-19 risk.

Woolf K, *et al. BMJ Open* 2021; 11:e050647. doi: 10.1136/bmjopen-2021-050647

Section 4/10: Your home and family life

35% complete
.

The following questions may refer to your household.

A household is one person living alone, or a group of people (not necessarily related) living at the same address who share cooking facilities and share a living room, sitting room or dining area.

A household can consist of a single family, more than one family or no families in the case of a group of unrelated people.
Your household does not include anyone you may be in a support or childcare bubble with who live at a different address to you.

Do you have a “support bubble” (in England or Northern Ireland) or “extended household” (in Wales or Scotland) which includes people who usually live at a different address?	◯ No<br>◯ Yes<br>◯ Do not know<br>◯ Prefer not to answer
How many people are in this "support bubble"?<br>Only count those who usually live at a different address.	__________________________
Do you have a “childcare bubble” which includes people who usually live at a different address?<br><br>Do not include anyone already counted in the support bubble in the previous question.	◯ No<br>◯ Yes<br>◯ Do not know<br>◯ Prefer not to answer
How many people are in this "childcare bubble"?<br><br>Only count those who usually live at a different address.	__________________________
Apart from you, how many other people are in your household?	◯ 0<br>◯ 1<br>◯ 2<br>◯ 3<br>◯ 4<br>◯ 5<br>◯ 6<br>◯ 7<br>◯ 8<br>◯ 9<br>◯ 10<br>◯ 11<br>◯ 12<br>◯ 13 or more people<br>◯ Prefer not to answer

Section 4/10: Your home and family life

40% complete
.

These questions are about each person living with you in your current household. We will ask you about each person in turn. It is your choice as to which order you put them in, but it might help to order them by age, from oldest to youngest.

Reminder: A household is one person living alone, or a group of people (not necessarily related) living at the same address who share cooking facilities and share a living room, sitting room or dining area.
A household can consist of a single family, more than one family, or no families in the case of a group of unrelated people.
Your household does not include anyone you may be in a support or childcare bubble with who live at a different address to you.

Section 4/10: Your home and family life

40% complete
.

Person 1 - What best describes this person's relationship to you? Please select from the list provided.	◯ Spouse/Partner<br>◯ Child<br>◯ Grandchild<br>◯ Parent<br>◯ Mother-in-law/Father-in-law/Your partner's parent<br>◯ Twin<br>◯ Sibling (not including your twin)<br>◯ Sister-in-law/Brother-in-law/Your partner's sibling<br>◯ Friend/Housemate<br>◯ Grandparent<br>◯ Aunt/Uncle/Your parent's sibling<br>◯ Great-Aunt/Great-Uncle/Your grandparent's sibling<br>◯ Cousin/Other familial relation<br>◯ Colleague<br>◯ Other<br>◯ Prefer not to answer
Person 1 - How old is this person?	◯ 0-1<br>◯ 2-4<br>◯ 5-10<br>◯ 11-16<br>◯ 17-18<br>◯ 19-24<br>◯ 25-34<br>◯ 35-44<br>◯ 45-54<br>◯ 55-64<br>◯ 65-74<br>◯ 75-84<br>◯ 85-94<br>◯ 95+<br>◯ Prefer not to answer

Confidential

Section 4/10: Your home and family life

42% complete
.

Person 2 - What best describes this person's relationship to you?	◯ Spouse/Partner<br>◯ Child<br>◯ Grandchild<br>◯ Parent<br>◯ Mother-in-law/Father-in-law/Your partner's parent<br>◯ Twin<br>◯ Sibling (not including your twin)<br>◯ Sister-in-law/Brother-in-law/Your partner's sibling<br>◯ Friend/Housemate<br>◯ Grandparent<br>◯ Aunt/Uncle/Your parent's sibling<br>◯ Great-Aunt/Great-Uncle/Your grandparent's sibling<br>◯ Cousin/Other familial relation<br>◯ Colleague<br>◯ Other<br>◯ Prefer not to answer
Person 2 - How old is this person?	◯ 0-1<br>◯ 2-4<br>◯ 5-10<br>◯ 11-16<br>◯ 17-18<br>◯ 19-24<br>◯ 25-34<br>◯ 35-44<br>◯ 45-54<br>◯ 55-64<br>◯ 65-74<br>◯ 75-84<br>◯ 85-94<br>◯ 95+<br>◯ Prefer not to answer

Woolf K, *et al. BMJ Open* 2021; 11:e050647. doi: 10.1136/bmjopen-2021-050647

Section 4/10: Your home and family life

44% complete
.

Person 3 - What best describes this person's relationship to you? Please select from the list provided.	◯ Spouse/Partner<br>◯ Child<br>◯ Grandchild<br>◯ Parent<br>◯ Mother-in-law/Father-in-law/Your partner's parent<br>◯ Twin<br>◯ Sibling (not including your twin)<br>◯ Sister-in-law/Brother-in-law/Your partner's sibling<br>◯ Friend/Housemate<br>◯ Grandparent<br>◯ Aunt/Uncle/Your parent's sibling<br>◯ Great-Aunt/Great-Uncle/Your grandparent's sibling<br>◯ Cousin/Other familial relation<br>◯ Colleague<br>◯ Other<br>◯ Prefer not to answer
Person 3 - How old is this person?	◯ 0-1<br>◯ 2-4<br>◯ 5-10<br>◯ 11-16<br>◯ 17-18<br>◯ 19-24<br>◯ 25-34<br>◯ 35-44<br>◯ 45-54<br>◯ 55-64<br>◯ 65-74<br>◯ 75-84<br>◯ 85-94<br>◯ 95+<br>◯ Prefer not to answer

Woolf K, *et al. BMJ Open* 2021; 11:e050647. doi: 10.1136/bmjopen-2021-050647

Section 4/10: Your home and family life

46% complete
.

Person 4 - What best describes this person's relationship to you? Please select from the list provided.	◯ Spouse/Partner<br>◯ Child<br>◯ Grandchild<br>◯ Parent<br>◯ Mother-in-law/Father-in-law/Your partner's parent<br>◯ Twin<br>◯ Sibling (not including your twin)<br>◯ Sister-in-law/Brother-in-law/Your partner's sibling<br>◯ Friend/Housemate<br>◯ Grandparent<br>◯ Aunt/Uncle/Your parent's sibling<br>◯ Great-Aunt/Great-Uncle/Your grandparent's sibling<br>◯ Cousin/Other familial relation<br>◯ Colleague<br>◯ Other<br>◯ Prefer not to answer
Person 4 - How old is this person?	◯ 0-1<br>◯ 2-4<br>◯ 5-10<br>◯ 11-16<br>◯ 17-18<br>◯ 19-24<br>◯ 25-34<br>◯ 35-44<br>◯ 45-54<br>◯ 55-64<br>◯ 65-74<br>◯ 75-84<br>◯ 85-94<br>◯ 95+<br>◯ Prefer not to answer

Confidential

Section 4/10: Your home and family life

47% complete
.

Person 5 - What best describes this person's relationship to you? Please select from the list provided.

- ◯ Spouse/Partner
- ◯ Child
- ◯ Grandchild
- ◯ Parent
- ◯ Mother-in-law/Father-in-law/Your partner's parent
- ◯ Twin
- ◯ Sibling (not including your twin)
- ◯ Sister-in-law/Brother-in-law/Your partner's sibling
- ◯ Friend/Housemate
- ◯ Grandparent
- ◯ Aunt/Uncle/Your parent's sibling
- ◯ Great-Aunt/Great-Uncle/Your grandparent's sibling
- ◯ Cousin/Other familial relation
- ◯ Colleague
- ◯ Other
- ◯ Prefer not to answer

Person 5 - How old is this person?

- ◯ 0-1
- ◯ 2-4
- ◯ 5-10
- ◯ 11-16
- ◯ 17-18
- ◯ 19-24
- ◯ 25-34
- ◯ 35-44
- ◯ 45-54
- ◯ 55-64
- ◯ 65-74
- ◯ 75-84
- ◯ 85-94
- ◯ 95+
- ◯ Prefer not to answer

Woolf K, *et al. BMJ Open* 2021; 11:e050647. doi: 10.1136/bmjopen-2021-050647

Confidential

Section 4/10: Your home and family life

49% complete
.

Person 6 - What best describes this person's relationship to you? Please select from the list provided.	◯ Spouse/Partner<br>◯ Child<br>◯ Grandchild<br>◯ Parent<br>◯ Mother-in-law/Father-in-law/Your partner's parent<br>◯ Twin<br>◯ Sibling (not including your twin)<br>◯ Sister-in-law/Brother-in-law/Your partner's sibling<br>◯ Friend/Housemate<br>◯ Grandparent<br>◯ Aunt/Uncle/Your parent's sibling<br>◯ Great-Aunt/Great-Uncle/Your grandparent's sibling<br>◯ Cousin/Other familial relation<br>◯ Colleague<br>◯ Other<br>◯ Prefer not to answer
Person 6 - How old is this person?	◯ 0-1<br>◯ 2-4<br>◯ 5-10<br>◯ 11-16<br>◯ 17-18<br>◯ 19-24<br>◯ 25-34<br>◯ 35-44<br>◯ 45-54<br>◯ 55-64<br>◯ 65-74<br>◯ 75-84<br>◯ 85-94<br>◯ 95+<br>◯ Prefer not to answer

Section 4/10: Your home and family life

51% complete
.

Person 7 - What best describes this person's relationship to you? Please select from the list provided.	◯ Spouse/Partner<br>◯ Child<br>◯ Grandchild<br>◯ Parent<br>◯ Mother-in-law/Father-in-law/Your partner's parent<br>◯ Twin<br>◯ Sibling (not including your twin)<br>◯ Sister-in-law/Brother-in-law/Your partner's sibling<br>◯ Friend/Housemate<br>◯ Grandparent<br>◯ Aunt/Uncle/Your parent's sibling<br>◯ Great-Aunt/Great-Uncle/Your grandparent's sibling<br>◯ Cousin/Other familial relation<br>◯ Colleague<br>◯ Other<br>◯ Prefer not to answer
Person 7 - How old is this person?	◯ 0-1<br>◯ 2-4<br>◯ 5-10<br>◯ 11-16<br>◯ 17-18<br>◯ 19-24<br>◯ 25-34<br>◯ 35-44<br>◯ 45-54<br>◯ 55-64<br>◯ 65-74<br>◯ 75-84<br>◯ 85-94<br>◯ 95+<br>◯ Prefer not to answer

Section 4/10: Your home and family life

53% complete
.

Person 8 - What best describes this person's relationship to you? Please select from the list provided.	◯ Spouse/Partner<br>◯ Child<br>◯ Grandchild<br>◯ Parent<br>◯ Mother-in-law/Father-in-law/Your partner's parent<br>◯ Twin<br>◯ Sibling (not including your twin)<br>◯ Sister-in-law/Brother-in-law/Your partner's sibling<br>◯ Friend/Housemate<br>◯ Grandparent<br>◯ Aunt/Uncle/Your parent's sibling<br>◯ Great-Aunt/Great-Uncle/Your grandparent's sibling<br>◯ Cousin/Other familial relation<br>◯ Colleague<br>◯ Other<br>◯ Prefer not to answer
Person 8 - How old is this person?	◯ 0-1<br>◯ 2-4<br>◯ 5-10<br>◯ 11-16<br>◯ 17-18<br>◯ 19-24<br>◯ 25-34<br>◯ 35-44<br>◯ 45-54<br>◯ 55-64<br>◯ 65-74<br>◯ 75-84<br>◯ 85-94<br>◯ 95+<br>◯ Prefer not to answer

Confidential

Section 4/10: Your home and family life

54% complete
.

Person 9 - What best describes this person's relationship to you? Please select from the list provided.	◯ Spouse/Partner<br>◯ Child<br>◯ Grandchild<br>◯ Parent<br>◯ Mother-in-law/Father-in-law/Your partner's parent<br>◯ Twin<br>◯ Sibling (not including your twin)<br>◯ Sister-in-law/Brother-in-law/Your partner's sibling<br>◯ Friend/Housemate<br>◯ Grandparent<br>◯ Aunt/Uncle/Your parent's sibling<br>◯ Great-Aunt/Great-Uncle/Your grandparent's sibling<br>◯ Cousin/Other familial relation<br>◯ Colleague<br>◯ Other<br>◯ Prefer not to answer
Person 9 - How old is this person?	◯ 0-1<br>◯ 2-4<br>◯ 5-10<br>◯ 11-16<br>◯ 17-18<br>◯ 19-24<br>◯ 25-34<br>◯ 35-44<br>◯ 45-54<br>◯ 55-64<br>◯ 65-74<br>◯ 75-84<br>◯ 85-94<br>◯ 95+<br>◯ Prefer not to answer

Confidential

Section 4/10: Your home and family life

56% complete
.

Person 10 - What best describes this person's relationship to you? Please select from the list provided.	◯ Spouse/Partner<br>◯ Child<br>◯ Grandchild<br>◯ Parent<br>◯ Mother-in-law/Father-in-law/Your partner's parent<br>◯ Twin<br>◯ Sibling (not including your twin)<br>◯ Sister-in-law/Brother-in-law/Your partner's sibling<br>◯ Friend/Housemate<br>◯ Grandparent<br>◯ Aunt/Uncle/Your parent's sibling<br>◯ Great-Aunt/Great-Uncle/Your grandparent's sibling<br>◯ Cousin/Other familial relation<br>◯ Colleague<br>◯ Other<br>◯ Prefer not to answer
Person 10 - How old is this person?	◯ 0-1<br>◯ 2-4<br>◯ 5-10<br>◯ 11-16<br>◯ 17-18<br>◯ 19-24<br>◯ 25-34<br>◯ 35-44<br>◯ 45-54<br>◯ 55-64<br>◯ 65-74<br>◯ 75-84<br>◯ 85-94<br>◯ 95+<br>◯ Prefer not to answer

Woolf K, *et al. BMJ Open* 2021; 11:e050647. doi: 10.1136/bmjopen-2021-050647

Section 4/10: Your home and family life

58% complete
.

Question	Options
Person 11 - What best describes this person's relationship to you? Please select from the list provided.	◯ Spouse/Partner<br>◯ Child<br>◯ Grandchild<br>◯ Parent<br>◯ Mother-in-law/Father-in-law/Your partner's parent<br>◯ Twin<br>◯ Sibling (not including your twin)<br>◯ Sister-in-law/Brother-in-law/Your partner's sibling<br>◯ Friend/Housemate<br>◯ Grandparent<br>◯ Aunt/Uncle/Your parent's sibling<br>◯ Great-Aunt/Great-Uncle/Your grandparent's sibling<br>◯ Cousin/Other familial relation<br>◯ Colleague<br>◯ Other<br>◯ Prefer not to answer
Person 11 - How old is this person?	◯ 0-1<br>◯ 2-4<br>◯ 5-10<br>◯ 11-16<br>◯ 17-18<br>◯ 19-24<br>◯ 25-34<br>◯ 35-44<br>◯ 45-54<br>◯ 55-64<br>◯ 65-74<br>◯ 75-84<br>◯ 85-94<br>◯ 95+<br>◯ Prefer not to answer

BMJ Publishing Group Limited (BMJ) disclaims all liability and responsibility arising from any reliance placed on this supplemental material which has been supplied by the author(s)

Section 4/10: Your home and family life

60% complete
.

Person 12 - What best describes this person's relationship to you? Please select from the list provided.	◯ Spouse/Partner<br>◯ Child<br>◯ Grandchild<br>◯ Parent<br>◯ Mother-in-law/Father-in-law/Your partner's parent<br>◯ Twin<br>◯ Sibling (not including your twin)<br>◯ Sister-in-law/Brother-in-law/Your partner's sibling<br>◯ Friend/Housemate<br>◯ Grandparent<br>◯ Aunt/Uncle/Your parent's sibling<br>◯ Great-Aunt/Great-Uncle/Your grandparent's sibling<br>◯ Cousin/Other familial relation<br>◯ Colleague<br>◯ Other<br>◯ Prefer not to answer
Person 12 - How old is this person?	◯ 0-1<br>◯ 2-4<br>◯ 5-10<br>◯ 11-16<br>◯ 17-18<br>◯ 19-24<br>◯ 25-34<br>◯ 35-44<br>◯ 45-54<br>◯ 55-64<br>◯ 65-74<br>◯ 75-84<br>◯ 85-94<br>◯ 95+<br>◯ Prefer not to answer

Woolf K, *et al. BMJ Open* 2021; 11:e050647. doi: 10.1136/bmjopen-2021-050647

Confidential

Section 4/10: Your home and family life

62% complete
.

If you live with more than 12 people, please state the relationship to you and ages for the others in this box separated by a comma, e.g.: ________________________________

Aunt 56, Colleague 25

Confidential

Section 4/10: Your home and family life

62% complete
.

Apart from yourself, how many people in your household travel to work using public transport?	______________________ (Enter the number of people)
Apart from yourself, how many people in your household work in jobs that often bring them into close physical contact (within 2 metres) with others?<br><br>Some examples include: bus driver, carer, cleaner, doctor, supermarket checkout worker, teacher.	______________________ (Enter the number of people)

In which year did you move to your current address?

- ◯ 2021
- ◯ 2020
- ◯ 2019
- ◯ 2018
- ◯ 2017
- ◯ 2016
- ◯ 2015
- ◯ 2014
- ◯ 2013
- ◯ 2012
- ◯ 2011
- ◯ 2010
- ◯ 2009
- ◯ 2008
- ◯ 2007
- ◯ 2006
- ◯ 2005
- ◯ 2004
- ◯ 2003
- ◯ 2002
- ◯ 2001
- ◯ 2000
- ◯ 1999
- ◯ 1998
- ◯ 1997
- ◯ 1996
- ◯ 1995
- ◯ 1994
- ◯ 1993
- ◯ 1992
- ◯ 1991
- ◯ 1990
- ◯ 1989
- ◯ 1988
- ◯ 1987
- ◯ 1986
- ◯ 1985
- ◯ 1984
- ◯ 1983
- ◯ 1982
- ◯ 1981
- ◯ 1980
- ◯ 1979
- ◯ 1978
- ◯ 1977
- ◯ 1976
- ◯ 1975
- ◯ 1974
- ◯ 1973
- ◯ 1972
- ◯ 1971
- ◯ 1970
- ◯ 1969
- ◯ 1968
- ◯ 1967
- ◯ 1966
- ◯ 1965
- ◯ 1964
- ◯ 1963
- ◯ 1962
- ◯ 1961
- ◯ 1960
- ◯ 1959
- ◯ 1958
- ◯ 1957
- ◯ 1956
- ◯ 1955
- ◯ 1954
- ◯ 1953

	◯ 1952<br>◯ 1951<br>◯ 1950<br>◯ 1949<br>◯ 1948<br>◯ 1947<br>◯ 1946<br>◯ 1945<br>◯ 1944<br>◯ 1943<br>◯ 1942<br>◯ 1941<br>◯ 1940<br>◯ 1939<br>◯ 1938<br>◯ 1937<br>◯ 1936<br>◯ 1935<br>◯ 1934<br>◯ 1933<br>◯ 1932<br>◯ 1931<br>◯ 1930<br>◯ 1929<br>◯ 1928<br>◯ 1927<br>◯ 1926<br>◯ 1925<br>◯ 1924<br>◯ 1923<br>◯ 1922<br>◯ 1921<br>◯ 1920<br>◯ Prefer not to answer
In which month did you move to your current address?	◯ Jan<br>◯ Feb<br>◯ Mar<br>◯ Apr<br>◯ May<br>◯ Jun<br>◯ Jul<br>◯ Aug<br>◯ Sep<br>◯ Oct<br>◯ Nov<br>◯ Dec<br>◯ Prefer not to answer
What type of accommodation are you currently living in?	◯ Detached house<br>◯ Semi-detached house<br>◯ Terraced house<br>◯ Flat or apartment<br>◯ Hostel<br>◯ Mobile home or caravan<br>◯ Sheltered house<br>◯ Homeless<br>◯ Other (please specify)<br>◯ Prefer not to answer
Please specify what type of accommodation you live in:	__________________________________

Confidential

Is your current accommodation provided by or linked to your employer, e.g. hospital staff accommodation?	◯ No<br>◯ Yes<br>◯ Prefer not to answer
How many rooms are in your accommodation (not including the kitchen and bathroom(s))?	__________________________
Do you share any of the following rooms with people you do not consider to be a part of your household? You may select more than one answer. If you do not share any of the rooms listed, please select 'None'.	☐ None<br>☐ Kitchen<br>☐ Bathroom<br>☐ Living room, sitting room or dining area<br>☐ Prefer not to answer
Does your accommodation include shared communal areas such as hallways, stairwells or lifts?	◯ No<br>◯ Yes<br>◯ Prefer not to answer
Does your accommodation have a safe outdoor space (e.g., a garden or yard) where you can exercise or relax?	◯ No<br>◯ Yes<br>◯ Prefer not to answer
Is your garden/yard shared with other households or private?	◯ Shared<br>◯ Private<br>◯ Prefer not to answer

Woolf K, *et al. BMJ Open* 2021; 11:e050647. doi: 10.1136/bmjopen-2021-050647

Section 5/10: Your friends and social network

63% complete
.

This section asks questions about your friends, social relationships and social networks.

Confidential

Section 5/10: Your friends and social network

63% complete
.

How many people outside those in your household did you talk with in the last week (but not as a part of your work)
If you are answering this questionnaire on a smartphone, you may find it easier to view by rotating the screen.

	0	1-5	6-20	21-50	51+	Prefer not to answer
Remotely (e.g. over the phone, social media or via video media)	○	○	○	○	○	○
Face-to-face with social distancing	○	○	○	○	○	○
With physical contact (e.g. handshake/hug/kiss, etc)	○	○	○	○	○	○

What proportion of your friends are of the same ethnic group as yourself?

- ○ None
- ○ Hardly any
- ○ Some
- ○ About a half
- ○ Most
- ○ Almost all
- ○ All
- ○ Prefer not to answer

Woolf K, *et al. BMJ Open* 2021; 11:e050647. doi: 10.1136/bmjopen-2021-050647

Section 6/10: Harassment and discrimination

64% complete
.

People from any background can be harassed or discriminated against for many reasons.

This section asks about your experiences of discrimination and harassment. Depending on your experiences, you may find some of the questions upsetting or difficult. You can stop at any time and return later if you wish, or you can choose not to answer a question.

You can find information about sources of support on our website and from the organisations below:
• Victim Support – support for people affected by crime or traumatic events, including hate crime: https://www.victimsupport.org.uk/
• Samaritans – Emotional support for everyone: www.samaritans.org
• Mind – Advice and support for anyone with a mental health problem: www.mind.org.uk

Section 6/10: Harassment and discrimination

64% complete
.

In your day-to-day life, how often do any of the following things happen to you?

If you are answering this questionnaire on a smartphone, you may find it easier to view by rotating the screen.

	Almost everyday	At least once a week	A few times a month	A few times a year	Less than once a year	Never	Prefer not to answer
You are treated with less courtesy than other people are.	○	○	○	○	○	○	○
You are treated with less respect than other people are.	○	○	○	○	○	○	○
You receive poorer service than other people at restaurants or shops.	○	○	○	○	○	○	○
People act as if they think you are not smart.	○	○	○	○	○	○	○
People act as if they are afraid of you.	○	○	○	○	○	○	○
People act as if they think you are dishonest.	○	○	○	○	○	○	○
People act as if they're better than you are.	○	○	○	○	○	○	○
You are called names or insulted.	○	○	○	○	○	○	○
You are threatened or harassed.	○	○	○	○	○	○	○

What do you think are the reasons for these experiences?
Please select all that apply.

- ☐ Your national origins
- ☐ Your gender
- ☐ Your ethnicity
- ☐ Your age
- ☐ Your religion
- ☐ Your height
- ☐ Your weight
- ☐ Your health or disability
- ☐ Your dress
- ☐ Some other aspect of your physical appearance
- ☐ Your sexual orientation
- ☐ Your education or income level
- ☐ Your language or accent
- ☐ Your social class
- ☐ Other (please specify)
- ☐ Prefer not to answer

Please specify what you think is the main reason for these experiences: ________________________________

Confidential

In the last 12 months have you personally experienced discrimination at work from any of the following? Select all that apply.	☐ Patients / service users, their relatives or other members of the public<br>☐ Manager / team leader or other colleagues<br>☐ I have not experienced discrimination at work in the last 12 months<br>☐ I have not worked in the last 12 months<br>☐ Prefer not to answer
On what grounds have you experienced discrimination at work?	☐ Your national origins<br>☐ Your gender<br>☐ Your ethnicity<br>☐ Your age<br>☐ Your religion<br>☐ Your height<br>☐ Your weight<br>☐ Your health or disability<br>☐ Your dress<br>☐ Some other aspect of your physical appearance<br>☐ Your sexual orientation<br>☐ Your education or income level<br>☐ Your language or accent<br>☐ Your social class<br>☐ Other (please specify)<br>☐ Prefer not to answer
Please specify the grounds on which you have experienced discrimination at work:	__________________________________
Did you make a complaint about the discrimination at work?	◯ No<br>◯ No - but I did consider it<br>◯ Yes - informally<br>◯ Yes, I made a formal complaint<br>◯ Prefer not to answer

Section 7/10: Your health

67% complete
.

This section asks about your overall physical and mental health. It will ask about:

• Height and weight
• Cigarette, vaping and alcohol usage
• Exercise and physical activity
• Overall health and specific health problems
• Mental health issues

Section 7/10: Your health

67% complete
.

What is your current height?	◯ Enter height in centimetres<br>◯ Enter height in feet and inches<br>◯ Do not know<br>◯ Prefer not to answer
Please enter your current height to the nearest centimetre:	__________________
Feet	__________________
Inches	__________________
What is your current weight?	◯ Enter weight in kilograms<br>◯ Enter weight in stones and pounds<br>◯ Do not know<br>◯ Prefer not to answer
Please enter your current weight in kilograms:	__________________
Stones	__________________
Pounds	__________________

Section 7/10: Your health

68% complete
.

Do you or have you ever smoked tobacco?	◯ Never<br>◯ Ex-smoker<br>◯ Current smoker<br>◯ Prefer not to answer
Do you currently use an e-cigarette or vape?	◯ Yes<br>◯ No<br>◯ Prefer not to answer
How often do you have a drink containing alcohol?	◯ Never<br>◯ Monthly or less<br>◯ 2-4 times per month<br>◯ 2-3 times per week<br>◯ 4+ times per week<br>◯ Prefer not to answer
How many units of alcohol do you drink in a typical week?<br>If you are unsure, see the guide below.<br><br>Pint of standard strength (3.6%) lager/beer/cider<br>2 units<br>Pint of higher strength (5.2%) lager/beer/cider<br>3 units<br>Medium (175ml) glass of wine<br>2 units<br>Large (250ml) glass of wine<br>3 units<br>Bottle (275ml) of alcopop1<br>1.5 units<br>Single shot (25ml) of spirits (e.g. vodka, whisky, gin, rum)<br>1 unit	◯ 0<br>◯ 1-7<br>◯ 8-14<br>◯ 15-21<br>◯ 22-28<br>◯ 29-35<br>◯ 36-50<br>◯ 51+<br>◯ Prefer not to answer<br>(Select the number of units)

Confidential

Section 7/10: Your health

71% complete
.

Think about a typical week at work over the past month. Please consider the type and amount of physical activity involved in your work.

Please select one option only.

- ◯ I spend most of my time at work sitting
- ◯ I spend most of my time at work standing or walking. However, my work does not require much intense physical effort
- ◯ My work involves definite physical effort including handling of heavy objects and use of tools
- ◯ My work involves vigorous physical activity including handling of very heavy objects
- ◯ I am not in employment
- ◯ Prefer not to answer

During the last week, about how many hours did you spend on each of the following activities?
If you are answering this questionnaire on a smartphone, you may find it easier to view by rotating the screen.

	None	Less than 1 hour	1 - 3 hours	3 hours +	Prefer not to answer
Physical exercise such as swimming, jogging, aerobics, football, tennis, gym workout etc.	◯	◯	◯	◯	◯
Cycling, including cycling to work and during leisure time	◯	◯	◯	◯	◯
Walking, including walking to work, shopping, for pleasure etc.	◯	◯	◯	◯	◯
Housework/Childcare	◯	◯	◯	◯	◯
Gardening/DIY	◯	◯	◯	◯	◯

How would you describe your usual walking pace?
Please select one option only.

- ◯ Slow pace
- ◯ Steady average pace
- ◯ Brisk pace
- ◯ Fast pace
- ◯ Prefer not to answer

Confidential

Section 7/10: Your health

72% complete
.

Has your lifestyle changed since the beginning of the COVID-19 pandemic?

Select 'This has not changed' if an option does not apply, e.g. you still do not smoke.
If you are answering this questionnaire on a smartphone, you may find it easier to view by rotating the screen.

	I do this more often	This has not changed	I do this less often	Prefer not to answer
Smoking	○	○	○	○
Drinking alcohol	○	○	○	○
Eating healthy food	○	○	○	○
Physical activity (including walking and cycling)	○	○	○	○

Section 7/10: Your health

73% complete
.

Last year, in 2019, how many times did you have a consultation with your GP about your own health?	◯ 0<br>◯ 1<br>◯ 2<br>◯ 3-5<br>◯ 6-10<br>◯ 11-20<br>◯ 21+<br>◯ Prefer not to answer
Last year, in 2019, how many days did you spend as a hospital inpatient?	◯ 0<br>◯ 1<br>◯ 2<br>◯ 3-5<br>◯ 6-10<br>◯ 11-20<br>◯ 21+<br>◯ Prefer not to answer
Did you have a flu vaccine last winter (2019-2020)?	◯ No<br>◯ Yes<br>◯ Do not know<br>◯ Prefer not to answer
Have you had a flu vaccine for this winter (2020-2021)?	◯ No<br>◯ Yes<br>◯ Do not know<br>◯ Prefer not to answer
Have you been contacted by letter or text message to say you are at severe risk from COVID-19 due to an underlying health condition and should be shielding?	◯ No<br>◯ Yes<br>◯ Do not know<br>◯ Prefer not to answer
Do you currently take any of these medications/supplements?<br><br>Please select all that apply. If you do not take any of these, please select "None of these".	☐ Ibuprofen / Nurofen, any other type of non-steroidal anti-inflammatory<br>☐ Vitamin D<br>☐ ACE-inhibitor (e.g. ramipril, lisinopril)<br>☐ Sartan (e.g. losartan, valsartan, candesartan)<br>☐ Entresto (sucubitril/valsartan)<br>☐ Metformin<br>☐ None of these<br>☐ Prefer not to answer

Are you, or do you, currently have any of the following?<br><br>Please select all that apply. If none apply to you, please select "None of the above".	☐ Pregnant<br>☐ Organ transplant<br>☐ Diabetes (Type I or II)<br>☐ Heart disease or heart problems<br>☐ Hypertension<br>☐ Overweight<br>☐ Stroke<br>☐ Kidney disease<br>☐ Liver disease<br>☐ Anaemia<br>☐ Asthma<br>☐ Other lung condition such as COPD, bronchitis or emphysema<br>☐ Cancer<br>☐ Condition affecting the brain and nerves (e.g. Dementia, Parkinson's, Multiple Sclerosis)<br>☐ A weakened immune system or reduced ability to deal with infections (as a result of a disease or treatment)<br>☐ Depression<br>☐ Anxiety<br>☐ Psychiatric disorder<br>☐ None of the above<br>☐ Prefer not to answer

Section 7/10: Your health

74% complete
.

Some of the following questions ask about your mental health. We would like to reassure you that your answers will be treated in accordance with strict research governance procedures, and the study has been reviewed by the Brighton and Sussex Research Ethics Committee.

If do not wish to answer a question, then please click on ‘Prefer not to answer’, or leave it blank.

For help during a mental health crisis or emergency:

Call 999 or go to A&E now if your life is at risk, or if you do not feel you can keep yourself safe.

For free confidential advice or to talk about anything that's troubling you, no matter how difficult:
Call 116 123 to talk to Samaritans at any time of day or night, or email: jo@samaritans.org for a reply within 24 hours Text "SHOUT" to 85258 to contact the Shout Crisis Text Line, or text "YM" if you're under 19 You can find a local NHS urgent mental health helpline here Urgent sources of support are also summarised at this NHS webpage. Other sources of support include: the Covid-19 Workforce Wellbeing pages, the mental health charity Mind, and Victim Support for victims of crime.

74% complete
.

Please select the ONE option that best describes your health TODAY.

MOBILITY

◯ I have no problems in walking about
◯ I have slight problems in walking about
◯ I have moderate problems in walking about
◯ I have severe problems in walking about
◯ I am unable to walk about

© EuroQol Research Foundation. EQ-5D™ is a trade mark of the EuroQol Research Foundation. UK (English) v2.1

74% complete
.

Please select the ONE option that best describes your health TODAY.

◯ I have no problems washing or dressing myself
◯ I have slight problems washing or dressing myself
◯ I have moderate problems washing or dressing myself
◯ I have severe problems washing or dressing myself
◯ I am unable to wash or dress myself

SELF-CARE

© EuroQol Research Foundation. EQ-5D™ is a trade mark of the EuroQol Research Foundation. UK (English) v2.1

74% complete
.

Please select the ONE option that best describes your health TODAY.

USUAL ACTIVITIES (e.g. work, study, housework, family or leisure activities)

◯ I have no problems doing my usual activities
◯ I have slight problems doing my usual activities
◯ I have moderate problems doing my usual activities
◯ I have severe problems doing my usual activities
◯ I am unable to do my usual activities

© EuroQol Research Foundation. EQ-5D™ is a trade mark of the EuroQol Research Foundation. UK (English) v2.1

Confidential

74% complete
.

Please select the ONE option that best describes your health TODAY.

PAIN / DISCOMFORT	◯ I have no pain or discomfort<br>◯ I have slight pain or discomfort<br>◯ I have moderate pain or discomfort<br>◯ I have severe pain or discomfort<br>◯ I have extreme pain or discomfort

© EuroQol Research Foundation. EQ-5D™ is a trade mark of the EuroQol Research Foundation. UK (English) v2.1

74% complete
.

Please select the ONE option that best describes your health TODAY.

ANXIETY / DEPRESSION	◯ I am not anxious or depressed<br>◯ I am slightly anxious or depressed<br>◯ I am moderately anxious or depressed<br>◯ I am severely anxious or depressed<br>◯ I am extremely anxious or depressed

© EuroQol Research Foundation. EQ-5D™ is a trade mark of the EuroQol Research Foundation. UK (English) v2.1

74% complete
.

We would like to know how good or bad your health is TODAY.

This scale is numbered from 0 to 100.
100 means the best health you can imagine.
0 means the worst health you can imagine.

Using the slider, please indicate how your health is TODAY.

0 The worst health you can imagine | 50 | 100 The best health you can imagine

(Place a mark on the scale above)

© EuroQol Research Foundation. EQ-5D™ is a trade mark of the EuroQol Research Foundation. UK (English) v2.1

Section 7/10: Your health

78% complete
.

Over the last 2 weeks, how often have you been bothered by any of the following problems?

If you are answering this questionnaire on a smartphone, you may find it easier to view by rotating the screen.

	Not at all	Several days	More than half the days	Nearly every day	Prefer not to answer
Feeling nervous, anxious or on edge?	◯	◯	◯	◯	◯
Not being able to stop or control worrying?	◯	◯	◯	◯	◯
Little interest or pleasure in doing things	◯	◯	◯	◯	◯
Feeling down, depressed, or hopeless?	◯	◯	◯	◯	◯

How worried are you about your future financial situation?

- ◯ Not at all
- ◯ A little bit
- ◯ Moderately
- ◯ Quite a bit
- ◯ Extremely
- ◯ Prefer not to answer

These questions are about problems and complaints that people sometimes have in response to stressful life experiences. Please indicate how much you have been bothered by each problem in the past month.
If you are answering this questionnaire on a smartphone, you may find it easier to view by rotating the screen.

	Not at all	A little bit	Moderately	Quite a bit	Extremely	Prefer not to answer
Repeated, disturbing memories, thoughts, or images of a stressful experience from the past?	◯	◯	◯	◯	◯	◯
Feeling very upset when something reminded you of a stressful experience from the past?	◯	◯	◯	◯	◯	◯
Avoided activities or situations because they reminded you of a stressful experience from the past?	◯	◯	◯	◯	◯	◯

Woolf K, *et al. BMJ Open* 2021; 11:e050647. doi: 10.1136/bmjopen-2021-050647

Confidential

Section 7/10: Your health

80% complete
.

The following statements describe how people sometimes feel. For each statement, please indicate how often you feel this way.
If you are answering this questionnaire on a smartphone, you may find it easier to view by rotating the screen.

	Hardly ever or never	Some of the time	Often	Prefer not to answer
How often do you feel you lack companionship?	◯	◯	◯	◯
How often do you feel left out?	◯	◯	◯	◯
How often do you feel isolated from others?	◯	◯	◯	◯

Overall, how satisfied are you with your life nowadays?

Please give an answer on a scale of 0 to 10, where 0 is 'not at all' and 10 is 'completely'.

◯ 0 Not at all satisfied
◯ 1
◯ 2
◯ 3
◯ 4
◯ 5
◯ 6
◯ 7
◯ 8
◯ 9
◯ 10 Completely satisfied
◯ Prefer not to answer

Section 7/10: Your health

This is the end of the questions about your mental health. We have repeated the information about sources of support from the beginning of this section here.
For help during a mental health crisis or emergency:

Call 999 or go to A&E now if your life is at risk, or if you do not feel you can keep yourself safe.

For free confidential advice or to talk about anything that's troubling you, no matter how difficult:
Call 116 123 to talk to Samaritans at any time of day or night, or email: jo@samaritans.org for a reply within 24 hours Text "SHOUT" to 85258 to contact the Shout Crisis Text Line, or text "YM" if you're under 19 You can find a local NHS urgent mental health helpline here Urgent sources of support are also summarised at this NHS webpage. Other sources of support include: the Covid-19 Workforce Wellbeing pages, the mental health charity Mind, and Victim Support for victims of crime.

Confidential

Section 8/10: Your health and COVID-19

81% complete
.

This next section asks questions about COVID-19 and your health and your feelings about the pandemic. It will ask about:
What changes you made during the UK national lockdown COVID-19 tests and whether you have had COVID-19 The chances of catching COVID-19 The possible effects of COVID-19 on society Possible vaccines for COVID-19 Attitudes towards the COVID-19 pandemic

Confidential

Section 8/10: Your health and COVID-19

81% complete
.

Have you been in close contact with anyone with COVID-19 outside of your work in the last two weeks?

- ◯ Yes, someone in my household had/has confirmed/tested COVID-19
- ◯ Yes, someone in my household had/has suspected COVID-19
- ◯ Yes, contact with a confirmed/tested COVID-19 case outside of my household
- ◯ Yes, contact with a suspected COVID-19 case outside of my household
- ◯ No, not to my knowledge
- ◯ Prefer not to answer

Thinking back to when COVID-19 was emerging from January 2020 until the beginning of the UK national lockdown on 23rd March 2020, which of the following activities were you doing then, and which have you been doing in the past few weeks?
Please select all that apply.

	Between January 2020and March 2020	Past few weeks	Prefer not to answer
Cancelling my usual social activities	☐	☐	☐
Not going to work	☐	☐	☐
Only going shopping for essential things	☐	☐	☐
Not going to a grocery store or pharmacy	☐	☐	☐
Not leaving the house	☐	☐	☐
Wearing a face mask outside my home	☐	☐	☐
Trying to avoid physical contact with people	☐	☐	☐
Following handwashing recommendations	☐	☐	☐
Using hand sanitiser more than usual	☐	☐	☐
Following coughing and sneezing recommendations	☐	☐	☐
Using tissues more than usual	☐	☐	☐
Wearing gloves while going out of my home	☐	☐	☐
Avoiding public transport	☐	☐	☐
Avoiding going to restaurants/bars/pubs	☐	☐	☐

Avoiding going for walks or exercise outside	☐	☐	☐
Avoiding taking my children out of my home (if applicable)	☐	☐	☐
Prefer not to answer	☐	☐	☐

Thinking back to the months of UK national lockdown which began on 23rd March, which of these is closest to your view?

- ◯ I enjoyed nothing about the UK national lockdown
- ◯ I enjoyed a few aspects of the UK national lockdown
- ◯ I enjoyed some aspects of the UK national lockdown
- ◯ I enjoyed most aspects of the UK national lockdown
- ◯ I enjoyed almost all of the UK national lockdown
- ◯ Prefer not to answer

Confidential

Section 8/10: Your health and COVID-19

83% complete
.

Have you ever had a test to see if you have or have had COVID-19?<br>Select all that apply.	☐ No<br>☐ Yes, A swab test (swab of your throat and/or nose) which tests for active infection<br>☐ Yes, An antibody test for COVID-19 (a blood test, or a drop of blood from your finger) which tests for past infection<br>☐ Do not know<br>☐ Prefer not to answer
What was the reason that you had the swab test?<br>Please select all that apply.	☐ Because I had symptoms<br>☐ Because I have been in contact with someone who had COVID-19<br>☐ Because of my job<br>☐ Before going into hospital as a patient (e.g. for surgery)<br>☐ It was offered by my local council (e.g. posted through my door)<br>☐ For another reason (please specify)<br>☐ Prefer not to answer
Please specify the reason you had the swab test:	______________________
Have you ever had a positive result from a swab test (i.e. showing that you had coronavirus when the swab was taken)?	◯ No<br>◯ Yes<br>◯ Do not know<br>◯ Prefer not to answer
When was the sample taken for the test that came back positive?<br><br>Give the latest date if you have had more than one	______________________
Have you had a positive result from an antibody test (i.e. showing that you had coronavirus at some time previously)?	◯ No<br>◯ Yes<br>◯ Do not know<br>◯ Prefer not to answer
When was the sample taken for the test that came back positive?<br><br>Give the latest date if you have had more than one	______________________

Woolf K, *et al. BMJ Open* 2021; 11:e050647. doi: 10.1136/bmjopen-2021-050647

Have you experienced any of the following symptoms in the past two weeks?<br><br>Please select all that apply.	☐ Fever<br>☐ Cough - dry<br>☐ Cough - mucus or phlegm<br>☐ Sore throat<br>☐ Chest tightness<br>☐ Shortness of breath<br>☐ Runny nose<br>☐ Nasal congestion<br>☐ Sneezing<br>☐ Muscle or body aches<br>☐ Fatigue<br>☐ Unusual loose motions or diarrhoea<br>☐ Vomiting<br>☐ Loss of smell<br>☐ Loss of taste<br>☐ Skin rash<br>☐ None of these<br>☐ Prefer not to answer
Do you think that you currently have or have had COVID-19?	◯ No<br>◯ Unsure<br>◯ Yes, my own suspicions<br>◯ Yes, suspected by a doctor but not tested<br>◯ Yes, confirmed by a positive test<br>◯ Prefer not to answer
When were you told/when did you first think you had COVID-19?	
Month	◯ Jan<br>◯ Feb<br>◯ Mar<br>◯ Apr<br>◯ May<br>◯ Jun<br>◯ Jul<br>◯ Aug<br>◯ Sep<br>◯ Oct<br>◯ Nov<br>◯ Dec<br>◯ Prefer not to answer
Year	◯ 2021<br>◯ 2020<br>◯ 2019<br>◯ Prefer not to answer
Were you hospitalised for treatment of your COVID-19 disease?	◯ No<br>◯ Yes<br>◯ Prefer not to answer
Were you admitted to the intensive care unit (ITU/ICU)?	◯ No<br>◯ Yes<br>◯ Prefer not to answer

How long have you been unwell since having COVID-19?	◯ Less than 3 weeks<br>◯ 3 - 5 weeks<br>◯ More than 5 weeks but less than 3 months<br>◯ More than 3 months but less than 6 months<br>◯ More than 6 months<br>◯ Prefer not to answer
Has any health professional told you that your symptoms are likely to be the ongoing effect of COVID-19?	◯ Yes<br>◯ No<br>◯ Prefer not to answer

Section 8/10: Your health and COVID-19

89% complete
.

How concerned are you that you will get COVID-19 and require hospitalisation?	◯ Not at all concerned<br>◯ A little concerned<br>◯ Quite concerned<br>◯ Very concerned<br>◯ Prefer not to answer
How concerned are you that you might unknowingly spread COVID-19 to others?	◯ Not at all concerned<br>◯ A little concerned<br>◯ Quite concerned<br>◯ Very concerned<br>◯ Prefer not to answer
Do you personally know anyone who has died from COVID-19 (not including patients you have cared for as part of your work)?<br><br>Select all that apply.	☐ Yes, family member(s)<br>☐ Yes, friend(s)<br>☐ Yes, colleague(s)<br>☐ Yes, someone else<br>☐ No<br>☐ Prefer not to answer
Where do you get information about COVID-19?<br><br>Select all that apply.	☐ Friends, family, neighbours<br>☐ Colleagues<br>☐ Employer or manager<br>☐ Television<br>☐ Radio<br>☐ Newspapers or magazines<br>☐ Government or NHS posters, adverts or leaflets<br>☐ Twitter<br>☐ Other social media (e.g. Facebook, Instagram)<br>☐ UK Government website<br>☐ Welsh, Scottish or NI government website<br>☐ NHS website<br>☐ WHO website<br>☐ Other websites<br>☐ Local council<br>☐ My own GP or other healthcare workers<br>☐ Scientific journals<br>☐ Not applicable<br>☐ Prefer not to answer

Woolf K, *et al. BMJ Open* 2021; 11:e050647. doi: 10.1136/bmjopen-2021-050647

Section 8/10: Your health and COVID-19

92% complete
.

What do you think is your personal chance of catching the coronavirus in the next month?

Please enter a value on a scale from 0 to 100, where 0 means there is no possibility that you will and 100 means that you definitely will.

____

What do you think is your personal chance of catching the coronavirus in the next six months?

Please enter a value on a scale from 0 to 100, where 0 means there is no possibility that you will and 100 means that you definitely will.

____

If you do catch coronavirus, what do you think are your chances of needing hospital treatment?
Please enter a value on a scale from 0 to 100, where 0 means there is no possibility that you will and 100 means that you definitely will.

____

What percentage of people in the UK who are hospitalised with coronavirus do you think will end up dying as a result of the disease?
Please enter a value on a scale from 0 to 100, where 0 means nobody hospitalised will die and 100 means everybody hospitalised will die.

____

Section 8/10: Your health and COVID-19

93% complete
.

Have you taken part in a trial of a COVID-19 vaccine?
- ◯ Yes
- ◯ No, but I would if asked
- ◯ No, but I would not if asked
- ◯ Prefer not to answer

If yes, which one?
____

The following question refers to any vaccine you have been offered or may be offered that is not as part of a COVID-19 vaccine trial.

Have you had, or are you going to have, a vaccination against COVID-19?
- ◯ I have already had at least one COVID-19 vaccination
- ◯ I have not had a vaccination but have been told that I will be offered a vaccination in the near future
- ◯ I have been offered a vaccination but have decided not to have the vaccine
- ◯ I have not yet been offered a vaccination but intend to have the vaccine when offered
- ◯ I have not yet been offered a vaccination but have decided not to have a vaccine when offered
- ◯ Prefer not to answer

How many doses have you had?
- ◯ 1
- ◯ 2
- ◯ Unsure
- ◯ Prefer not to answer

Was the vaccination:
- ◯ In a hospital
- ◯ In a care home
- ◯ From a GP
- ◯ Other
- ◯ Prefer not to answer

Please specify:
____

What was the date when you had your first vaccination? (if you are unsure please give your best estimate)
____

Which vaccine did you receive?
- ◯ Pfizer-Biontech
- ◯ Oxford-AstraZeneca
- ◯ Moderna
- ◯ Other
- ◯ Unsure
- ◯ Prefer not to answer

Please specify:
____

Did you consider not having the vaccination?	◯ No<br>◯ Yes, I did consider not having the vaccination<br>◯ Prefer not to answer
What would have been your reason(s) for not having the vaccination? Please select all that apply.	☐ I have allergies, needle-phobia, am immuno-compromised, or have other clinical reasons not to be vaccinated<br>☐ I am concerned about the safety or potential side-effects of a COVID-19 vaccine<br>☐ I am not convinced that COVID-19 vaccines will be effective<br>☐ Vaccines may not have been tested thoroughly in all ethnic groups<br>☐ I have had COVID-19 and therefore do not feel I need the vaccine<br>☐ I am taking part in a clinical trial of a COVID-19 vaccine<br>☐ I would prefer one of the other COVID-19 vaccines that are being developed<br>☐ I would prefer to wait until many other people have received a COVID-19 vaccine<br>☐ I do not feel that I personally am at risk from COVID-19<br>☐ I would rather the vaccine were used for other people who need it more than I do<br>☐ I do not believe in vaccinations in general<br>☐ Other reason<br>☐ Prefer not to answer
Please specify:	__________________________________
When is the vaccination likely to be?	◯ In a few days<br>◯ In the next week<br>◯ In the next two weeks<br>◯ In the next month<br>◯ Other<br>◯ Prefer not to answer
Please specify:	__________________________________
Will this vaccination be:	◯ In a hospital<br>◯ In a care home<br>◯ From a GP<br>◯ Other<br>◯ Prefer not to answer
Please specify:	__________________________________
Are you considering not having the vaccination?	◯ No<br>◯ Yes, I am considering not having the vaccination<br>◯ Prefer not to answer

Woolf K, *et al. BMJ Open* 2021; 11:e050647. doi: 10.1136/bmjopen-2021-050647

Confidential

What might be your reason(s) for not having the vaccination? Please select all that apply.	☐ I have allergies, needle-phobia, am immuno-compromised, or have other clinical reasons not to be vaccinated<br>☐ I am concerned about the safety or potential side-effects of a COVID-19 vaccine<br>☐ I am not convinced that COVID-19 vaccines will be effective<br>☐ Vaccines may not have been tested thoroughly in all ethnic groups<br>☐ I have had COVID-19 and therefore do not feel I need the vaccine<br>☐ I am taking part in a clinical trial of a COVID-19 vaccine<br>☐ I would prefer one of the other COVID-19 vaccines that are being developed<br>☐ I would prefer to wait until many other people have received a COVID-19 vaccine<br>☐ I do not feel that I personally am at risk from COVID-19<br>☐ I would rather the vaccine were used for other people who need it more than I do<br>☐ I do not believe in vaccinations in general<br>☐ Other reason<br>☐ Prefer not to answer
Please specify:	__________________________
Was the vaccination offered by:	◯ A hospital<br>◯ A care home<br>◯ A GP<br>◯ Other<br>◯ Prefer not to answer.
Please specify:	__________________________

Woolf K, *et al. BMJ Open* 2021; 11:e050647. doi: 10.1136/bmjopen-2021-050647

Confidential

What were your reason(s) for not having the vaccination? Please select all that apply.	☐ I have allergies, needle-phobia, am immuno-compromised, or have other clinical reasons not to be vaccinated<br>☐ I am concerned about the safety or potential side-effects of a COVID-19 vaccine<br>☐ I am not convinced that COVID-19 vaccines will be effective<br>☐ Vaccines may not have been tested thoroughly in all ethnic groups<br>☐ I have had COVID-19 and therefore do not feel I need the vaccine<br>☐ I am taking part in a clinical trial of a COVID-19 vaccine<br>☐ I would prefer one of the other COVID-19 vaccines that are being developed<br>☐ I would prefer to wait until many other people have received a COVID-19 vaccine<br>☐ I do not feel that I personally am at risk from COVID-19<br>☐ I would rather the vaccine were used for other people who need it more than I do<br>☐ I do not believe in vaccinations in general<br>☐ Other reason<br>☐ Prefer not to answer
Please specify:	__________________________
When you are offered the vaccine, is there anything that might make you consider not having it?	◯ No<br>◯ Yes, I would consider not having the vaccine<br>◯ Prefer not to answer
What are your reason(s) for considering not having the vaccine? Please select all that apply.	☐ I have allergies, needle-phobia, am immuno-compromised, or have other clinical reasons not to be vaccinated<br>☐ I am concerned about the safety or potential side-effects of a COVID-19 vaccine<br>☐ I am not convinced that COVID-19 vaccines will be effective<br>☐ Vaccines may not have been tested thoroughly in all ethnic groups<br>☐ I have had COVID-19 and therefore do not feel I need the vaccine<br>☐ I am taking part in a clinical trial of a COVID-19 vaccine<br>☐ I would prefer one of the other COVID-19 vaccines that are being developed<br>☐ I would prefer to wait until many other people have received a COVID-19 vaccine<br>☐ I do not feel that I personally am at risk from COVID-19<br>☐ I would rather the vaccine were used for other people who need it more than I do<br>☐ I do not believe in vaccinations in general<br>☐ Other reason<br>☐ Prefer not to answer
Please specify:	__________________________

Woolf K, *et al. BMJ Open* 2021; 11:e050647. doi: 10.1136/bmjopen-2021-050647

What are your reason(s) for not having the vaccination? Please select all that apply.

- ☐ I have allergies, needle-phobia, am immuno-compromised, or have other clinical reasons not to be vaccinated
- ☐ I am concerned about the safety or potential side-effects of a COVID-19 vaccine
- ☐ I am not convinced that COVID-19 vaccines will be effective
- ☐ Vaccines may not have been tested thoroughly in all ethnic groups
- ☐ I have had COVID-19 and therefore do not feel I need the vaccine
- ☐ I am taking part in a clinical trial of a COVID-19 vaccine
- ☐ I would prefer one of the other COVID-19 vaccines that are being developed
- ☐ I would prefer to wait until many other people have received a COVID-19 vaccine
- ☐ I do not feel that I personally am at risk from COVID-19
- ☐ I would rather the vaccine were used for other people who need it more than I do
- ☐ I do not believe in vaccinations in general
- ☐ Other reason
- ☐ Prefer not to answer

Please specify: ______________________________

How much do you agree with the following statements about vaccinations in general?

	1 Strongly disagree	2	3	4	5 Strongly agree	Prefer not to answer
I can rely on vaccines to stop serious infectious diseases	○	○	○	○	○	○
Although most vaccines appear to be safe, there may be problems that we have not yet discovered	○	○	○	○	○	○
Authorities promote vaccination for financial gain, not for people's health	○	○	○	○	○	○
Being exposed to diseases naturally is safer for the immune system than being exposed through vaccination	○	○	○	○	○	○

When, if at all, do you think it will be possible to vaccinate most of the population against coronavirus?

- ○ 1 month from now
- ○ 2 months from now
- ○ 3 months from now
- ○ 6 months from now
- ○ 12 months from now
- ○ 18 months from now
- ○ 2 years from now
- ○ More than 2 years
- ○ Do not know
- ○ Prefer not to answer

Confidential

Section 8/10: Your health and COVID-19

94% complete
.

The coronavirus pandemic will have many effects in the UK, five of which are shown below. How serious do you think each of them is in terms of its consequences?

Please select the relevant number next to the statement (i.e. if you believe "Effects on economy and jobs" to be the most serious then select '1' for this effect).

You will only be able to select each number once.

If you are answering this questionnaire on a smartphone, you may find it easier to view by rotating the screen.

	1 (Most serious)	2	3	4	5 (Least serious)	Prefer not to answer
Effects on children and their education	○	○	○	○	○	○
Effects on the economy and jobs	○	○	○	○	○	○
Increasing deaths as a direct result of catching coronavirus	○	○	○	○	○	○
Increased deaths due to fewer healthcare resources to identify and treat medical conditions other than coronavirus	○	○	○	○	○	○
Increased mental health issues	○	○	○	○	○	○

When a vaccine becomes available, would you wish to receive it?

○ Definitely Yes
○ Probably Yes
○ Probably No
○ Definitely No
○ Prefer not to answer

Section 8/10: Your health and COVID-19

96% complete
.

Do you think the coronavirus crisis will increase or decrease the level of inequality in the UK, compared with before the pandemic?

- ◯ Increase a lot
- ◯ Increase a bit
- ◯ Make no difference
- ◯ Decrease a bit
- ◯ Decrease a lot
- ◯ Prefer not to answer

To what extent do you think the following statements about COVID-19 are true or false? Give your immediate thoughts, and don't look up the answers.

If you are answering this questionnaire on a smartphone, you may find it easier to view by rotating the screen.

	Definitely true	Probably true	Probably false	Definitely false	Prefer not to answer
A person can be infected twice with coronavirus	◯	◯	◯	◯	◯
Coronavirus is less infectious than the influenza virus	◯	◯	◯	◯	◯
Coronavirus was created in a laboratory	◯	◯	◯	◯	◯
Infection with coronavirus is equally likely in men and women	◯	◯	◯	◯	◯
Mortality from coronavirus is higher in men than women	◯	◯	◯	◯	◯
Most people in the UK have already had coronavirus without realising it	◯	◯	◯	◯	◯
The current pandemic is part of a global effort to force everyone to be vaccinated whether they want to or not	◯	◯	◯	◯	◯
The genetic material in a coronavirus is RNA, unlike that of humans which is DNA	◯	◯	◯	◯	◯
The number of people reported as dying from coronavirus is being deliberately reduced or hidden by the authorities	◯	◯	◯	◯	◯
The symptoms that most people blame on coronavirus appear to be linked to 5G network radiation	◯	◯	◯	◯	◯
There is no hard evidence that coronavirus really exists	◯	◯	◯	◯	◯

Confidential

Section 9/10: Your approach to life in general

96% complete
.

People differ in many ways. The questions below cover a range of ways that people can differ from one another, in terms of values, attitudes, and approach to life in general.
For most of the questions there is no right or wrong answer. Do not think too hard about each answer but instead give the one that most immediately seems correct for you, being as honest as you can.

Woolf K, *et al. BMJ Open* 2021; 11:e050647. doi: 10.1136/bmjopen-2021-050647

Confidential

Section 9/10: Your approach to life in general

96% complete
.

The following questions are about how you see yourself as a person.

Please select the number which best describes how you see yourself where 1 means 'does not apply to me at all' and 7 means 'applies to me perfectly'.

I see myself as someone who...

If you are answering this questionnaire on a smartphone, you may find it easier to view by rotating the screen.

	Does not apply to me at all (1)	2	3	4	5	6	Applies to me perfectly (7)	Prefer not to answer
Is sometimes rude to others	○	○	○	○	○	○	○	○
Does a thorough job	○	○	○	○	○	○	○	○
Is talkative	○	○	○	○	○	○	○	○
Worries a lot	○	○	○	○	○	○	○	○
Is original, comes up with new ideas	○	○	○	○	○	○	○	○
Has a forgiving nature	○	○	○	○	○	○	○	○
Tends to be lazy	○	○	○	○	○	○	○	○
Is outgoing, sociable	○	○	○	○	○	○	○	○
Gets nervous easily	○	○	○	○	○	○	○	○
Values artistic, aesthetic experiences	○	○	○	○	○	○	○	○
Is considerate and kind to almost everyone	○	○	○	○	○	○	○	○
Does things efficiently	○	○	○	○	○	○	○	○
Is reserved	○	○	○	○	○	○	○	○
Is relaxed, handles stress well	○	○	○	○	○	○	○	○
Has an active imagination	○	○	○	○	○	○	○	○

Section 9/10: Your approach to life in general

97% complete
.

For each of the following statements, indicate the extent to which you agree or disagree by selecting a number from 1 (Strongly disagree) to 7 (strongly agree).
If you are answering this questionnaire on a smartphone, you may find it easier to view by rotating the screen.

	1 (Strongly disagree)	2	3	4	5	6	7 (Strongly agree)	Prefer not to answer
My life is determined by my own actions	○	○	○	○	○	○	○	○
I am usually able to protect my personal interests	○	○	○	○	○	○	○	○
I can pretty much determine what will happen in my life	○	○	○	○	○	○	○	○
To a great extent, my life is controlled by accidental happenings	○	○	○	○	○	○	○	○
Often there is no chance of protecting my personal interest from bad luck happenings	○	○	○	○	○	○	○	○
When I get what I want, it's usually because I'm lucky	○	○	○	○	○	○	○	○
People like myself have very little chance of protecting our personal interests where they conflict with those of strong pressure groups	○	○	○	○	○	○	○	○
My life is chiefly controlled by powerful others	○	○	○	○	○	○	○	○
I feel like what happens in my life is mostly determined by powerful people	○	○	○	○	○	○	○	○
If someone is meant to have a serious disease, they will get that disease.	○	○	○	○	○	○	○	○
My health is determined by fate.	○	○	○	○	○	○	○	○
My health is determined by something greater than myself.	○	○	○	○	○	○	○	○
I will stay healthy if I am lucky.	○	○	○	○	○	○	○	○
I like having a clear and structured mode of life	○	○	○	○	○	○	○	○

I would quickly become impatient and irritated if I could not find a solution to a problem immediately	○	○	○	○	○	○	○	○
Genes are more important than one's own behaviour in determining one's health	○	○	○	○	○	○	○	○

Woolf K, *et al. BMJ Open* 2021; 11:e050647. doi: 10.1136/bmjopen-2021-050647

Confidential

Section 9/10: Your approach to life in general

98% complete
.

How do you see yourself: are you generally a person who is fully prepared to take risks or do you try to avoid taking risks?

Please select a number on the scale, where the value 0 means: 'not at all willing to take risks' and the value 10 means: 'very willing to take risks'.

- ◯ 0 Not at all willing to take risks
- ◯ 1
- ◯ 2
- ◯ 3
- ◯ 4
- ◯ 5
- ◯ 6
- ◯ 7
- ◯ 8
- ◯ 9
- ◯ 10 Very willing to take risks
- ◯ Prefer not to answer

Think back to the work you were doing at the end of 2019 or in early 2020 before the UK national lockdown on 23rd March 2020. How well do the following statements describe your typical work environment at that time?

If you had more than one job, think about your main job/role. If you were not working please select “not applicable”.

If you are answering this questionnaire on a smartphone, you may find it easier to view by rotating the screen.

	Definitely disagree	Somewhat disagree	Somewhat agree	Definitely agree	Not applicable	Prefer not to answer
There was a real opportunity for me to choose the particular things I worked on	◯	◯	◯	◯	◯	◯
My work colleagues really tried hard to get to know one another	◯	◯	◯	◯	◯	◯
I had a lot of choice about the work I did	◯	◯	◯	◯	◯	◯
I was required to do too many different things	◯	◯	◯	◯	◯	◯
My coworkers were supportive and friendly towards me	◯	◯	◯	◯	◯	◯
There seemed to be too much work to get through	◯	◯	◯	◯	◯	◯

Think back to the work you were doing at the end of 2019 or early 2020 before the UK national lockdown on 23rd March 2020. How well do the following statements describe overall the way you felt about your work at that time?

If you had more than one job, think about your main job/role. If you were not working please select “Not applicable”.

If you are answering this questionnaire on a smartphone, you may find it easier to view by rotating the screen.

Confidential

	Never	A few times a year	Once a month or less	A few times a month	Once a week	A few times a week	Every day	Not applicable	Prefer not to answer
I was exhausted in the morning at the thought of another day at work	○	○	○	○	○	○	○	○	○
When I was working I forgot everything else around me	○	○	○	○	○	○	○	○	○
I didn't have enough energy for family and friends during leisure time	○	○	○	○	○	○	○	○	○
I was proud of the work that I did	○	○	○	○	○	○	○	○	○
At work I felt bursting with energy	○	○	○	○	○	○	○	○	○

Section 10/10: A few last questions

99% complete
.

The questionnaire is now almost finished, and we are very grateful to you for having completed it so far and having contributed to the UK-REACH study.
People filling in questionnaires often feel that although they have answered a lot of questions they often have not had the chance to say what they really feel about issues that have come up. There are therefore three open-ended questions on very broad issues, to do with:

- Why ethnic minorities might be more vulnerable to COVID-19
- How society might change as a result of COVID-19
- How your own future might change as a result of COVID-19

Only if you want to, please type whatever you want to say into the three boxes. You should have more than enough space, and the computer will tell you how much space you have left. Often open-ended comments such as these can provide much of interest to researchers.
Finally, there are two short questions asking for a little feedback on the questionnaire itself, about whether you think it might be useful and whether you think it was far too long.

What are your thoughts on why people from ethnic minorities working in health and care have been more severely affected by COVID-19? ________________________________

How do you see society changing as a result of COVID-19? ________________________________

How do you see your own future changing as a result of COVID-19? ________________________________

Two very last questions:

We know that the questionnaire was long. Did you think:
- ◯ It should have asked about a lot more things
- ◯ It should have asked about some more things
- ◯ It was about the right length
- ◯ It should have been shorter
- ◯ It should have been much shorter

How useful do you think this questionnaire might be for researching and understanding COVID-19 in ethnic minorities?
- ◯ Not at all useful
- ◯ Not very useful
- ◯ Fairly useful
- ◯ Very useful
- ◯ Extremely useful

Thank you very much for your assistance in this study, which is very much appreciated.

Information about the progress of the study and the research findings will be placed on the UK-REACH website.
If you are affected by any of the issues raised in this questionnaire or are looking for information on COVID-19 (coronavirus) please visit:
• Government guidelines: www.gov.uk/coronavirus
• NHS advice: https://www.nhs.uk/conditions/coronavirus-covid-19/symptoms/
• NHS – Where to get urgent help for mental health: https://www.nhs.uk/using-the-nhs/nhs-services/mental-health-services/where-to-get-urgent-help-for-mental-health/
• Covid-19 Workforce Wellbeing: https://www.practitionerhealth.nhs.uk/covid-19-workforce-wellbeing
• Victim Support – support for people affected by crime or traumatic events, including hate crime: https://www.victimsupport.org.uk/
• Samaritans – Emotional support for everyone: www.samaritans.org
• Mind – Advice and support for anyone with a mental health problem: www.mind.org.uk
