# Supplementary material for: The United Kingdom Research study into Ethnicity And COVID-19 outcomes in Healthcare workers (UK-REACH): protocol for a prospective longitudinal cohort study of healthcare and ancillary workers in UK healthcare settings
Source: BMJ Open. 2021 Sep 17;11(9):e050647. doi: 10.1136/bmjopen-2021-050647 (PMC8450967; doi:10.1136/bmjopen-2021-050647)
Supplement: Author's manuscript [file bmjopen-2021-050647.draft_revisions.pdf]

BMJ Open is committed to open peer review. As part of this commitment we make the peer review history of every article we publish publicly available.

When an article is published we post the peer reviewers' comments and the authors' responses online. We also post the versions of the paper that were used during peer review. These are the versions that the peer review comments apply to.

The versions of the paper that follow are the versions that were submitted during the peer review process. They are not the versions of record or the final published versions. They should not be cited or distributed as the published version of this manuscript.

BMJ Open is an open access journal and the full, final, typeset and author-corrected version of record of the manuscript is available on our site with no access controls, subscription charges or pay-per-view fees (http://bmjopen.bmj.com).

If you have any questions on BMJ Open's open peer review process please email info.bmjopen@bmj.com

BMJ Open

The United Kingdom Research study into Ethnicity And COVID-19 outcomes in Healthcare workers (UK-REACH): Protocol for a prospective longitudinal cohort study of healthcare and ancillary workers in UK healthcare settings

Journal:	*BMJ Open*
Manuscript ID	bmjopen-2021-050647
Article Type:	Protocol
Date Submitted by the Author:	01-Mar-2021

Complete List of Authors:	Woolf, Katherine; University College London, Research Department of Medical Education<br>Melbourne, Carl; University of Leicester, Genetic Epidemiology Research Group, Department of Health Sciences<br>Bryant, Luke; University of Leicester, Department of Respiratory Sciences<br>Guyatt, Anna; University of Leicester, Genetic Epidemiology Research Group, Department of Health Sciences<br>McManus, Ian; University College London, Research Department of Medical Education<br>Gupta, Amit; Oxford University Hospitals NHS Foundation Trust<br>Free, Robert; University of Leicester, Department of Respiratory Sciences; NIHR Leicester Biomedical Research Centre Respiratory Diseases<br>Nellums, Laura ; University of Nottingham, Division of Epidemiology and Public Health<br>Carr, Sue; University Hospitals of Leicester NHS Trust; General Medical Council<br>John, Catherine; University of Leicester, Department of Health Sciences<br>Martin, Christopher; University of Leicester, Department of Respiratory Sciences; University Hospitals of Leicester NHS Trust, Department of Infection and HIV Medicine<br>Wain, Louise; University of Leicester, Department of Health Sciences; NIHR Leicester Biomedical Research Centre Respiratory Diseases<br>Gray, Laura; University of Leicester, Department of Health Sciences<br>Garwood, Claire; University of Leicester, Department of Respiratory Sciences<br>Modhwadia, Vishant; University of Leicester, Department of Respiratory Sciences<br>Abrams, Keith; University of York, Centre for Health Economics<br>Tobin, Martin; University of Leicester, Genetic Epidemiology Research Group, Department of Health Sciences; NIHR Leicester Biomedical Research Centre Respiratory Diseases, Glenfield Hospital<br>Khunti, Kamlesh; University of Leicester, Diabetes Research Centre<br>Pareek, Manish; University of Leicester, Department of Respiratory Sciences; University Hospitals of Leicester NHS Trust, Department of Infection and HIV Medicine

Keywords:	COVID-19, Public health < INFECTIOUS DISEASES, MENTAL HEALTH

I, the Submitting Author has the right to grant and does grant on behalf of all authors of the Work (as defined in the below author licence), an exclusive licence and/or a non-exclusive licence for contributions from authors who are: i) UK Crown employees; ii) where BMJ has agreed a CC-BY licence shall apply, and/or iii) in accordance with the terms applicable for US Federal Government officers or employees acting as part of their official duties; on a worldwide, perpetual, irrevocable, royalty-free basis to BMJ Publishing Group Ltd ("BMJ") its licensees and where the relevant Journal is co-owned by BMJ to the co-owners of the Journal, to publish the Work in this journal and any other BMJ products and to exploit all rights, as set out in our licence.

The Submitting Author accepts and understands that any supply made under these terms is made by BMJ to the Submitting Author unless you are acting as an employee on behalf of your employer or a postgraduate student of an affiliated institution which is paying any applicable article publishing charge ("APC") for Open Access articles. Where the Submitting Author wishes to make the Work available on an Open Access basis (and intends to pay the relevant APC), the terms of reuse of such Open Access shall be governed by a Creative Commons licence – details of these licences and which Creative Commons licence will apply to this Work are set out in our licence referred to above.

Other than as permitted in any relevant BMJ Author's Self Archiving Policies, I confirm this Work has not been accepted for publication elsewhere, is not being considered for publication elsewhere and does not duplicate material already published. I confirm all authors consent to publication of this Work and authorise the granting of this licence.

For peer review only

The United Kingdom Research study into Ethnicity And COVID-19 outcomes in Healthcare workers (UK-REACH): Protocol for a prospective longitudinal cohort study of healthcare and ancillary workers in UK healthcare settings

Katherine Woolf*[1], Carl Melbourne*[2], Luke Bryant*[3], Anna L Guyatt[2], Chris McManus[1], Amit Gupta[4], Robert C Free[3,9], Laura Nellums[5], Sue Carr[6,7], Catherine John[2], Christopher A Martin[3,8], Louise V Wain[2,9], Laura J Gray[2], Claire Garwood[3], Vishant Modhwadia[3], Keith Abrams[10], Martin D Tobin[2,9], Kamlesh Khunti[11], Manish Pareek[3,8]** on behalf of the UK-REACH Study Collaborative Group+

*Joint first authors; **Corresponding author manish.pareek@leicester.ac.uk

Affiliations

1 Research Department of Medical Education, University College London Medical School, Gower Street, London WC1E 6BT, UK.

2 Department of Health Sciences, University of Leicester, University Road, Leicester, LE1 7RH, UK.

3 Department of Respiratory Sciences, University of Leicester, Leicester, LE1 7RH, UK.

4 Oxford University Hospitals NHS Foundation Trust, Headley Way, Headington, Oxford OX3 9DU, UK.

5 Division of Epidemiology and Public Health, School of Medicine, University of Nottingham, City Hospital Campus, Hucknall Road, NG5 1PB, UK.

6 University Hospitals Leicester NHS Trust, Level 3, Balmoral Building, Leicester Royal Infirmary, Infirmary Square, Leicester, LE1 5WW, UK.

7 General Medical Council, 3BH, 350 Euston Rd, London NW1 3JN, UK.

8 Department of Infection and HIV Medicine, University Hospitals of Leicester NHS Trust, Leicester, LE1 5WW, UK.

9 National Institute for Health Research Leicester Biomedical Research Centre - Respiratory, Glenfield Hospital, Leicester, LE3 9QP UK.

10 Centre for Health Economics, University of York, York, UK.

11 Diabetes Research Centre, University of Leicester, Leicester LE5 4PW, UK

UK-REACH Study Collaborative Group (*Work Package 2 collaborators):

Manish Pareek (Chief investigator)*, Amani Al-Oraibi, Amit Gupta**, Anna Guyatt, Carl Melbourne*, Catherine John*, Christopher A Martin*, I Chris McManus*, Chris Orton, Claire Garwood*, David Ford, Edward Dove, Fatimah Wobi, Janet Hood, Kamlesh Khunti*, Katherine Woolf*, Keith Abrams*,

For peer review only - http://bmjopen.bmj.com/site/about/guidelines.xhtml

Laura J Gray*, Laura Nellums*, Louise V Wain*, Lucy Teece, Luke Bryant*, Martin Tobin,* Mayuri Gogoi, Osama Hassan, Robert C Free*, Ruby Reed-Berendt, Sue Carr*, Vishant Modhwadia*.

Abstract

Introduction

The COVID-19 pandemic has resulted in significant morbidity and mortality, and devastated economies globally. Among groups at increased risk are healthcare workers (HCWs) and ethnic minority groups. Emerging evidence suggests HCWs from ethnic minority groups are at increased risk of adverse COVID-19-related outcomes. To date there has been no large-scale analysis of these risks in UK HCWs or ancillary workers in healthcare settings, stratified by ethnicity or occupation, and adjusted for confounders. This paper reports the protocol for a prospective longitudinal questionnaire study of UK HCWs, as part of the UK-REACH programme (The United Kingdom Research study into Ethnicity And COVID-19 outcomes in Healthcare workers).

Methods and analysis

A baseline questionnaire with follow-ups at 4 and 8 months will be administered to a national cohort of UK HCWs and ancillary workers in healthcare settings, and those registered with UK healthcare regulators. With consent, data will be linked to health records with 25 year follow-up.

Univariate associations between ethnicity and clinical COVID-19 outcomes, physical and mental health, and key confounders/explanatory variables will be tested. Multivariable analyses will test for associations between ethnicity and key outcomes adjusted for the confounder/explanatory variables. Multilevel models will model changes over time by ethnic group, facilitating understanding of absolute and relative risks in different ethnic groups, and generalisability of findings.

Ethics and dissemination.

The study is approved by Health Research Authority (reference 20/HRA/4718), and carries minimal risk. We aim to manage the small risk of participant distress about questions on sensitive topics by clearly participant information that the questionnaire covers sensitive topics and there is no obligation to answer these or any other questions, and by providing support organisation links. Results will be disseminated with reports to Government and papers submitted to pre-print servers and peer reviewed journals.

Registration details.

Trial ID: ISRCTN11811602

For peer review only - http://bmjopen.bmj.com/site/about/guidelines.xhtml

STRENGTHS AND LIMITATIONS OF THIS STUDY

- National, UK-wide, study, aiming to capture variety of healthcare worker job roles including ancillary workers in healthcare settings.
- Longitudinal study including three waves of questionnaire data collection, and linkage to administrative data over 25 years, with consent.
- Unique support from all major UK healthcare worker regulators, relevant healthcare worker organisations, and a Professional Expert Panel to increase participant uptake and the validity of findings.
- Potential for self-selection bias and low response rates, and the use of electronic invitations and online data collection makes it harder to reach ancillary workers without regular access to work email addresses.

For peer review only

For peer review only - http://bmjopen.bmj.com/site/about/guidelines.xhtml

INTRODUCTION

COVID-19 has spread rapidly across the world, causing significant morbidity and mortality, and devastating health economies in many countries. Healthcare workers (HCWs) have been identified as being at increased risk of SARS-CoV-2 infection and adverse outcomes,(1-3) as have individuals from ethnic minority groups.(2, 4-13) Emerging evidence suggests that ethnic minority groups may also be at greater risk of long-term COVID-19 sequelae.(4) HCWs and individuals from ethnic minority groups may also be at increased risk of COVID-19-related poor mental health outcomes, including anxiety, depression and post-traumatic stress.(14-19)

There are concerns that HCWs from ethnic minority groups are at particular risk of SARS-CoV-2 infection and adverse COVID-19 outcomes compared to white HCWs (3, 20, 21) However, the quality of data relating to COVID-19 outcomes in HCWs remains poor, with no large representative studies of the risk of COVID-19 infection or adverse outcomes in healthcare workers or ancillary workers in healthcare settings (hereafter "HCWs") stratified by ethnicity or occupation type, controlling for potential confounders.

To address this, UK-REACH (United Kingdom Research study into COVID-19 outcomes in Healthcare workers) will rapidly examine differences in COVID-19 diagnosis, clinical outcomes (diagnosis, hospitalisation, ICU admission), professional roles, and well-being among ethnic minority and white HCWs through a unique large database analysis (rapid linkage and analysis of anonymised professional registration and National Health Service (NHS) datasets), longitudinal cohort study, legal/ethical analysis, and qualitative work packages. This work will provide information on very short-term outcomes and produce rapid actionable outputs, as well as enabling investigations of the medium/long-term effects of COVID-19 on HCWs in future studies through the linkage and cohort study. This protocol describes the UK-REACH longitudinal cohort study.

Research Question

What is the impact of COVID-19 on the physical and mental health of ethnic minority HCWs compared to white HCWs in the short term and the longer term?

Aims

To examine the relationship between ethnicity and COVID-19-related mental and physical health outcomes through the establishment of a novel longitudinal cohort study of HCWs, including recruitment from the membership bodies and professional registers for different healthcare roles, providers of facilities management, and directly from UK healthcare settings.

To study changes in health outcomes, social circumstances and professional roles of HCWs of different ethnicities, over the course of the COVID-19 pandemic and to characterise longer-term outcomes and consequences.

To measure differences in the impact of COVID-19 infection and working during the pandemic on physical and mental health in a multi-ethnic group of HCWs in the UK.

Objectives

To survey HCWs at baseline to collect data on demographics, job role, attitudes to work and work climate, social and living circumstances, values and personality, and physical and mental health.

To collect baseline biological samples for future analysis in a subsample of consenting participants.

For peer review only - http://bmjopen.bmj.com/site/about/guidelines.xhtml

To conduct follow-up surveys and samples over 12 months in order to capture changes over subsequent COVID-19 pandemic waves.

To link survey data to participant healthcare records, with consent.

METHODS AND ANALYSIS

Study design

National prospective longitudinal cohort study in all four nations of the United Kingdom.

Setting

Healthcare workers and ancillary workers in healthcare settings within the UK.

Participants

Inclusion criteria

Age ≥ 16 years;

Living in the UK;

Healthcare worker or ancillary worker in a UK healthcare setting OR Registered with the following UK healthcare professional regulatory bodies: the General Medical Council (GMC), Nursing and Midwifery Council (NMC), General Dental Council (GDC), Health and Care Professions Council (HCPC), General Optical Council (GOC), General Pharmaceutical Council (GPC) or the Pharmaceutical Society of Northern Ireland (PSNI);

Willing and able to give informed consent.

Exclusion criteria

Age <16 years;

Living outside the UK;

Not a healthcare worker or ancillary worker in a healthcare setting AND not regulated by one of the professional regulatory bodies listed above;

Unwilling and/or unable to give informed consent.

Sample size

We aim to recruit at least 32,000 HCWs (66% from ethnic minority groups). See Figure 1 for the study flow chart. The proposed sample will approximately comprise:

- 10,000 doctors
- 10,000 nurses, midwives and nursing associates
- 4,000 ancillary workers
- 2,000 allied health professionals
- 2,000 ambulance workers
- 2,000 pharmacists and pharmacy technicians
- 1,000 dentists and dental care professionals
- 1,000 optometrists and dispensing opticians

For peer review only - http://bmjopen.bmj.com/site/about/guidelines.xhtml

For peer review only

Whilst the above numbers represent our target numbers for recruitment, we will welcome participants working in other roles within healthcare settings. We may adapt target sample sizes for subgroups following initial response, to ensure we have appropriate representation of ethnic minority participants in each job role.

This sample size has been pragmatically chosen to allow for adequate representation of all ethnic groups within all job-role categories. Based on general population prevalence estimates (11) producing conservative power estimates, we anticipate at least 80% (statistical) power to detect modest effects of ethnicity (RR ≥1.5) for more prevalent outcomes (e.g. COVID-19 diagnosis) and larger effects (RR ≥2) for rarer outcomes (e.g. mortality). Power calculations will be reviewed to reflect changing rates of infection.

Recruitment

Recruitment will be via several routes, and will be incentivised by the inclusion of a prize draw for those who complete the questionnaire:

i) Email, letter, and/or text message invitations from healthcare professional regulators.

The records and registrations with the GMC, NMC, GDC, HCPC, GOC, GPC, PSNI will be used as a sampling frame. Where possible, we will utilise demographic data routinely collected by regulators to oversample for people identifying as being from ethnic minority backgrounds. We will endeavour to sample representatively across age groups, sex/gender, job roles, and other characteristics, in order to maximise the generalisability of our findings. Regulators will send invitations and reminders on behalf of the principal investigator. Alongside these invites regulators will also promote the study through their social media channels as the invites go out.

ii) Targeted advertisement to key staff groups through healthcare organisations.

We will advertise the study through the general communication channels of regulators, professional bodies (e.g. Royal College of Midwives, Royal College of General Practitioners), Health Education bodies, and other relevant organisations (e.g. the British Medical Association, the British Association of Physicians of Indian Origin (BAPIO), The Filipino Nurses Association United Kingdom). This will include promotion through newsletters, web pages, email communications and banners on self-service portals (for example, on the NHS Electronic Staff Record portal used for accessing payslips).

iii) General publicity of the study via print and broadcast media, social media for the study and other relevant organisations, and posters or flyers in workplaces, as relevant to participant staff groups.

iv) Direct invitation and recruitment via UK healthcare providers.

UK healthcare providers, including at least 30 NHS Hospital Trusts, will advertise to potential participants by email, text, mail, verbally, or through posters/flyers. Trusts will be selected to represent a range of geographical areas (to include England, Scotland, Wales, and Northern Ireland). We will aim to recruit from both large teaching hospitals and smaller community healthcare trusts, and will take into consideration regional ethnic diversity when selecting trusts. We will also utilise a study infographic to promote recruitment through NHS trusts. Recruitment of ancillary staff has been facilitated by Serco at specific trusts, through utilisation of content handouts and posters which have been cascaded by contractors on site.

For peer review only - http://bmjopen.bmj.com/site/about/guidelines.xhtml

Invitations and advertisements will direct staff via a weblink and/or QR code to the study recruitment site. In the case of direct recruitment through UK healthcare settings, potential participants will be supported to join with the help of suitably trained members of the local research team where appropriate. Reminders will also be sent to improve recruitment to the study.

Data collection

See Figure 2 for the study timeline.

To gain informed consent, potentially interested participants will have the opportunity to read the UK-REACH participant information sheet (PIS) online via a web application, or in person with a member of the local research team, before deciding if they would like to take part. Potential participants will be asked to register for a UK-REACH account (https://uk-reach.org/) by entering an email address and password, then will be asked to sign an online consent form. To complete registration, participants will be asked to provide personal details including name, date of birth, and address. Participants will be considered enrolled into the study after completing the registration process, and giving their electronic consent on a secure web page. They will be assigned a unique identifier at this point. This will be securely retained in an electronic database at the University of Leicester, which will also function as an enrolment log.

Once translation has been undertaken and checked, we will make study materials (PIS and consent form) available in alternative languages commonly used by workers in healthcare settings as required. These will be made available on the UK-REACH website.

After consenting to participation in the study, participants will be asked to complete a baseline online questionnaire on demographics, job role and other work circumstances, location of work/residence, interaction with COVID-19 patients (including access to personal protective equipment , social and living circumstances, discrimination and harassment, values and personality, and physical and mental health (see Measures, below). This questionnaire will be accessible via the study website, and support to complete the questionnaire will also be available from the study team. Some identifiable data (e.g. name, DOB, address) will be collected during registration and/or the questionnaire to facilitate secure data linkage (see Data linkage, below).

Participants will be invited to complete the baseline questionnaire between December 2020 and January 2021. Participants will have the option of completing two further questionnaires, one at approximately 4 months from baseline and one at approximately 8 months from baseline. Consent will be requested to follow up participants for up to 25 years (subject to funding), and during this time serial questionnaire data will be collected, and periodic linkage with healthcare records will take place (see Data linkage, below).

Data linkage

Participants will be asked to provide consent for the study to follow their health by extracting information from their past and future NHS health care records (including NHS number), any COVID-19 related records, and from "COVID-19 symptom study" websites or apps (22) if they use them.

Initially, questionnaire and personal data, for which consent has been given, will be electronically transferred to University of Leicester, and stored separately on secure servers. A study ID for each participant together with identifiable data will be sent to NHS Wales Informatics Service in order to acquire the list of NHS numbers/Community Health Index (CHI) number (for Scotland)/Health and

For peer review only - http://bmjopen.bmj.com/site/about/guidelines.xhtml

Care number (for Northern Ireland) in partnership with the relevant UK NHS data providers. Study ID and NHS/CHI/H&C number will then be used to link to healthcare records by the relevant data provider across the UK. De-identified records will be sent directly to the Secure Anonymised Information Linkage databank (SAIL, https://saildatabank.com), retaining Study ID. Separately, study ID and corresponding questionnaire data will be sent from the University of Leicester to SAIL. These datasets will be linked within SAIL to provide the complete dataset. Interim analysis of unlinked questionnaire data will be performed at the University of Leicester using pseudonymised datasets. All linked data analysis will take place within SAIL.

Questionnaire design

The questionnaires will be designed by the study team with input from the Professional Expert Panel (PEP) - see Patient and Public Involvement, below. Where possible, previously used and/or validated measures are being used. The study team will devise measures where none are available.

The baseline questionnaire will ask about participants' current experiences and attitudes, as well as collecting some retrospective data about participants' experiences and attitudes at the start of the pandemic and/or pre-pandemic. Follow-up questionnaires will repeat outcome measures, and may include new items relevant to the progression of the pandemic. The data dictionary (https://www.uk-reach.org/data-dictionary) contains the source(s) for each question. The baseline questionnaire is included as an Appendix.

Patient and public involvement

The UK-REACH team have worked closely with national and local organisations representing ethnic minority HCWs, who have been closely involved in identifying the research questions, deciding the study methodology, and are included either as members of the study delivery team or collaborators. They are also part of the Stakeholder group (see Supplementary Information for list of organisations) that will meet monthly to monitor study progress and research outputs, and provide advice to the research team on study delivery and progress. This group will also be central in disseminating the research findings. Alongside the high-level national organisation input into this stakeholder group, we will have representation from ethnic minority HCWs, including those who have contracted COVID-19 (mild to severe), to provide feedback on our work and how it should be disseminated. Members of our stakeholder/public engagement group will also sit on the Scientific Committee to ensure there is a seamless flow of information from the research team and the public engagement/stakeholder group. In addition we are working closely with the Centre for BME Health (Leicester, UK) to ensure that we are working to engage staff from a range of ethnic groups.

A Professional Expert Panel (PEP) will provide feedback on UK-REACH study materials and sampling methodology such as surveys, questionnaires, and interview and focus group topic guides. The PEP is made up of individuals who work within a healthcare setting from a range of ethnic backgrounds, occupational backgrounds, and genders. Staff have unique insight related to their professions or ethnic groups and are, therefore, in a position to provide advice and lived experience related to certain aspects of the project. The aim is for the PEP to draw on their experiences when providing their comments to ensure research instruments are at optimum suitability for study participants. The PEP meets virtually on a bi-monthly basis via Microsoft Teams. Study items/documents for discussion are circulated a week in advance of the PEP meeting and the group's Chair and Co-chair (PEP members who both volunteered to take on the role) moderate the meeting. UK-REACH team members are present in order to answer study-specific queries, and so only enter the discussions to

For peer review only - http://bmjopen.bmj.com/site/about/guidelines.xhtml

do so. The PEP also interacts with the study team between meetings via email with any additional feedback.

Primary outcome measures

Clinical COVID-19 outcomes

Participants will be asked to self-report COVID-19 infection, defined as either a positive SARS-CoV-2 polymerase chain reaction (PCR) or antibody test, or as self-reported suspected infection; the latter will be particularly relevant for those reporting historic illness early in the pandemic before widespread availability of testing.

Those reporting COVID-19 illness will be asked about: date of onset, the nature of symptoms experienced and their duration, and hospitalisation (including any time spent in intensive care). Corroboration of the details of these outcomes will be possible using linked electronic healthcare records (see "Measures obtained via data linkage", below).

General health

This will be measured using the EQ-5D-5L instrument(23) (https://euroqol.org/), which contains five dimensions on mobility, self-care, usual activities, pain and discomfort, and anxiety and depression, plus an overall self-report of health.

Mental health

This will be measured using the Patient Health Questionnaire-2 (PHQ-2)(24) for depression, the Generalised Anxiety Disorder-2 (GAD-2)(25) scale for anxiety, a three-item abbreviated version of the PCL-6 scale for post-traumatic stress disorder,(26) a three-item abbreviated version of the UCLA Loneliness scale, (27) and an Office for National Statistics question about overall life satisfaction.(28) Participants will also be asked key questions from the Utrecht Work Engagement Scale (29) and the GMC National Training Survey questions on burnout(30) (from the Copenhagen Burnout Inventory (31)).

Questionnaire measures

Ethnicity

In this study we will ask participants to self-identify the ethnic group with which they most identify using the 18 UK Census 2011 Categories.(32) The questionnaire also asks the ethnic group of any partner and of parents.

We will then collapse these 18 categories into five main ethnic categories also defined within the Census (Black, Asian, Mixed, Other, White). We will further collapse them into two groups which we will refer to as "white" (White British, White Irish, White Gypsy or Traveller, White Other) and "ethnic minority" (all other ethnic groups). There is currently considerable debate about the categorisation of people using ethnic groups, and in particular, the grouping of people who do not identify as white into a single category. There is also considerable debate and controversy about the words used to describe such a broad and heterogeneous grouping, with terms such as "people of colour", "Black Asian and Minority Ethnic" or "BAME" used. In our choice of terms we have followed the BMJ who in their special edition on Racism in Medicine use the term "ethnic minority" as one that is most likely to be understood by our study population.(33) We fully acknowledge that broad ethnic groupings can mask important ethnic and cultural differences, and where possible we will use more refined ethnic groupings, while also acknowledging the heterogeneity within them.

For peer review only - http://bmjopen.bmj.com/site/about/guidelines.xhtml

Nationality, religion, and languages

Country of birth, nationality, parents' country of birth, grandparents' country of birth (born in UK / not born in UK), age learned English (if second language), language(s) spoken at home (currently and as a child), religion, religiosity, ethnic identity.

Other demographics and education

Age, gender, sex, marital status, highest level of education completed, year and country of primary professional qualification (if applicable), highest level of education achieved by parents.

Work

Job role(s), sector(s), grade and specialty (for doctors), NHS band (for other HCWs), registered field of practice (nurses); work location(s); whether currently working, reasons for not working (if applicable); hours worked in a typical week; frequency of night shifts; contact with patients (with and without COVID-19), colleagues and others (remotely, face-to-face with social distancing, with physical contact); time spent travelling to and from work, modes of transport; access to, use of, and training to use personal protective equipment (PPE); exposure to aerosol-generating procedures; NHS COVID-19 risk assessment experiences; feelings about raising a clinical concern at work, perceptions of fairness of organisation with regards career progression; redeployment as a result of the pandemic, patient exposure, training and supervision in redeployed role (if applicable); proportion of colleagues of same ethnicity to self, proportion of colleagues who are white; work engagement.

Home and social life

Household composition (numbers, ages, relationship to participant) and sharing of accommodation; number of household members travelling using public transport or in jobs that bring them into close contact with others; childcare and support "bubbles"; length of time at current address; type and size of accommodation including amount of shared space and access to safe outdoor space; numbers of social contacts (remotely, face to face with social distancing, with physical contact), proportion of friends of the same ethnic group to self.

Harassment and discrimination

Experiences of discrimination in day-to-day life; discrimination at work and whether made a complaint (if applicable).

Physical health, mental health, and wellbeing

Height, weight, smoking and alcohol use, physical activity at work, general physical activity, change in lifestyle since start of pandemic, healthcare experience in 2019 (GP consultations and hospital admittance), flu vaccine uptake, medication, health conditions and pregnancy, quality of life, general anxiety, depression, post-traumatic stress disorder (PTSD), loneliness, and general life satisfaction.

COVID-19 experiences and beliefs

COVID-19 exposure, testing and test positivity; symptoms experienced, plus their severity and longevity, including diagnosis of long-COVID (if applicable); behaviour changes due to COVID-19; concern, knowledge and beliefs about COVID-19; COVID-19 information sources; enjoyment of first national lockdown (Spring 2020), COVID-19 vaccine trial participation; COVID-19 vaccination intention including offers, uptake (including vaccine brand), or reasons for refusal, and vaccine beliefs.

For peer review only - http://bmjopen.bmj.com/site/about/guidelines.xhtml

Trait and state psychological measures

'Big five' personality traits, locus of control, health locus of control, risk taking, burnout, personal need for structure.

Open-ended questions

The baseline questionnaire will include three open-ended free-text questions: "What are your thoughts on why people from ethnic minorities working in health and care have been more severely affected by COVID-19?", "How do you see society changing as a result of COVID-19?", "How do you see your own future changing as a result of COVID-19?".

Evaluation questions

Views on the length of the questionnaire and on its usefulness for understanding ethnicity and COVID-19.

Measures obtained via data linkage

Data linkage will be used to corroborate COVID-19 clinical outcomes (acute infection, antibody positivity), major comorbidities and patterns of healthcare use.

Biological sampling

At baseline we will also seek consent to re-contact participants in the future for DNA sampling and sampling related to immune profiling although we will submit an amendment to implement this sampling and detail the specifics relating to this at the time of submitting the amendment.

Statistical analysis

Descriptive statistics will be calculated for the primary outcome measures, and for ethnicity and key confounder/explanatory variables.

Univariate associations between ethnicity and primary outcome measures, and between ethnicity and key confounders/explanatory variables calculated using chi-squared tests for categorical variables, and t-tests and analyses of variance for continuous measures, with non-parametric equivalents used as appropriate for ordinal variables. This will enable the examination of the behavioural, social, and clinical phenotypes of the cohort in relation to the patterns of demographics, job role, attitudes to work and work climate, social and living circumstances, values and personality, and physical and mental health by ethnicity.

Using baseline data, multivariable analyses will test for associations between ethnicity and key outcomes, adjusted for the confounder/explanatory variables found to have a statistically significant univariate association with either ethnicity or the primary outcome variable(s), with interactions included as appropriate. Using follow-up data, multilevel models will be used to model changes over time by ethnic group.

Models will fit ethnicity as both a binary indicator (ethnic minority versus white) and as a categorical variable based on ONS categorisation of ethnicity, with the white group used as the reference group.

For peer review only

For peer review only - http://bmjopen.bmj.com/site/about/guidelines.xhtml

ETHICS AND DISSEMINATION

Ethical approval

The study has been approved by the Health Research Authority (Brighton and Sussex Research Ethics Committee; ethics reference: 20/HRA/4718).

Ethical considerations

Whilst this study is low risk, the questionnaire covers sensitive topics around COVID-19, ethnicity (including issues of discrimination and harassment), and mental health, and these could be distressing to participants. We aim to manage this risk by clearly indicating on the PIS that the questionnaire covers sensitive topics and that participants are under no obligation to answer these, or indeed any other, questions, and provide links to support organisations.

Participant confidentiality

The participants will be identified only by a unique identifier in the main research database. Identifiable information (name, date of birth, address etc) will be stored in a separate secure database, and will be accessed only by a small number of authorised staff at the University of Leicester who require access to administer the study. All documents will be stored securely, and only be accessible by study staff and authorised personnel. The study will comply with the Data Protection Act, which requires data to be anonymised as soon as it is practical to do so. Any dissemination of study findings will follow best-practice guidelines for deductive disclosure. Only aggregate data will included in publications.

Discontinuation/withdrawal of participants from study

Participants who wish to withdraw from the cohort study will be asked to determine the desired level of withdrawal from the study as described by the two options below. We will keep a record of consent for all participants to manage re-contact and for future audit. We will accept signed withdrawal forms from participants or, if they are unable to complete a withdrawal form themselves, from someone acting on the participant's behalf. At the present time, withdrawal forms will be completed electronically, but signed written forms will also be accepted when it is feasible to securely receive and store these. The options that participants will be given if they wish to withdraw:

Option 1 - No further contact: we would no longer contact the participant, but would have the participant's permission to continue to obtain information by accessing their health records in the future.

Option 2 – No further contact or access: we would no longer contact the participant or obtain information from the participant's health records in the future.

If participants withdraw from UK-REACH, then any data and samples already collected will remain and be used in the study. Information and data will continue to be collected about participants' health from central NHS records, hospital records, and participants' GPs, unless participants state otherwise on the withdrawal form.

Description of data flow

See Figure 3 for a description of the data flow.

For peer review only - http://bmjopen.bmj.com/site/about/guidelines.xhtml

Dissemination plan

Quarterly reports in months 3, 6, 9, and 12 summarising recruitment progress and initial findings on relationship between ethnicity, COVID-19 diagnosis and outcomes, physical/mental well-being, and professional and social factors. Brief reports will be produced and submitted for review by the stakeholder group (see Supplementary material for details), PEP, Study Steering Committee and the UK Government's Scientific Advisory Group for Emergencies (SAGE). Papers submitted to peer reviewed journals and pre-print servers.

DATA SHARING STATEMENT

To access data or samples produced by the UK-REACH study, the working group representative must first submit a data and material request form to the **Data Access Committee** (DAC) providing details for all manuscript proposals. The DAC will establish priorities for core and ancillary projects. For ancillary studies outside of the core deliverables, the **Steering Committee** will make final decisions once they have been approved by the **Core Management Group** and the DAC. Decisions on granting the access to data/materials will be made within eight weeks. Third party requests from outside the Project will require explicit approval of the Steering Committee once approved by the Core Management Group and the DAC.

AUTHORS CONTRIBUTIONS

MP conceived of the idea and led the application for funding with input from MT, KK, ICM, KW, RF, LN, SC, KRA, LG, AG and CJ. The survey was designed by KW, MP, ICM, CMel, CJ, AG, LN, CAM and RF. Online consent and survey tools were developed by LB. KW wrote the first draft of the manuscript with input from MP and all co-authors. All authors approved the submitted manuscript.

ACKNOWLEDGEMENTS

This work is carried out with the support of BREATHE -The Health Data Research Hub for Respiratory Health [MC_PC_19004] in partnership with SAIL Databank. BREATHE is funded through the UK Research and Innovation Industrial Strategy Challenge Fund and delivered through Health Data Research UK.

We wish to acknowledge the PEP group and the STAG group (see Supplementary material), and SERCO, as well as the following people for their support in organising the email invites from the regulatory bodies: Kerrin Clapton and Andrew Ledgard (General Medical Council), Caroline Kenny (Nursing and Midwifery Council), David Teeman and Lisa Bainbridge (General Dental Council), My Phan and John Tse (General Pharmaceutical Council), Angharad Jones (General Optical Council), Katherine Timms and Charlotte Rogers (The Health and Care Professions Council) and Mark Neale (Pharmaceutical Society of Northern Ireland).

FUNDING STATEMENT

UK-REACH is supported by a grant from the MRC-UK Research and Innovation (MR/V027549/1) and the Department of Health and Social Care through the National Institute for Health Research (NIHR) rapid response panel to tackle COVID-19. Core funding was also provided by NIHR Biomedical Research Centres.

For peer review only - http://bmjopen.bmj.com/site/about/guidelines.xhtml

ALG was funded by internal fellowships at the University of Leicester from the Wellcome Trust Institutional Strategic Support Fund (204801/Z/16/Z) and the BHF Accelerator Award (AA/18/3/34220). CJ holds a Medical Research Council Clinical Research Training Fellowship (MR/P00167X/1). KA is supported by Health Data Research (HDR) UK and as a NIHR Senior Investigator Emeritus (NF-SI-0512-10159). KK and LJG are supported by the National Institute for Health Research (NIHR) Applied Research Collaboration East Midlands (ARC EM). KK, MP, and RCF are supported by the NIHR Leicester Biomedical Research Centre (BRC). CAM is an NIHR Academic Clinical Fellow (ACF-2018-11-004). LBN is supported by an Academy of Medical Sciences Springboard Award (SBF005\1047). MP is supported by a NIHR Development and Skills Enhancement Award. MDT holds a Wellcome Trust Investigator Award (WT 202849/Z/16/Z) and an NIHR Senior Investigator Award. KW holds an NIHR Career Development Fellowship (CDF: CDF-2017-10-008). LVW holds a GSK/British Lung Foundation Chair in Respiratory Research (C17-1).

The views expressed in the publication are those of the author(s) and not necessarily those of the National Health Service (NHS), the NIHR or the Department of Health and Social Care.

This research was funded in whole, or in part, by the Wellcome Trust [WT204801/Z/16/Z and WT 202849/Z/16/Z]. For the purpose of open access, the author has applied a CC BY public copyright licence to any Author Accepted Manuscript version arising from this submission.

COMPETING INTERESTS STATEMENT

SC is Deputy Medical Director of the General Medical Council, UK Honorary Professor, University of Leicester. KK is Director of the University of Leicester Centre for Black Minority Ethnic Health, Trustee of the South Asian Health Foundation, Chair of the Ethnicity Subgroup of SAGE and Member of Independent SAGE. LVW receives grant funding from GSK and Orion, outside of the submitted work. KA has served as a paid consultant, providing unrelated methodological and strategic advice, to the pharmaceutical and life sciences industry generally and has received unrelated research funding from Association of the British Pharmaceutical Industry, European Federation of Pharmaceutical Industries & Associations, Pfizer, Sanofi and Swiss Precision Diagnostics. He is a Partner and Director of Visible Analytics Limited, a healthcare consultancy company.

WORD COUNT

4087

REFERENCES

1. Office for National Statistics. Coronavirus (COVID-19) related deaths by occupation, England and Wales: deaths registered between 9 March and 28 December 2020 2021 [Available from: https://www.ons.gov.uk/peoplepopulationandcommunity/healthandsocialcare/causesofdeath/bulletins/coronaviruscovid19relateddeathsbyoccupationenglandandwales/deathsregisteredbetween9marchand28december2020#deaths-involving-covid-19-among-men-and-women-health-and-social-care-workers.

2. Mutambudzi M, Niedwiedz C, Macdonald EB, Leyland A, Mair F, Anderson J, et al. Occupation and risk of severe COVID-19: prospective cohort study of 120 075 UK Biobank participants. Occupational and Environmental Medicine. 2020:oemed-2020-106731.

For peer review only

3. Levene LS, Coles B, Davies MJ, Hanif W, Zaccardi F, Khunti K. COVID-19 cumulative mortality rates for frontline healthcare staff in England. British Journal of General Practice. 2020;70(696):327-8.

4. Ayoubkhani D, Khunti K, Nafilyan V, Maddox T, Humberstone B, Diamond SI, et al. Epidemiology of post-COVID syndrome following hospitalisation with coronavirus: a retrospective cohort study. medRxiv. 2021:2021.01.15.21249885.

5. Khanijahani A. Racial, ethnic, and socioeconomic disparities in confirmed COVID-19 cases and deaths in the United States: a county-level analysis as of November 2020. Ethnicity & Health. 2020:1-14.

6. Khunti K, Singh AK, Pareek M, Hanif W. Is ethnicity linked to incidence or outcomes of covid-19? BMJ. 2020;369:m1548.

7. Kirby T. Evidence mounts on the disproportionate effect of COVID-19 on ethnic minorities. The Lancet Respiratory Medicine. 2020;8(6):547-8.

8. Platt L, Warwick R. COVID-19 and Ethnic Inequalities in England and Wales*. Fiscal Studies. 2020;41(2):259-89.

9. Webb Hooper M, Nápoles AM, Pérez-Stable EJ. COVID-19 and Racial/Ethnic Disparities. JAMA. 2020;323(24):2466-7.

10. Nazroo J, Murray K, Taylor H, Bécares L, Field Y, Kapadia D, et al. Rapid Evidence Review: Inequalities in relation to COVID-19 and their effects on London. University of Manchester, The Ubele Initiative, University of Sussex; 2020.

11. Sze S, Pan D, Nevill CR, Gray LJ, Martin CA, Nazareth J, et al. Ethnicity and clinical outcomes in COVID-19: A systematic review and meta-analysis. EClinicalMedicine. 2020;29-30:100630.

12. Pan D, Sze S, Minhas JS, Bangash MN, Pareek N, Divall P, et al. The impact of ethnicity on clinical outcomes in COVID-19: A systematic review. EClinicalMedicine. 2020;23.

13. Clift AK, Coupland CAC, Keogh RH, Diaz-Ordaz K, Williamson E, Harrison EM, et al. Living risk prediction algorithm (QCOVID) for risk of hospital admission and mortality from coronavirus 19 in adults: national derivation and validation cohort study. BMJ. 2020;371:m3731.

14. Sahebi A, Nejati B, Moayedi S, Yousefi K, Torres M, Golitaleb M. The prevalence of anxiety and depression among healthcare workers during the COVID-19 pandemic: An umbrella review of meta-analyses. Progress in Neuro-Psychopharmacology and Biological Psychiatry. 2021:110247.

15. Galli F, Pozzi G, Ruggiero F, Mameli F, Cavicchioli M, Barbieri S, et al. A Systematic Review and Provisional Metanalysis on Psychopathologic Burden on Health Care Workers of Coronavirus Outbreaks. Frontiers in Psychiatry. 2020;11(1001).

For peer review only - http://bmjopen.bmj.com/site/about/guidelines.xhtml

16. Greene T, Harju-Seppänen J, Adeniji M, Steel C, Grey N, Brewin CR, et al. Predictors and rates of PTSD, depression and anxiety in UK frontline health and social care workers during COVID-19. medRxiv. 2020:2020.10.21.20216804.

17. Iob E, Frank P, Steptoe A, Fancourt D. Levels of Severity of Depressive Symptoms Among At-Risk Groups in the UK During the COVID-19 Pandemic. JAMA Network Open. 2020;3(10):e2026064-e.

18. Iob E, Steptoe A, Fancourt D. Abuse, self-harm and suicidal ideation in the UK during the COVID-19 pandemic. The British Journal of Psychiatry. 2020;217(4):543-6.

19. Wanigasooriya K, Palimar P, Naumann DN, Ismail K, Fellows JL, Logan P, et al. Mental health symptoms in a cohort of hospital healthcare workers following the first peak of the COVID-19 pandemic in the UK. BJPsych Open. 2020;7(1):e24.

20. Trivedy C, Mills I, Dhanoya O. The impact of the risk of COVID-19 on Black, Asian and Minority Ethnic (BAME) members of the UK dental profession. British Dental Journal. 2020;228(12):919-22.

21. Martin CA, Patel P, Goss C, Jenkins DR, Price A, Barton L, et al. Demographic and occupational determinants of anti-SARS-CoV-2 IgG seropositivity in hospital staff. Journal of Public Health. 2020.

22. Drew DA, Nguyen LH, Steves CJ, Menni C, Freydin M, Varsavsky T, et al. Rapid implementation of mobile technology for real-time epidemiology of COVID-19. Science. 2020;368(6497):1362-7.

23. Herdman M, Gudex C, Lloyd A, Janssen MF, Kind P, Parkin D, et al. Development and preliminary testing of the new five-level version of EQ-5D (EQ-5D-5L). Quality of Life Research. 2011;20(10):1727-36.

24. Löwe B, Kroenke K, Gräfe K. Detecting and monitoring depression with a two-item questionnaire (PHQ-2). Journal of Psychosomatic Research. 2005;58(2):163-71.

25. Plummer F, Manea L, Trepel D, McMillan D. Screening for anxiety disorders with the GAD-7 and GAD-2: a systematic review and diagnostic metaanalysis. General Hospital Psychiatry. 2016;39:24-31.

26. Lang AJ, Wilkins K, Roy-Byrne PP, Golinelli D, Chavira D, Sherbourne C, et al. Abbreviated PTSD Checklist (PCL) as a guide to clinical response. General hospital psychiatry. 2012;34(4):332-8.

27. Hughes ME, Waite LJ, Hawkley LC, Cacioppo JT. A Short Scale for Measuring Loneliness in Large Surveys:Results From Two Population-Based Studies. Research on Aging. 2004;26(6):655-72.

28. Surveys using our four personal well-being questions: A guide to what surveys include the four ONS personal well-being questions: Office for National Statistics; 2018 [Available from: https://www.ons.gov.uk/peoplepopulationandcommunity/wellbeing/methodologies/surveysusingthe4officefornationalstatisticspersonalwellbeingquestions.

For peer review only - http://bmjopen.bmj.com/site/about/guidelines.xhtml

29. Schaufeli WB, Bakker AB, Salanova M. The Measurement of Work Engagement With a Short Questionnaire:A Cross-National Study. Educational and Psychological Measurement. 2006;66(4):701-16.

30. General Medical Council National training surveys 2019: Initial findings report. General Medical Council, 2019.

31. Kristensen TS, Borritz M, Villadsen E, Christensen KB. The Copenhagen Burnout Inventory: A new tool for the assessment of burnout. Work & Stress. 2005;19(3):192-207.

32. Harmonised Concepts and Questions for Social Data Sources, Primary Principles: Ethnic Group. Government Statistical Service; 2016.

33. Ladher N. Let's talk about race: BMJ; 2020 [Available from: https://blogs.bmj.com/bmj/2020/02/13/navjoyt-ladher-lets-talk-about-race/.

For peer review only

For peer review only - http://bmjopen.bmj.com/site/about/guidelines.xhtml

Figure captions

Figure 1: Study flowchart

Figure 2: UK-REACH Work package 2 timeline as of 4th February 2021. Dates are shown across the bottom from October 2019 to July 2021. The COVID-19 outbreak began in the UK in January 2020 with numbers of daily hospital admissions due to COVID-19 shown by the lower orange line for Wave one and Wave two. Vaccinations began in December 2020 and are shown by the green line for daily vaccinations. Lockdowns are shown by horizontal red bars, the first national lockdown beginning on March 23rd 2020, the second on November 5th, and the third of January 5th 2021. Lockdowns differed somewhat in timing between England, Wales, Scotland and Northern Ireland. Tiered local restrictions were in place in various regions of the UK between lockdowns, shown in yellow. Questionnaire 1 of Work package 2 began to be distributed on December 4th onwards and is being distributed until the end of March 2021. Questionnaire 1 asks about current events and working conditions, as well as retrospectively about events and working conditions pre-Covid in 2019, about early Covid responses in the first months of 2021, and about events during the first national lockdown. Questionnaire 2 will be distributed four months after registration for questionnaire 1 and therefore will be distributed between April and June 2021. Questionnaire 2 asks primarily about current working conditions, and changes to other aspects of participants' lives captured in Questionnaire 1, including key measures of physical and mental health. With consent, the questionnaire data will be linked to electronic healthcare record data.

Figure 3: Data flow diagram. Solid lines with a circle indicate identifiable data flows for linkage purposes only. Solid lines with an arrow indicate anonymous outputs. Dashed lines indicate de-identified data. After consenting to join UK-REACH, participants provide limited personal identifiable information (PII) which are stored in a secure location at the University of Leicester, alongside study ID. Questionnaire data (including study ID but not alongside the aforementioned PII) are stored in a different secure location at the University of Leicester. Participants provide consent for the study to follow their health by extracting information from electronic health records. Relevant PII for each participant will be securely transferred to NHS Wales Informatics Service (alongside the UK-REACH study ID) in order to acquire NHS number/Community Health Index (CHI) number (for Scotland)/Health and Care number (for Northern Ireland). NHS/CHI/H&C number will then be used to link to healthcare records by the relevant data provider across the UK. De-identified health records will be sent directly to the Secure Anonymised Information Linkage databank (SAIL, https://saildatabank.com), retaining Study ID but excluding PII. Questionnaire data (including study ID) will be sent from the University of Leicester to SAIL. These datasets will be linked within SAIL to provide the complete dataset. Interim analysis of unlinked questionnaire data will be performed at the University of Leicester using pseudonymised datasets. All linked data analysis will take place within SAIL. The above diagram and style was interpreted by Anna Guyatt and Chris Orton from an initial data flow diagram created and provided by Andy Boyd at the University of Bristol. It has been repurposed and amended to illustrate data flows specific to the UK-REACH project.

For peer review only - http://bmjopen.bmj.com/site/about/guidelines.xhtml

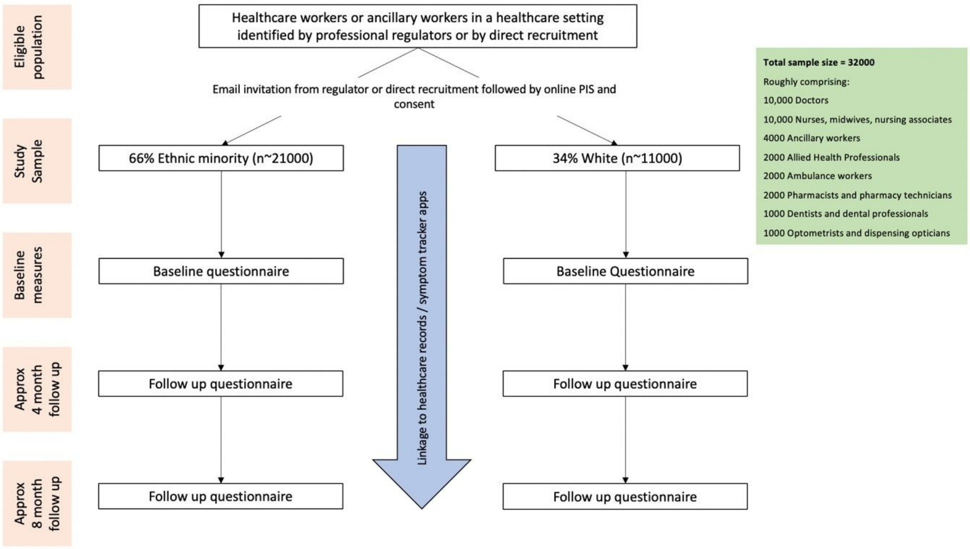

Figure 1: Study flowchart

116x65mm (300 x 300 DPI)

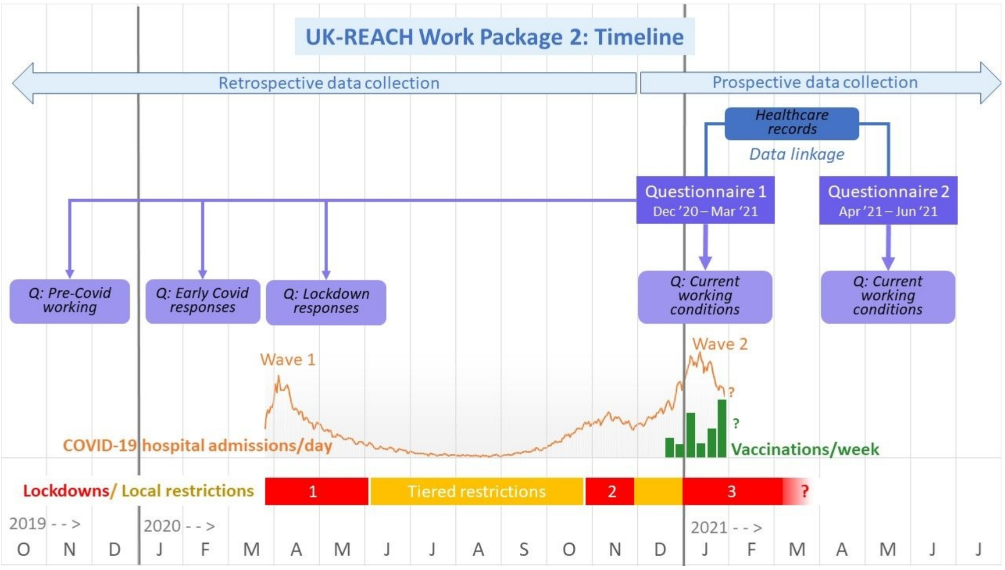

Figure 2: UK-REACH Work package 2 timeline as of 4th February 2021. Dates are shown across the bottom from October 2019 to July 2021. The COVID-19 outbreak began in the UK in January 2020 with numbers of daily hospital admissions due to COVID-19 shown by the lower orange line for Wave one and Wave two. Vaccinations began in December 2020 and are shown by the green line for daily vaccinations. Lockdowns are shown by horizontal red bars, the first national lockdown beginning on March 23rd 2020, the second on November 5th, and the third of January 5th 2021. Lockdowns differed somewhat in timing between England, Wales, Scotland and Northern Ireland. Tiered local restrictions were in place in various regions of the UK between lockdowns, shown in yellow. Questionnaire 1 of Work package 2 began to be distributed on December 4th onwards and is being distributed until the end of March 2021. Questionnaire 1 asks about current events and working conditions, as well as retrospectively about events and working conditions pre-Covid in 2019, about early Covid responses in the first months of 2021, and about events during the first national lockdown. Questionnaire 2 will be distributed four months after registration for questionnaire 1 and therefore will be distributed between April and June 2021. Questionnaire 2 asks primarily about current working conditions, and changes to other aspects of participants' lives captured in Questionnaire 1, including key measures of physical and mental health. With consent, the questionnaire data will be linked to electronic healthcare record data.

108x60mm (300 x 300 DPI)

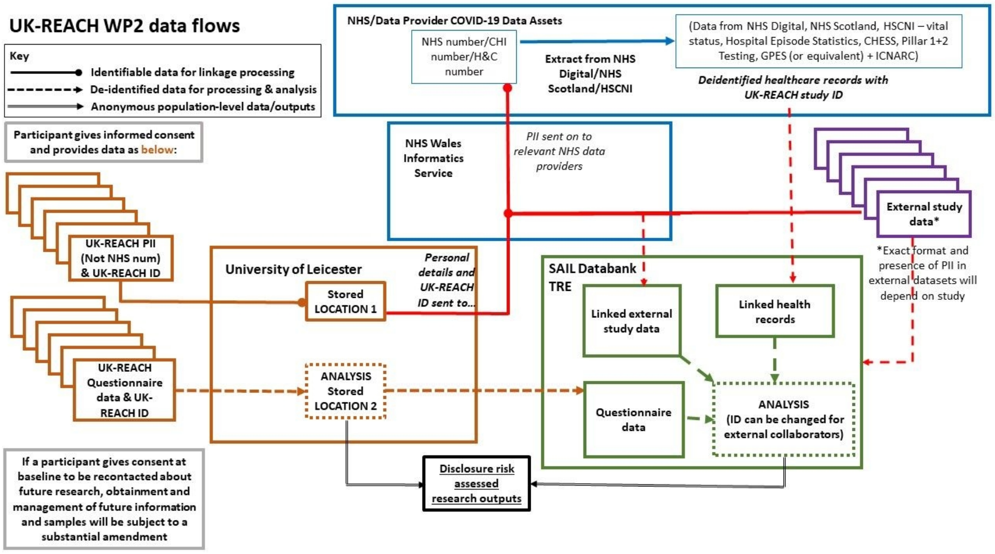

Figure 3: Data flow diagram. Solid lines with a circle indicate identifiable data flows for linkage purposes only. Solid lines with an arrow indicate anonymous outputs. Dashed lines indicate de-identified data. After consenting to join UK-REACH, participants provide limited personal identifiable information (PII) which are stored in a secure location at the University of Leicester, alongside study ID. Questionnaire data (including study ID but not alongside the aforementioned PII) are stored in a different secure location at the University of Leicester. Participants provide consent for the study to follow their health by extracting information from electronic health records. Relevant PII for each participant will be securely transferred to NHS Wales Informatics Service (alongside the UK-REACH study ID) in order to acquire NHS number/Community Health Index (CHI) number (for Scotland)/Health and Care number (for Northern Ireland). NHS/CHI/H&C number will then be used to link to healthcare records by the relevant data provider across the UK. De-identified health records will be sent directly to the Secure Anonymised Information Linkage databank (SAIL, https://saildatabank.com), retaining Study ID but excluding PII. Questionnaire data (including study ID) will be sent from the University of Leicester to SAIL. These datasets will be linked within SAIL to provide the complete dataset. Interim analysis of unlinked questionnaire data will be performed at the University of Leicester using pseudonymised datasets. All linked data analysis will take place within SAIL. The above diagram and style was interpreted by Anna Guyatt and Chris Orton from an initial data flow diagram created and provided by Andy Boyd at the University of Bristol. It has been repurposed and amended to illustrate data flows specific to the UK-REACH project.

108x60mm (300 x 300 DPI)

SUPPLEMENTARY INFORMATION

List of Members of UK-REACH Stakeholder Group (STAG) as at January 2021

1. General Medical Council (GMC)
2. Nursing and Midwifery Council (NMC)
3. General Dental Council (GDC)
4. General Pharmaceutical Council (GPC)
5. Royal College of Psychiatrists (RCPsych)
6. Royal College of Obstetricians and Gynaecologists (RCOG)
7. Royal College of Midwives (RCM)
8. NHS Confederation
9. British Association of Physicians of Indian Origin (BAPIO)
10. Sudan Doctors' Union –UK Branch
11. Association of Pakistani Physicians of Northern Europe (APPNE)
12. South Asian Health Foundation (SAHF)
13. Health Education England (HEE)
14. General Optical Council (GOC)
15. Filipino Nurses Association UK (FNAUK)
16. Pharmaceutical Society of Northern Ireland (PSNI)
17. Health and Care Professions Council (HCPC)
18. NHS England & Improvement
19. British Medical Association (BMA)
20. Medical Association of Nigerians Across Great Britain (MANSAG)

List of Members of UK-REACH Professional Expert Panel (PEP) as at January 2021

Susie Lagrata (co-Chair), Nurse.
Padmasayee Papineni (co-Chair), Doctor.
Sandra Kazembe, Nurse.
Tatiana Monteiro, Domestic worker.
Juliette Mutumiyana, Doctor.
Satheesh Mathew, Doctor.
Amir Burney, Doctor.
Ahmed Hashim, Doctor.
Tiffanie Harrison, Nurse.

For peer review only

STROBE Statement—Checklist of items that should be included in reports of ***cohort studies***

	Item No	Recommendation	Page No.
**Title and abstract**	1	(*a*) Indicate the study's design with a commonly used term in the title or the abstract	1
		(*b*) Provide in the abstract an informative and balanced summary of what was done and what was found	2
**Introduction**			
Background/rationale	2	Explain the scientific background and rationale for the investigation being reported	4
Objectives	3	State specific objectives, including any prespecified hypotheses	4,5
**Methods**			
Study design	4	Present key elements of study design early in the paper	5
Setting	5	Describe the setting, locations, and relevant dates, including periods of recruitment, exposure, follow-up, and data collection	5-7
Participants	6	(*a*) Give the eligibility criteria, and the sources and methods of selection of participants. Describe methods of follow-up	5,6
		(*b*) For matched studies, give matching criteria and number of exposed and unexposed	NA
Variables	7	Clearly define all outcomes, exposures, predictors, potential confounders, and effect modifiers. Give diagnostic criteria, if applicable	8-10
Data sources/ measurement	8*	For each variable of interest, give sources of data and details of methods of assessment (measurement). Describe comparability of assessment methods if there is more than one group	8-10
Bias	9	Describe any efforts to address potential sources of bias	10
Study size	10	Explain how the study size was arrived at	6
Quantitative variables	11	Explain how quantitative variables were handled in the analyses. If applicable, describe which groupings were chosen and why	10
Statistical methods	12	(*a*) Describe all statistical methods, including those used to control for confounding	10
		(*b*) Describe any methods used to examine subgroups and interactions	10
		(*c*) Explain how missing data were addressed	NA
		(*d*) If applicable, explain how loss to follow-up was addressed	NA
		(*e*) Describe any sensitivity analyses	NA
**Results**			
Participants	13*	(a) Report numbers of individuals at each stage of study—eg numbers potentially eligible, examined for eligibility, confirmed eligible, included in the study, completing follow-up, and analysed	NA
		(b) Give reasons for non-participation at each stage	NA
		(c) Consider use of a flow diagram	NA
Descriptive data	14*	(a) Give characteristics of study participants (eg demographic, clinical, social) and information on exposures and potential confounders	NA
		(b) Indicate number of participants with missing data for each variable of interest	NA
		(c) Summarise follow-up time (eg, average and total amount)	NA
Outcome data	15*	Report numbers of outcome events or summary measures over time	NA
Main results	16	(*a*) Give unadjusted estimates and, if applicable, confounder-adjusted estimates and their precision (eg, 95% confidence interval). Make clear	NA

For peer review only - http://bmjopen.bmj.com/site/about/guidelines.xhtml

		which confounders were adjusted for and why they were included	
		(*b*) Report category boundaries when continuous variables were categorized	NA
		(*c*) If relevant, consider translating estimates of relative risk into absolute risk for a meaningful time period	NA
Other analyses	17	Report other analyses done—eg analyses of subgroups and interactions, and sensitivity analyses	NA
**Discussion**			
Key results	18	Summarise key results with reference to study objectives	NA
Limitations	19	Discuss limitations of the study, taking into account sources of potential bias or imprecision. Discuss both direction and magnitude of any potential bias	NA
Interpretation	20	Give a cautious overall interpretation of results considering objectives, limitations, multiplicity of analyses, results from similar studies, and other relevant evidence	NA
Generalisability	21	Discuss the generalisability (external validity) of the study results	NA
**Other information**			
Funding	22	Give the source of funding and the role of the funders for the present study and, if applicable, for the original study on which the present article is based	13

*Give information separately for exposed and unexposed groups.

Note: An Explanation and Elaboration article discusses each checklist item and gives methodological background and published examples of transparent reporting. The STROBE checklist is best used in conjunction with this article (freely available on the Web sites of PLoS Medicine at http://www.plosmedicine.org/, Annals of Internal Medicine at http://www.annals.org/, and Epidemiology at http://www.epidem.com/). Information on the STROBE Initiative is available at http://www.strobe-statement.org.

For peer review only

BMJ Open

The United Kingdom Research study into Ethnicity And COVID-19 outcomes in Healthcare workers (UK-REACH): Protocol for a prospective longitudinal cohort study of healthcare and ancillary workers in UK healthcare settings

Journal:	*BMJ Open*
Manuscript ID	bmjopen-2021-050647.R1
Article Type:	Protocol
Date Submitted by the Author:	02-Aug-2021

Complete List of Authors:	Woolf, Katherine; University College London, Research Department of Medical Education<br>Melbourne, Carl; University of Leicester, Genetic Epidemiology Research Group, Department of Health Sciences<br>Bryant, Luke; University of Leicester, Department of Respiratory Sciences<br>Guyatt, Anna; University of Leicester, Genetic Epidemiology Research Group, Department of Health Sciences<br>McManus, Ian; University College London, Research Department of Medical Education<br>Gupta, Amit; Oxford University Hospitals NHS Foundation Trust<br>Free, Robert; University of Leicester, Department of Respiratory Sciences; NIHR Leicester Biomedical Research Centre Respiratory Diseases<br>Nellums, Laura ; University of Nottingham, Division of Epidemiology and Public Health<br>Carr, Sue; University Hospitals of Leicester NHS Trust; General Medical Council<br>John, Catherine; University of Leicester, Department of Health Sciences<br>Martin, Christopher; University of Leicester, Department of Respiratory Sciences; University Hospitals of Leicester NHS Trust, Department of Infection and HIV Medicine<br>Wain, Louise; University of Leicester, Department of Health Sciences; NIHR Leicester Biomedical Research Centre Respiratory Diseases<br>Gray, Laura; University of Leicester, Department of Health Sciences<br>Garwood, Claire; University of Leicester, Department of Respiratory Sciences<br>Modhwadia, Vishant; University of Leicester, Department of Respiratory Sciences<br>Abrams, Keith; University of York, Centre for Health Economics<br>Tobin, Martin; University of Leicester, Genetic Epidemiology Research Group, Department of Health Sciences; NIHR Leicester Biomedical Research Centre Respiratory Diseases, Glenfield Hospital<br>Khunti, Kamlesh; University of Leicester, Diabetes Research Centre<br>Pareek, Manish; University of Leicester, Department of Respiratory Sciences; University Hospitals of Leicester NHS Trust, Department of Infection and HIV Medicine

**Primary Subject Heading**:	Infectious diseases
Secondary Subject Heading:	Public health, Health services research
Keywords:	Public health < INFECTIOUS DISEASES, MENTAL HEALTH, COVID-19

SCHOLARONE™ Manuscripts

I, the Submitting Author has the right to grant and does grant on behalf of all authors of the Work (as defined in the below author licence), an exclusive licence and/or a non-exclusive licence for contributions from authors who are: i) UK Crown employees; ii) where BMJ has agreed a CC-BY licence shall apply, and/or iii) in accordance with the terms applicable for US Federal Government officers or employees acting as part of their official duties; on a worldwide, perpetual, irrevocable, royalty-free basis to BMJ Publishing Group Ltd ("BMJ") its licensees and where the relevant Journal is co-owned by BMJ to the co-owners of the Journal, to publish the Work in this journal and any other BMJ products and to exploit all rights, as set out in our licence.

The Submitting Author accepts and understands that any supply made under these terms is made by BMJ to the Submitting Author unless you are acting as an employee on behalf of your employer or a postgraduate student of an affiliated institution which is paying any applicable article publishing charge ("APC") for Open Access articles. Where the Submitting Author wishes to make the Work available on an Open Access basis (and intends to pay the relevant APC), the terms of reuse of such Open Access shall be governed by a Creative Commons licence – details of these licences and which Creative Commons licence will apply to this Work are set out in our licence referred to above.

Other than as permitted in any relevant BMJ Author's Self Archiving Policies, I confirm this Work has not been accepted for publication elsewhere, is not being considered for publication elsewhere and does not duplicate material already published. I confirm all authors consent to publication of this Work and authorise the granting of this licence.

For peer review only

The United Kingdom Research study into Ethnicity And COVID-19 outcomes in Healthcare workers (UK-REACH): Protocol for a prospective longitudinal cohort study of healthcare and ancillary workers in UK healthcare settings

Katherine Woolf*[1], Carl Melbourne*[2], Luke Bryant*[3], Anna L Guyatt[2], Ian Chris McManus[1], Amit Gupta[4], Robert C Free[3,9], Laura Nellums[5], Sue Carr[6,7], Catherine John[2], Christopher A Martin[3,8], Louise V Wain[2,9], Laura J Gray[2], Claire Garwood[3], Vishant Modhwadia[3], Keith Abrams [10], Martin D Tobin[2,9], Kamlesh Khunti[11], Manish Pareek[3,8]** on behalf of the UK-REACH Study Collaborative Group+

*Joint first authors; **Corresponding author manish.pareek@leicester.ac.uk

Affiliations

1 Research Department of Medical Education, University College London Medical School, Gower Street, London WC1E 6BT, UK.

2 Department of Health Sciences, University of Leicester, University Road, Leicester, LE1 7RH, UK.

3 Department of Respiratory Sciences, University of Leicester, Leicester, LE1 7RH, UK.

4 Oxford University Hospitals NHS Foundation Trust, Headley Way, Headington, Oxford OX3 9DU, UK.

5 Division of Epidemiology and Public Health, School of Medicine, University of Nottingham, City Hospital Campus, Hucknall Road, NG5 1PB, UK.

6 University Hospitals Leicester NHS Trust, Level 3, Balmoral Building, Leicester Royal Infirmary, Infirmary Square, Leicester, LE1 5WW, UK.

7 General Medical Council, 3BH, 350 Euston Rd, London NW1 3JN, UK.

8 Department of Infection and HIV Medicine, University Hospitals of Leicester NHS Trust, Leicester, LE1 5WW, UK.

9 National Institute for Health Research Leicester Biomedical Research Centre - Respiratory, Glenfield Hospital, Leicester, LE3 9QP UK.

10 Centre for Health Economics, University of York, York, UK.

11 Diabetes Research Centre, University of Leicester, Leicester LE5 4PW, UK

UK-REACH Study Collaborative Group (*Work Package 2 collaborators):

Manish Pareek (Chief investigator)*, Amani Al-Oraibi, Amit Gupta**, Anna L Guyatt, Carl Melbourne*, Catherine John*, Christopher A Martin*, Ian Chris McManus*, Chris Orton, Claire

Garwood*, David Ford, Edward Dove, Fatimah Wobi, Janet Hood, Kamlesh Khunti*, Katherine Woolf*, Keith Abrams*, Laura J Gray*, Laura Nellums*, Louise V Wain*, Lucy Teece, Luke Bryant*, Martin Tobin,* Mayuri Gogoi, Osama Hassan, Robert C Free*, Ruby Reed-Berendt, Sue Carr*, Vishant Modhwadia*.

Abstract

Introduction

The COVID-19 pandemic has resulted in significant morbidity and mortality, and devastated economies globally. Among groups at increased risk are healthcare workers (HCWs) and ethnic minority groups. Emerging evidence suggests HCWs from ethnic minority groups are at increased risk of adverse COVID-19-related outcomes. To date there has been no large-scale analysis of these risks in UK HCWs or ancillary workers in healthcare settings, stratified by ethnicity or occupation, and adjusted for confounders. This paper reports the protocol for a prospective longitudinal questionnaire study of UK HCWs, as part of the UK-REACH programme (The United Kingdom Research study into Ethnicity And COVID-19 outcomes in Healthcare workers).

Methods and analysis

A baseline questionnaire will be administered to a national cohort of UK HCWs and ancillary workers in healthcare settings, and those registered with UK healthcare regulators, with follow-up questionnaires administered at 4 and 8 months. With consent, questionnaire data will be linked to health records with 25 year follow-up.

Univariate associations between ethnicity and clinical COVID-19 outcomes, physical and mental health, and key confounders/explanatory variables will be tested. Multivariable analyses will test for associations between ethnicity and key outcomes adjusted for the confounder/explanatory variables. We will model changes over time by ethnic group, facilitating understanding of absolute and relative risks in different ethnic groups, and generalisability of findings.

Ethics and dissemination.

The study is approved by Health Research Authority (reference 20/HRA/4718), and carries minimal risk. We aim to manage the small risk of participant distress about questions on sensitive topics by clearly participant information that the questionnaire covers sensitive topics and there is no obligation to answer these or any other questions, and by providing support organisation links. Results will be disseminated with reports to Government and papers submitted to pre-print servers and peer reviewed journals.

Registration details.

Trial ID: ISRCTN11811602

For peer review only - http://bmjopen.bmj.com/site/about/guidelines.xhtml

STRENGTHS AND LIMITATIONS OF THIS STUDY

- Sampling frame includes a variety of healthcare worker job roles including ancillary workers in healthcare settings will improve the generalisability of results across diverse healthcare job roles.
- Longitudinal study including three waves of questionnaire data collection, and linkage to administrative data over 25 years, with consent, will enable researchers to infer causal relationships.
- Unique support from all major UK healthcare worker regulators, relevant healthcare worker organisations, and a Professional Expert Panel to increase participant uptake and the validity of findings.
- Potential for self-selection bias and low response rates.
- The use of electronic invitations and online data collection makes it harder to reach ancillary workers without regular access to work email addresses.

For peer review only

For peer review only - http://bmjopen.bmj.com/site/about/guidelines.xhtml

INTRODUCTION

COVID-19 has spread rapidly across the world, causing significant morbidity and mortality, and devastating health economies in many countries. Healthcare workers (HCWs) have been identified as being at increased risk of SARS-CoV-2 infection and adverse outcomes,(1-3) as have individuals from ethnic minority groups.(2, 4-13) Emerging evidence suggests that ethnic minority groups may also be at greater risk of long-term COVID-19 sequelae.(4) HCWs and individuals from ethnic minority groups may also be at increased risk of COVID-19-related poor mental health outcomes, including anxiety, depression and post-traumatic stress.(14-19)

There are concerns that HCWs from ethnic minority groups are at particular risk of SARS-CoV-2 (severe acute respiratory syndrome coronavirus 2) infection and adverse COVID-19 outcomes compared to white HCWs (3, 20, 21) However, the quality of data relating to COVID-19 outcomes in HCWs remains poor, with no large representative studies of the risk of COVID-19 infection or adverse outcomes in healthcare workers or ancillary workers in healthcare settings (hereafter "HCWs") stratified by ethnicity or occupation type, controlling for potential confounders.

To address this, UK-REACH (United Kingdom Research study into COVID-19 outcomes in Healthcare workers) will rapidly examine differences in COVID-19 diagnosis, clinical outcomes (diagnosis, hospitalisation, ICU admission), professional roles, and well-being among ethnic minority and white HCWs through a unique large database analysis (rapid linkage and analysis of anonymised professional registration and National Health Service (NHS) datasets), longitudinal cohort study, legal/ethical analysis, and qualitative work packages. This work will provide information on very short-term outcomes and produce rapid actionable outputs, as well as enabling investigations of the medium/long-term effects of COVID-19 on HCWs in future studies through the linkage and cohort study. This protocol describes the UK-REACH longitudinal cohort study.

Research Question

What is the impact of COVID-19 on the physical and mental health of ethnic minority HCWs compared to white HCWs in the short term and the longer term?

Aims

To examine the relationship between ethnicity and COVID-19-related mental and physical health outcomes through the establishment of a novel longitudinal cohort study of HCWs, including recruitment from the membership bodies and professional registers for different healthcare roles, providers of facilities management, and directly from UK healthcare settings.

To study changes in health outcomes, social circumstances and professional roles of HCWs of different ethnicities, over the course of the COVID-19 pandemic and to characterise longer-term outcomes and consequences.

To measure differences in the impact of COVID-19 infection and working during the pandemic on physical and mental health in a multi-ethnic group of HCWs in the UK.

Objectives

To survey HCWs at baseline to collect data on demographics, job role, attitudes to work and work climate, social and living circumstances, values and personality, and physical and mental health.

To collect baseline biological samples for future analysis in a subsample of consenting participants.

For peer review only - http://bmjopen.bmj.com/site/about/guidelines.xhtml

To conduct follow-up surveys and samples over 12 months in order to capture changes over subsequent COVID-19 pandemic waves.

To link survey data to participant healthcare records, with consent.

METHODS AND ANALYSIS

Study design

National prospective longitudinal cohort study in all four nations of the United Kingdom.

Setting

Healthcare workers and ancillary workers in healthcare settings within the UK.

Participants

Inclusion criteria

Age ≥ 16 years;

Living in the UK;

Healthcare worker or ancillary worker in a UK healthcare setting OR Registered with the following UK healthcare professional regulatory bodies: the General Medical Council (GMC), Nursing and Midwifery Council (NMC), General Dental Council (GDC), Health and Care Professions Council (HCPC), General Optical Council (GOC), General Pharmaceutical Council (GPC) or the Pharmaceutical Society of Northern Ireland (PSNI);

Willing and able to give informed consent.

Exclusion criteria

Age <16 years;

Living outside the UK;

Not a healthcare worker or ancillary worker in a healthcare setting AND not regulated by one of the professional regulatory bodies listed above;

Unwilling and/or unable to give informed consent.

Sample size

We aim to recruit at least 32,000 HCWs (66% from ethnic minority groups). See Figure 1 for the study flow chart. The proposed sample will approximately comprise:

- 10,000 doctors
- 10,000 nurses, midwives and nursing associates
- 4,000 ancillary workers
- 2,000 allied health professionals
- 2,000 ambulance workers
- 2,000 pharmacists and pharmacy technicians
- 1,000 dentists and dental care professionals
- 1,000 optometrists and dispensing opticians

For peer review only - http://bmjopen.bmj.com/site/about/guidelines.xhtml

For peer review only

Whilst the above numbers represent our target numbers for recruitment, we will welcome participants working in other roles within healthcare settings. We may adapt sampling frame for subgroups following initial response, to ensure we have appropriate representation of ethnic minority participants in each job role.

This sample size has been pragmatically chosen to allow for adequate representation of all ethnic groups within all job-role categories. Based on general population prevalence estimates (11) producing conservative power estimates, we anticipate at least 80% (statistical) power to detect modest effects of ethnicity (RR ≥1.5) for more prevalent outcomes (e.g. COVID-19 diagnosis) and larger effects (RR ≥2) for rarer outcomes (e.g. mortality). Power calculations will be reviewed to reflect changing rates of infection.

Recruitment

Recruitment will be via several routes, and will be incentivised by the inclusion of a prize draw for those who complete the questionnaire:

i) Email, letter, and/or text message invitations from healthcare professional regulators.
The records and registrations with the GMC, NMC, GDC, HCPC, GOC, GPC, PSNI will be used as a sampling frame. Where possible, we will utilise demographic data routinely collected by regulators to oversample for people identifying as being from ethnic minority backgrounds. We will endeavour to sample representatively across age groups, sex/gender, job roles, and other characteristics, in order to maximise the generalisability of our findings. Regulators will send invitations and reminders on behalf of the principal investigator. Alongside these invites regulators will also promote the study through their social media channels as the invites go out.

ii) Targeted advertisement to key staff groups through healthcare organisations.
We will advertise the study through the general communication channels of regulators, professional bodies (e.g. Royal College of Midwives, Royal College of General Practitioners), Health Education bodies, and other relevant organisations (e.g. the British Medical Association, the British Association of Physicians of Indian Origin (BAPIO), The Filipino Nurses Association United Kingdom). This will include promotion through newsletters, web pages, email communications and banners on self-service portals (for example, on the NHS Electronic Staff Record portal used for accessing payslips).

iii) General publicity of the study via print and broadcast media, social media for the study and other relevant organisations, and posters or flyers in workplaces, as relevant to participant staff groups.

iv) Direct invitation and recruitment via UK healthcare providers.
UK healthcare providers, including at least 30 NHS Hospital Trusts, will advertise to potential participants by email, text, mail, verbally, or through posters/flyers. Trusts will be selected to represent a range of geographical areas (to include England, Scotland, Wales, and Northern Ireland). We will aim to recruit from both large teaching hospitals and smaller community healthcare trusts, and will take into consideration regional ethnic diversity when selecting trusts. We will also utilise a study infographic to promote recruitment through NHS trusts. Recruitment of ancillary staff has been facilitated by Serco at specific trusts, through utilisation of content handouts and posters which have been cascaded by contractors on site.

For peer review only - http://bmjopen.bmj.com/site/about/guidelines.xhtml
For peer review only

Invitations and advertisements will direct staff via a weblink and/or QR code to the study recruitment site. In the case of direct recruitment through UK healthcare settings, potential participants will be supported to join with the help of suitably trained members of the local research team where appropriate. Reminders will also be sent to improve recruitment to the study.

Data collection

See Figure 2 for the study timeline.

To gain informed consent, potentially interested participants will have the opportunity to read the UK-REACH participant information sheet (PIS) online via a web application, or in person with a member of the local research team, before deciding if they would like to take part. Potential participants will be asked to register for a UK-REACH account (https://uk-reach.org/) by entering an email address and password, then will be asked to sign an online consent form. To complete registration, participants will be asked to provide personal details including name, date of birth, and address. Participants will be considered enrolled into the study after completing the registration process, and giving their electronic consent on a secure web page. They will be assigned a unique identifier at this point. This will be securely retained in an electronic database at the University of Leicester, which will also function as an enrolment log.

Once translation has been undertaken and checked, we will make study materials (PIS and consent form) available in alternative languages commonly used by workers in healthcare settings as required. These will be made available on the UK-REACH website.

After consenting to participation in the study, participants will be asked to complete a baseline online questionnaire on demographics, job role and other work circumstances, location of work/residence, interaction with COVID-19 patients (including access to personal protective equipment , social and living circumstances, discrimination and harassment, values and personality, and physical and mental health (see Measures, below). This questionnaire will be accessible via the study website, and support to complete the questionnaire will also be available from the study team. Some identifiable data (e.g. name, DOB, address) will be collected during registration and/or the questionnaire to facilitate secure data linkage (see Data linkage, below).

Participants will be invited to complete the baseline questionnaire between December 2020 and January 2021. Participants will have the option of completing two further questionnaires, one at approximately 4 months from baseline and one at approximately 8 months from baseline. Consent will be requested to follow up participants for up to 25 years (subject to funding), and during this time serial questionnaire data will be collected, and periodic linkage with healthcare records will take place (see Data linkage, below).

Data linkage

Participants will be asked to provide consent for the study to follow their health by extracting information from their past and future NHS health care records (including NHS number), any COVID-19 related records, and from "COVID-19 symptom study" websites or apps (22) if they use them.

Initially, questionnaire and personal data, for which consent has been given, will be electronically transferred to University of Leicester, and stored separately on secure servers. A study ID for each participant together with identifiable data will be sent to NHS Wales Informatics Service in order to acquire the list of NHS numbers/Community Health Index (CHI) number (for Scotland)/Health and

For peer review only - http://bmjopen.bmj.com/site/about/guidelines.xhtml

Care number (for Northern Ireland) in partnership with the relevant UK NHS data providers. Study ID and NHS/CHI/H&C number will then be used to link to healthcare records by the relevant data provider across the UK. De-identified records will be sent directly to the Secure Anonymised Information Linkage databank (SAIL, https://saildatabank.com), retaining Study ID. Separately, study ID and corresponding questionnaire data will be sent from the University of Leicester to SAIL. These datasets will be linked within SAIL to provide the complete dataset. Interim analysis of unlinked questionnaire data will be performed at the University of Leicester using pseudonymised datasets. All linked data analysis will take place within SAIL.

Questionnaire design

The questionnaires will be designed by the study team with input from the Professional Expert Panel (PEP) - see Patient and Public Involvement, below. Where possible, previously used and/or validated measures are being used. The study team will devise measures where none are available.

The baseline questionnaire will ask about participants' current experiences and attitudes, as well as collecting some retrospective data about participants' experiences and attitudes at the start of the pandemic and/or pre-pandemic. Follow-up questionnaires will repeat outcome measures, and may include new items relevant to the progression of the pandemic. The data dictionary (https://www.uk-reach.org/data-dictionary) contains the source(s) for each question. The baseline questionnaire is included as an Appendix.

Patient and public involvement

The UK-REACH team have worked closely with national and local organisations representing ethnic minority HCWs, who have been closely involved in identifying the research questions, deciding the study methodology, and are included either as members of the study delivery team or collaborators. They are also part of the Stakeholder group (see Supplementary Information for list of organisations) that will meet monthly to monitor study progress and research outputs, and provide advice to the research team on study delivery and progress. This group will also be central in disseminating the research findings. Alongside the high-level national organisation input into this stakeholder group, we will have representation from ethnic minority HCWs, including those who have contracted COVID-19 (mild to severe), to provide feedback on our work and how it should be disseminated. Members of our stakeholder/public engagement group will also sit on the Scientific Committee to ensure there is a seamless flow of information from the research team and the public engagement/stakeholder group. In addition we are working closely with the Centre for BME Health (Leicester, UK) to ensure that we are working to engage staff from a range of ethnic groups.

A Professional Expert Panel (PEP) will provide feedback on UK-REACH study materials and sampling methodology such as surveys, questionnaires, and interview and focus group topic guides. The PEP is made up of individuals who work within a healthcare setting from a range of ethnic backgrounds, occupational backgrounds, and genders. Staff have unique insight related to their professions or ethnic groups and are, therefore, in a position to provide advice and lived experience related to certain aspects of the project. The aim is for the PEP to draw on their experiences when providing their comments to ensure research instruments are at optimum suitability for study participants. The PEP meets virtually on a bi-monthly basis via Microsoft Teams. Study items/documents for discussion are circulated a week in advance of the PEP meeting and the group's Chair and Co-chair (PEP members who both volunteered to take on the role) moderate the meeting. UK-REACH team members are present in order to answer study-specific queries, and so only enter the discussions to

For peer review only - http://bmjopen.bmj.com/site/about/guidelines.xhtml

do so. The PEP also interacts with the study team between meetings via email with any additional feedback.

Primary outcome measures

Clinical COVID-19 outcomes

Participants will be asked to self-report COVID-19 infection, defined as either a positive SARS-CoV-2 polymerase chain reaction (PCR) or antibody test, or as self-reported suspected infection; the latter will be particularly relevant for those reporting historic illness early in the pandemic before widespread availability of testing. In our analyses we will consider all those with a PCR assay for SARS-CoV-2 or a positive anti-SARS-CoV-2 serology assay as being infected. To ensure those that acquired infection prior to widespread testing availability are not excluded, in those who have never been tested by PCR or serology, we will determine infection status based on whether they, or another healthcare professional, suspected them of having had COVID-19. To investigate how the inclusion of those that report suspected (but not confirmed) COVID-19 impacts upon our results, we will conduct sensitivity analyses examining only those who have undergone laboratory testing for current/previous infection.

Those reporting COVID-19 illness will be asked about: date of onset, the nature of symptoms experienced and their duration, and hospitalisation (including any time spent in intensive care). Corroboration of the details of these outcomes will be possible using linked electronic healthcare records (see “Measures obtained via data linkage”, below).

General health

This will be measured using the EQ-5D-5L instrument(23) (https://euroqol.org/), which contains five dimensions on mobility, self-care, usual activities, pain and discomfort, and anxiety and depression, plus an overall self-report of health.

Mental health

This will be measured using the Patient Health Questionnaire-2 (PHQ-2)(24) for depression, the Generalised Anxiety Disorder-2 (GAD-2)(25) scale for anxiety, a three-item abbreviated version of the PCL-6 scale for post-traumatic stress disorder,(26) a three-item abbreviated version of the UCLA Loneliness scale, (27) and an Office for National Statistics question about overall life satisfaction.(28) Participants will also be asked key questions from the Utrecht Work Engagement Scale (29) and the GMC National Training Survey questions on burnout(30) (from the Copenhagen Burnout Inventory (31)).

Questionnaire measures

Ethnicity

In this study we will ask participants to self-identify the ethnic group with which they most identify using the 18 UK Census 2011 Categories.(32) The questionnaire also asks the ethnic group of any partner and of parents.

We will then collapse these 18 categories into five main ethnic categories also defined within the Census (Black, Asian, Mixed, Other, White). We will further collapse them into two groups which we will refer to as “white” (White British, White Irish, White Gypsy or Traveller, White Other) and “ethnic minority” (all other ethnic groups). There is currently considerable debate about the categorisation of people using ethnic groups, and in particular, the grouping of people who do not

For peer review only - http://bmjopen.bmj.com/site/about/guidelines.xhtml

identify as white into a single category. There is also considerable debate and controversy about the words used to describe such a broad and heterogeneous grouping, with terms such as "people of colour", "Black Asian and Minority Ethnic" or "BAME" used. In our choice of terms we have followed the BMJ who in their special edition on Racism in Medicine use the term "ethnic minority" as one that is most likely to be understood by our study population.(33) We fully acknowledge that broad ethnic groupings can mask important ethnic and cultural differences, and where possible we will use more refined ethnic groupings, while also acknowledging the heterogeneity within them.

Nationality, religion, and languages

Country of birth, nationality, parents' country of birth, grandparents' country of birth (born in UK / not born in UK), age learned English (if second language), language(s) spoken at home (currently and as a child), religion, religiosity, ethnic identity.

Other demographics and education

Age, gender, sex, marital status, highest level of education completed, year and country of primary professional qualification (if applicable), highest level of education achieved by parents.

Work

Job role(s), sector(s), grade and specialty (for doctors), NHS band (for other HCWs), registered field of practice (nurses); work location(s); whether currently working, reasons for not working (if applicable); hours worked in a typical week; frequency of night shifts; contact with patients (with and without COVID-19), colleagues and others (remotely, face-to-face with social distancing, with physical contact); time spent travelling to and from work, modes of transport; access to, use of, and training to use personal protective equipment (PPE); exposure to aerosol-generating procedures; NHS COVID-19 risk assessment experiences; feelings about raising a clinical concern at work, perceptions of fairness of organisation with regards career progression; redeployment as a result of the pandemic, patient exposure, training and supervision in redeployed role (if applicable); proportion of colleagues of same ethnicity to self, proportion of colleagues who are white; work engagement.

Home and social life

Household composition (numbers, ages, relationship to participant) and sharing of accommodation; number of household members travelling using public transport or in jobs that bring them into close contact with others; childcare and support "bubbles"; length of time at current address; type and size of accommodation including amount of shared space and access to safe outdoor space; numbers of social contacts (remotely, face to face with social distancing, with physical contact), proportion of friends of the same ethnic group to self.

Harassment and discrimination

Experiences of discrimination in day-to-day life; discrimination at work and whether made a complaint (if applicable).

Physical health, mental health, and wellbeing

Height, weight, smoking and alcohol use, physical activity at work, general physical activity, change in lifestyle since start of pandemic, healthcare experience in 2019 (GP consultations and hospital admittance), flu vaccine uptake, medication, health conditions and pregnancy, quality of life, general anxiety, depression, post-traumatic stress disorder (PTSD), loneliness, and general life satisfaction.

For peer review only - http://bmjopen.bmj.com/site/about/guidelines.xhtml

COVID-19 experiences and beliefs

COVID-19 exposure, testing and test positivity; symptoms experienced, plus their severity and longevity, including diagnosis of long-COVID (if applicable); behaviour changes due to COVID-19; concern, knowledge and beliefs about COVID-19; COVID-19 information sources; enjoyment of first national lockdown (Spring 2020), COVID-19 vaccine trial participation; COVID-19 vaccination intention including offers, uptake (including vaccine brand), or reasons for refusal, and vaccine beliefs.

Trait and state psychological measures

'Big five' personality traits, locus of control, health locus of control, risk taking, burnout, personal need for structure.

Open-ended questions

The baseline questionnaire will include three open-ended free-text questions: "What are your thoughts on why people from ethnic minorities working in health and care have been more severely affected by COVID-19?", "How do you see society changing as a result of COVID-19?", "How do you see your own future changing as a result of COVID-19?".

Evaluation questions

Views on the length of the questionnaire and on its usefulness for understanding ethnicity and COVID-19.

Measures obtained via data linkage

Data linkage will be used to corroborate COVID-19 clinical outcomes (acute infection, antibody positivity), major comorbidities and patterns of healthcare use.

Biological sampling

At baseline we will also seek consent to re-contact participants in the future for DNA sampling and sampling related to immune profiling although we will submit an amendment to implement this sampling and detail the specifics relating to this at the time of submitting the amendment.

Statistical analysis

Descriptive statistics will be calculated for the primary outcome measures, and for ethnicity and key confounder/explanatory variables.

Univariate associations between ethnicity and primary outcome measures, and between ethnicity and key confounders/explanatory variables calculated using chi-squared tests for categorical variables, and t-tests and analyses of variance for continuous measures, with non-parametric equivalents used as appropriate for ordinal variables. This will enable the examination of the behavioural, social, and clinical phenotypes of the cohort in relation to the patterns of demographics, job role, attitudes to work and work climate, social and living circumstances, values and personality, and physical and mental health by ethnicity.

Using baseline data, multivariable analyses will test for associations between ethnicity and key outcomes, adjusted for the confounder/explanatory variables found to have a statistically significant univariate association with either ethnicity or the primary outcome variable(s), with interactions

For peer review only - http://bmjopen.bmj.com/site/about/guidelines.xhtml

For peer review only

included as appropriate. Using follow-up data, mixed models will be used to model changes over time by ethnic group.

Models will fit ethnicity as both a binary indicator (ethnic minority versus white) and as a categorical variable based on ONS categorisation of ethnicity, with the white group used as the reference group.

ETHICS AND DISSEMINATION

Ethical approval

The study has been approved by the Health Research Authority (Brighton and Sussex Research Ethics Committee; ethics reference: 20/HRA/4718).

Ethical considerations

Whilst this study is low risk, the questionnaire covers sensitive topics around COVID-19, ethnicity (including issues of discrimination and harassment), and mental health, and these could be distressing to participants. We aim to manage this risk by clearly indicating on the PIS that the questionnaire covers sensitive topics and that participants are under no obligation to answer these, or indeed any other, questions, and provide links to support organisations.

Participant confidentiality

The participants will be identified only by a unique identifier in the main research database. Identifiable information (name, date of birth, address etc) will be stored in a separate secure database, and will be accessed only by a small number of authorised staff at the University of Leicester who require access to administer the study. All documents will be stored securely, and only be accessible by study staff and authorised personnel. The study will comply with the Data Protection Act, which requires data to be anonymised as soon as it is practical to do so. Any dissemination of study findings will follow best-practice guidelines for deductive disclosure. Only aggregate data will included in publications.

Discontinuation/withdrawal of participants from study

Participants who wish to withdraw from the cohort study will be asked to determine the desired level of withdrawal from the study as described by the two options below. We will keep a record of consent for all participants to manage re-contact and for future audit. We will accept signed withdrawal forms from participants or, if they are unable to complete a withdrawal form themselves, from someone acting on the participant's behalf. At the present time, withdrawal forms will be completed electronically, but signed written forms will also be accepted when it is feasible to securely receive and store these. The options that participants will be given if they wish to withdraw:

Option 1 - No further contact: we would no longer contact the participant, but would have the participant's permission to continue to obtain information by accessing their health records in the future.

Option 2 – No further contact or access: we would no longer contact the participant or obtain information from the participant's health records in the future.

If participants withdraw from UK-REACH, then any data and samples already collected will remain and be used in the study. Information and data will continue to be collected about participants'

For peer review only - http://bmjopen.bmj.com/site/about/guidelines.xhtml

health from central NHS records, hospital records, and participants' GPs, unless participants state otherwise on the withdrawal form.

Description of data flow

See Figure 3 for a description of the data flow.

Dissemination plan

Quarterly reports in months 3, 6, 9, and 12 summarising recruitment progress and initial findings on relationship between ethnicity, COVID-19 diagnosis and outcomes, physical/mental well-being, and professional and social factors. Brief reports will be produced and submitted for review by the stakeholder group (see Supplementary material for details), PEP, Study Steering Committee and the UK Government's Scientific Advisory Group for Emergencies (SAGE). Papers submitted to peer reviewed journals and pre-print servers.

DATA SHARING STATEMENT

To access data or samples produced by the UK-REACH study, the working group representative must first submit a data and material request form to the **Data Access Committee** (DAC) providing details for all manuscript proposals. The DAC will establish priorities for core and ancillary projects. For ancillary studies outside of the core deliverables, the **Steering Committee** will make final decisions once they have been approved by the **Core Management Group** and the DAC. Decisions on granting the access to data/materials will be made within eight weeks. Third party requests from outside the Project will require explicit approval of the Steering Committee once approved by the Core Management Group and the DAC.

AUTHORS CONTRIBUTIONS

MP conceived of the idea and led the application for funding with input from MT, KK, ICM, KW, RF, LN, SC, KRA, LG, AG, LVW and CJ. The survey was designed by KW, MP, ICM, CMel, CJ, ALG, AG, LN, CAM and RF. Online consent and survey tools were developed by LB with support from CG and VM. KW wrote the first draft of the manuscript with input from MP and all co-authors. All authors approved the submitted manuscript.

ACKNOWLEDGEMENTS

We wish to acknowledge the PEP group and the STAG group (see Supplementary material), and SERCO, as well as the following people for their support in organising the email invites from the regulatory bodies: Kerrin Clapton and Andrew Ledgard (General Medical Council), Caroline Kenny (Nursing and Midwifery Council), David Teeman and Lisa Bainbridge (General Dental Council), My Phan and John Tse (General Pharmaceutical Council), Angharad Jones (General Optical Council), Katherine Timms and Charlotte Rogers (The Health and Care Professions Council) and Mark Neale (Pharmaceutical Society of Northern Ireland).

FUNDING STATEMENT

UK-REACH is supported by a grant from the MRC-UK Research and Innovation (MR/V027549/1) and the Department of Health and Social Care through the National Institute for Health Research (NIHR) rapid response panel to tackle COVID-19. Core funding was also provided by NIHR Biomedical

For peer review only - http://bmjopen.bmj.com/site/about/guidelines.xhtml

Research Centres. This work is carried out with the support of BREATHE -The Health Data Research Hub for Respiratory Health [UKRI MC_PC_19004] in partnership with SAIL Databank. BREATHE is funded through the UK Research and Innovation Industrial Strategy Challenge Fund and delivered through Health Data Research UK.

ALG was funded by internal fellowships at the University of Leicester from the Wellcome Trust Institutional Strategic Support Fund (204801/Z/16/Z) and the BHF Accelerator Award (AA/18/3/34220). CJ holds a Medical Research Council Clinical Research Training Fellowship (MR/P00167X/1). KA is supported by Health Data Research (HDR) UK and as a NIHR Senior Investigator Emeritus (NF-SI-0512-10159). KK and LJG are supported by the National Institute for Health Research (NIHR) Applied Research Collaboration East Midlands (ARC EM). KK, MP, and RCF are supported by the NIHR Leicester Biomedical Research Centre (BRC). CAM is an NIHR Academic Clinical Fellow (ACF-2018-11-004). LBN is supported by an Academy of Medical Sciences Springboard Award (SBF005\1047). MP is supported by a NIHR Development and Skills Enhancement Award. MDT holds a Wellcome Trust Investigator Award (WT 202849/Z/16/Z) and an NIHR Senior Investigator Award. KW holds an NIHR Career Development Fellowship (CDF: CDF-2017-10-008). LVW holds a GSK/British Lung Foundation Chair in Respiratory Research (C17-1).

The views expressed in the publication are those of the author(s) and not necessarily those of the National Health Service (NHS), the NIHR or the Department of Health and Social Care.

This research was funded in whole, or in part, by the Wellcome Trust [WT204801/Z/16/Z and WT 202849/Z/16/Z]. For the purpose of open access, the author has applied a CC BY public copyright licence to any Author Accepted Manuscript version arising from this submission.

COMPETING INTERESTS STATEMENT

SC is Deputy Medical Director of the General Medical Council, UK Honorary Professor, University of Leicester. KK is Director of the University of Leicester Centre for Black Minority Ethnic Health, Trustee of the South Asian Health Foundation, Chair of the Ethnicity Subgroup of SAGE and Member of Independent SAGE. LVW receives grant funding from GSK and Orion, outside of the submitted work. KA has served as a paid consultant, providing unrelated methodological and strategic advice, to the pharmaceutical and life sciences industry generally and has received unrelated research funding from Association of the British Pharmaceutical Industry, European Federation of Pharmaceutical Industries & Associations, Pfizer, Sanofi and Swiss Precision Diagnostics. He is a Partner and Director of Visible Analytics Limited, a healthcare consultancy company.

WORD COUNT

4087

REFERENCES

1. Office for National Statistics. Coronavirus (COVID-19) related deaths by occupation, England and Wales: deaths registered between 9 March and 28 December 2020 2021 [Available from: https://www.ons.gov.uk/peoplepopulationandcommunity/healthandsocialcare/causesofdeath/bulletins/coronaviruscovid19relateddeathsbyoccupationenglandandwales/deathsregisteredbetween9marchand28december2020#deaths-involving-covid-19-among-men-and-women-health-and-social-care-workers.

For peer review only - http://bmjopen.bmj.com/site/about/guidelines.xhtml

2. Mutambudzi M, Niedwiedz C, Macdonald EB, Leyland A, Mair F, Anderson J, et al. Occupation and risk of severe COVID-19: prospective cohort study of 120 075 UK Biobank participants. Occupational and Environmental Medicine. 2020:oemed-2020-106731.

3. Levene LS, Coles B, Davies MJ, Hanif W, Zaccardi F, Khunti K. COVID-19 cumulative mortality rates for frontline healthcare staff in England. British Journal of General Practice. 2020;70(696):327-8.

4. Ayoubkhani D, Khunti K, Nafilyan V, Maddox T, Humberstone B, Diamond SI, et al. Epidemiology of post-COVID syndrome following hospitalisation with coronavirus: a retrospective cohort study. medRxiv. 2021:2021.01.15.21249885.

5. Khanijahani A. Racial, ethnic, and socioeconomic disparities in confirmed COVID-19 cases and deaths in the United States: a county-level analysis as of November 2020. Ethnicity & Health. 2020:1-14.

6. Khunti K, Singh AK, Pareek M, Hanif W. Is ethnicity linked to incidence or outcomes of covid-19? BMJ. 2020;369:m1548.

7. Kirby T. Evidence mounts on the disproportionate effect of COVID-19 on ethnic minorities. The Lancet Respiratory Medicine. 2020;8(6):547-8.

8. Platt L, Warwick R. COVID-19 and Ethnic Inequalities in England and Wales*. Fiscal Studies. 2020;41(2):259-89.

9. Webb Hooper M, Nápoles AM, Pérez-Stable EJ. COVID-19 and Racial/Ethnic Disparities. JAMA. 2020;323(24):2466-7.

10. Nazroo J, Murray K, Taylor H, Bécares L, Field Y, Kapadia D, et al. Rapid Evidence Review: Inequalities in relation to COVID-19 and their effects on London. University of Manchester, The Ubele Initiative, University of Sussex; 2020.

11. Sze S, Pan D, Nevill CR, Gray LJ, Martin CA, Nazareth J, et al. Ethnicity and clinical outcomes in COVID-19: A systematic review and meta-analysis. EClinicalMedicine. 2020;29-30:100630.

12. Pan D, Sze S, Minhas JS, Bangash MN, Pareek N, Divall P, et al. The impact of ethnicity on clinical outcomes in COVID-19: A systematic review. EClinicalMedicine. 2020;23.

13. Clift AK, Coupland CAC, Keogh RH, Diaz-Ordaz K, Williamson E, Harrison EM, et al. Living risk prediction algorithm (QCOVID) for risk of hospital admission and mortality from coronavirus 19 in adults: national derivation and validation cohort study. BMJ. 2020;371:m3731.

14. Sahebi A, Nejati B, Moayedi S, Yousefi K, Torres M, Golitaleb M. The prevalence of anxiety and depression among healthcare workers during the COVID-19 pandemic: An umbrella review of meta-analyses. Progress in Neuro-Psychopharmacology and Biological Psychiatry. 2021:110247.

For peer review only - http://bmjopen.bmj.com/site/about/guidelines.xhtml

15. Galli F, Pozzi G, Ruggiero F, Mameli F, Cavicchioli M, Barbieri S, et al. A Systematic Review and Provisional Metanalysis on Psychopathologic Burden on Health Care Workers of Coronavirus Outbreaks. Frontiers in Psychiatry. 2020;11(1001).

16. Greene T, Harju-Seppänen J, Adeniji M, Steel C, Grey N, Brewin CR, et al. Predictors and rates of PTSD, depression and anxiety in UK frontline health and social care workers during COVID-19. medRxiv. 2020:2020.10.21.20216804.

17. Iob E, Frank P, Steptoe A, Fancourt D. Levels of Severity of Depressive Symptoms Among At-Risk Groups in the UK During the COVID-19 Pandemic. JAMA Network Open. 2020;3(10):e2026064-e.

18. Iob E, Steptoe A, Fancourt D. Abuse, self-harm and suicidal ideation in the UK during the COVID-19 pandemic. The British Journal of Psychiatry. 2020;217(4):543-6.

19. Wanigasooriya K, Palimar P, Naumann DN, Ismail K, Fellows JL, Logan P, et al. Mental health symptoms in a cohort of hospital healthcare workers following the first peak of the COVID-19 pandemic in the UK. BJPsych Open. 2020;7(1):e24.

20. Trivedy C, Mills I, Dhanoya O. The impact of the risk of COVID-19 on Black, Asian and Minority Ethnic (BAME) members of the UK dental profession. British Dental Journal. 2020;228(12):919-22.

21. Martin CA, Patel P, Goss C, Jenkins DR, Price A, Barton L, et al. Demographic and occupational determinants of anti-SARS-CoV-2 IgG seropositivity in hospital staff. Journal of Public Health. 2020.

22. Drew DA, Nguyen LH, Steves CJ, Menni C, Freydin M, Varsavsky T, et al. Rapid implementation of mobile technology for real-time epidemiology of COVID-19. Science. 2020;368(6497):1362-7.

23. Herdman M, Gudex C, Lloyd A, Janssen MF, Kind P, Parkin D, et al. Development and preliminary testing of the new five-level version of EQ-5D (EQ-5D-5L). Quality of Life Research. 2011;20(10):1727-36.

24. Löwe B, Kroenke K, Gräfe K. Detecting and monitoring depression with a two-item questionnaire (PHQ-2). Journal of Psychosomatic Research. 2005;58(2):163-71.

25. Plummer F, Manea L, Trepel D, McMillan D. Screening for anxiety disorders with the GAD-7 and GAD-2: a systematic review and diagnostic metaanalysis. General Hospital Psychiatry. 2016;39:24-31.

26. Lang AJ, Wilkins K, Roy-Byrne PP, Golinelli D, Chavira D, Sherbourne C, et al. Abbreviated PTSD Checklist (PCL) as a guide to clinical response. General hospital psychiatry. 2012;34(4):332-8.

27. Hughes ME, Waite LJ, Hawkley LC, Cacioppo JT. A Short Scale for Measuring Loneliness in Large Surveys:Results From Two Population-Based Studies. Research on Aging. 2004;26(6):655-72.

For peer review only - http://bmjopen.bmj.com/site/about/guidelines.xhtml

For peer review only

28. Surveys using our four personal well-being questions: A guide to what surveys include the four ONS personal well-being questions: Office for National Statistics; 2018 [Available from: https://www.ons.gov.uk/peoplepopulationandcommunity/wellbeing/methodologies/surveysusingthe4officefornationalstatisticspersonalwellbeingquestions.

29. Schaufeli WB, Bakker AB, Salanova M. The Measurement of Work Engagement With a Short Questionnaire:A Cross-National Study. Educational and Psychological Measurement. 2006;66(4):701-16.

30. General Medical Council National training surveys 2019: Initial findings report. General Medical Council, 2019.

31. Kristensen TS, Borritz M, Villadsen E, Christensen KB. The Copenhagen Burnout Inventory: A new tool for the assessment of burnout. Work & Stress. 2005;19(3):192-207.

32. Harmonised Concepts and Questions for Social Data Sources, Primary Principles: Ethnic Group. Government Statistical Service; 2016.

33. Ladher N. Let's talk about race: BMJ; 2020 [Available from: https://blogs.bmj.com/bmj/2020/02/13/navjoyt-ladher-lets-talk-about-race/.

For peer review only

Figure captions

Figure 1: Study flowchart

Figure 2: UK-REACH Work package 2 timeline as of 4th February 2021. Dates are shown across the bottom from October 2019 to July 2021. The COVID-19 outbreak began in the UK in January 2020 with numbers of daily hospital admissions due to COVID-19 shown by the lower orange line for Wave one and Wave two. Vaccinations began in December 2020 and are shown by the green line for daily vaccinations. Lockdowns are shown by horizontal red bars, the first national lockdown beginning on March 23rd 2020, the second on November 5th, and the third of January 5th 2021. Lockdowns differed somewhat in timing between England, Wales, Scotland and Northern Ireland. Tiered local restrictions were in place in various regions of the UK between lockdowns, shown in yellow. Questionnaire 1 of Work package 2 began to be distributed on December 4th onwards and is being distributed until the end of March 2021. Questionnaire 1 asks about current events and working conditions, as well as retrospectively about events and working conditions pre-Covid in 2019, about early Covid responses in the first months of 2021, and about events during the first national lockdown. Questionnaire 2 will be distributed four months after registration for questionnaire 1 and therefore will be distributed between April and June 2021. Questionnaire 2 asks primarily about current working conditions, and changes to other aspects of participants' lives captured in Questionnaire 1, including key measures of physical and mental health. With consent, the questionnaire data will be linked to electronic healthcare record data.

Figure 3: Data flow diagram. Solid lines with a circle indicate identifiable data flows for linkage purposes only. Solid lines with an arrow indicate anonymous outputs. Dashed lines indicate de-identified data. After consenting to join UK-REACH, participants provide limited personal identifiable information (PII) which are stored in a secure location at the University of Leicester, alongside study ID. Questionnaire data (including study ID but not alongside the aforementioned PII) are stored in a different secure location at the University of Leicester. Participants provide consent for the study to follow their health by extracting information from electronic health records. Relevant PII for each participant will be securely transferred to NHS Wales Informatics Service (alongside the UK-REACH study ID) in order to acquire NHS number/Community Health Index (CHI) number (for Scotland)/Health and Care number (for Northern Ireland). NHS/CHI/H&C number will then be used to link to healthcare records by the relevant data provider across the UK. De-identified health records will be sent directly to the Secure Anonymised Information Linkage databank (SAIL, https://saildatabank.com), retaining Study ID but excluding PII. Questionnaire data (including study ID) will be sent from the University of Leicester to SAIL. These datasets will be linked within SAIL to provide the complete dataset. Interim analysis of unlinked questionnaire data will be performed at the University of Leicester using pseudonymised datasets. All linked data analysis will take place within SAIL. The above diagram and style was interpreted by Anna Guyatt and Chris Orton from an initial data flow diagram created and provided by Andy Boyd at the University of Bristol. It has been repurposed and amended to illustrate data flows specific to the UK-REACH project.

For peer review only - http://bmjopen.bmj.com/site/about/guidelines.xhtml

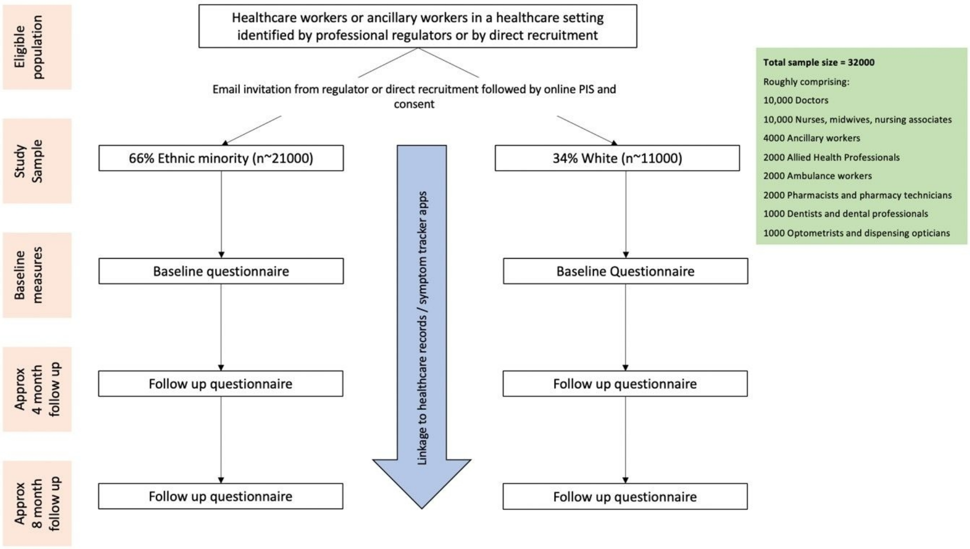

Figure 1: Study flowchart

116x65mm (300 x 300 DPI)

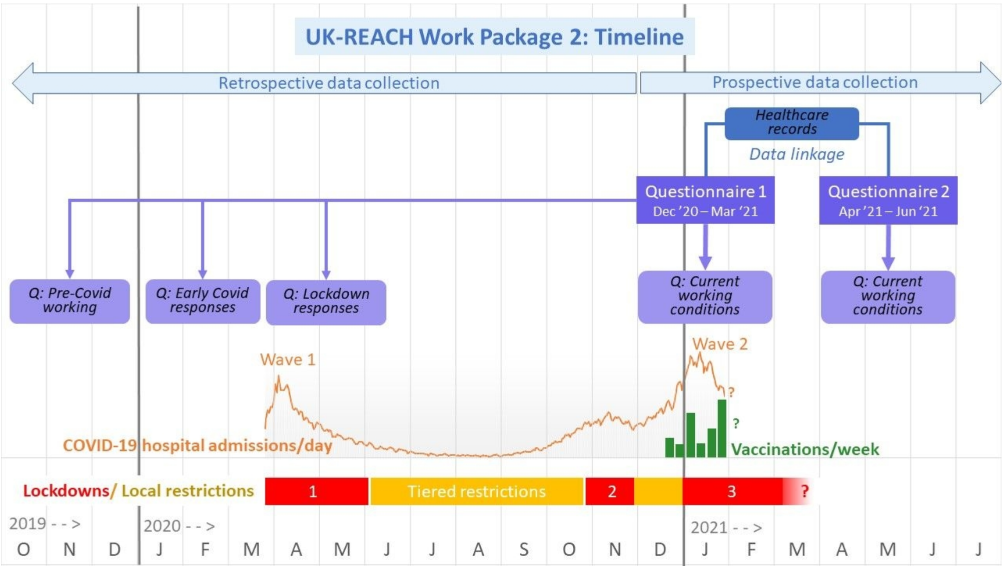

Figure 2: UK-REACH Work package 2 timeline as of 4th February 2021. Dates are shown across the bottom from October 2019 to July 2021. The COVID-19 outbreak began in the UK in January 2020 with numbers of daily hospital admissions due to COVID-19 shown by the lower orange line for Wave one and Wave two. Vaccinations began in December 2020 and are shown by the green line for daily vaccinations. Lockdowns are shown by horizontal red bars, the first national lockdown beginning on March 23rd 2020, the second on November 5th, and the third of January 5th 2021. Lockdowns differed somewhat in timing between England, Wales, Scotland and Northern Ireland. Tiered local restrictions were in place in various regions of the UK between lockdowns, shown in yellow. Questionnaire 1 of Work package 2 began to be distributed on December 4th onwards and is being distributed until the end of March 2021. Questionnaire 1 asks about current events and working conditions, as well as retrospectively about events and working conditions pre-Covid in 2019, about early Covid responses in the first months of 2021, and about events during the first national lockdown. Questionnaire 2 will be distributed four months after registration for questionnaire 1 and therefore will be distributed between April and June 2021. Questionnaire 2 asks primarily about current working conditions, and changes to other aspects of participants' lives captured in Questionnaire 1, including key measures of physical and mental health. With consent, the questionnaire data will be linked to electronic healthcare record data.

108x60mm (300 x 300 DPI)

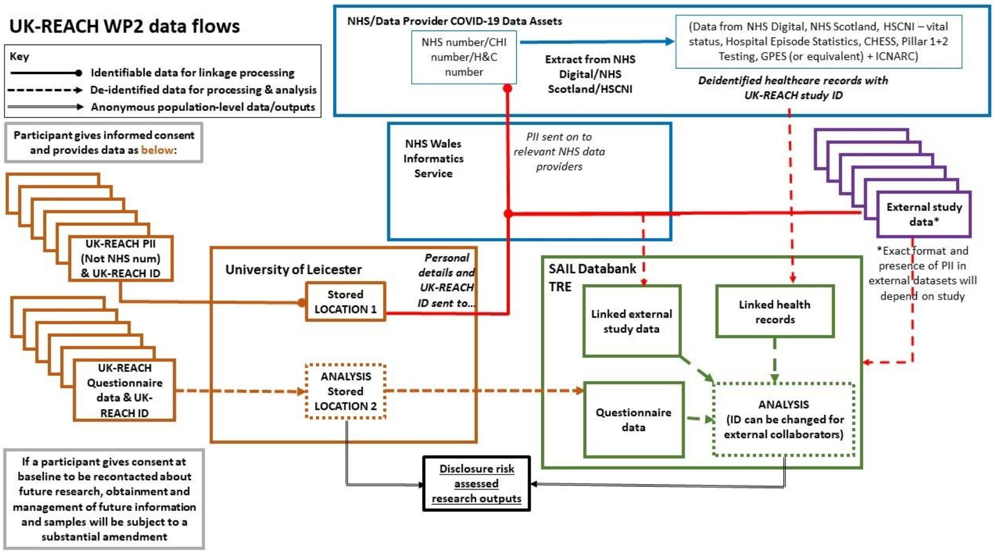

Figure 3: Data flow diagram. Solid lines with a circle indicate identifiable data flows for linkage purposes only. Solid lines with an arrow indicate anonymous outputs. Dashed lines indicate de-identified data. After consenting to join UK-REACH, participants provide limited personal identifiable information (PII) which are stored in a secure location at the University of Leicester, alongside study ID. Questionnaire data (including study ID but not alongside the aforementioned PII) are stored in a different secure location at the University of Leicester. Participants provide consent for the study to follow their health by extracting information from electronic health records. Relevant PII for each participant will be securely transferred to NHS Wales Informatics Service (alongside the UK-REACH study ID) in order to acquire NHS number/Community Health Index (CHI) number (for Scotland)/Health and Care number (for Northern Ireland). NHS/CHI/H&C number will then be used to link to healthcare records by the relevant data provider across the UK. De-identified health records will be sent directly to the Secure Anonymised Information Linkage databank (SAIL, https://saildatabank.com), retaining Study ID but excluding PII. Questionnaire data (including study ID) will be sent from the University of Leicester to SAIL. These datasets will be linked within SAIL to provide the complete dataset. Interim analysis of unlinked questionnaire data will be performed at the University of Leicester using pseudonymised datasets. All linked data analysis will take place within SAIL. The above diagram and style was interpreted by Anna Guyatt and Chris Orton from an initial data flow diagram created and provided by Andy Boyd at the University of Bristol. It has been repurposed and amended to illustrate data flows specific to the UK-REACH project.

108x60mm (300 x 300 DPI)

SUPPLEMENTARY INFORMATION

List of Members of UK-REACH Stakeholder Group (STAG) as at January 2021

1. General Medical Council (GMC)
2. Nursing and Midwifery Council (NMC)
3. General Dental Council (GDC)
4. General Pharmaceutical Council (GPC)
5. Royal College of Psychiatrists (RCPsych)
6. Royal College of Obstetricians and Gynaecologists (RCOG)
7. Royal College of Midwives (RCM)
8. NHS Confederation
9. British Association of Physicians of Indian Origin (BAPIO)
10. Sudan Doctors' Union –UK Branch
11. Association of Pakistani Physicians of Northern Europe (APPNE)
12. South Asian Health Foundation (SAHF)
13. Health Education England (HEE)
14. General Optical Council (GOC)
15. Filipino Nurses Association UK (FNAUK)
16. Pharmaceutical Society of Northern Ireland (PSNI)
17. Health and Care Professions Council (HCPC)
18. NHS England & Improvement
19. British Medical Association (BMA)
20. Medical Association of Nigerians Across Great Britain (MANSAG)

List of Members of UK-REACH Professional Expert Panel (PEP) as at January 2021

Susie Lagrata (co-Chair), Nurse.
Padmasayee Papineni (co-Chair), Doctor.
Sandra Kazembe, Nurse.
Tatiana Monteiro, Domestic worker.
Juliette Mutumiyana, Doctor.
Satheesh Mathew, Doctor.
Amir Burney, Doctor.
Ahmed Hashim, Doctor.
Tiffanie Harrison, Nurse.

For peer review only

UK-REACH Questionnaire

Introduction

token ________________________________

Thank you for taking part in the UK-REACH study on understanding Covid-19 outcomes for ethnic minority healthcare workers (https://uk-reach.org/).
The study is interested in healthcare workers from all backgrounds, and particularly in healthcare workers from ethnic minorities.
This questionnaire asks about your work and about your ethnic and cultural background. It also asks questions that will help the research team understand how work fits into your life more broadly, and how work affects your life.
These include questions about:

• your age and other background information;
• your work in healthcare;
• your ethnicity, culture, religion, languages and education;
• your home environment and living circumstances;
• your health, both physical and mental;
• your attitudes and values in life and at work;
• your occupation and working life;
• your possible exposure to COVID-19;
• your experience of events before and during the UK national lockdown

Some questions may seem unusual and it will not always seem obvious why they are being asked. They are however important, and it would be helpful if you could answer as many as possible.

Some of the questions ask about sensitive topics, and if you cannot or do not wish to answer them then simply click on “Prefer not to answer”. Please be reassured that answers will be treated in accordance with strict research governance procedures, and the study has been reviewed by the Brighton and Sussex Research Ethics Committee.

Most people should be able to answer this questionnaire in about 30 minutes or so. Your answers will be stored as you go along, so you can pause the questionnaire and resume it later if you want. To do this, please select Save & Return Later. You can then continue the questionnaire from where you left off by selecting the Resume button in your UK REACH profile. You can return to your profile using the Return to Profile button provided.

If you need any further information about the study, or you have problems with any part of it, then the study team can be reached via email at uk-reach@leicester.ac.uk or by telephone on 07425611865.
If you are affected by any of the issues raised in this questionnaire or are looking for information on COVID-19 (coronavirus) please visit:
• Government guidelines: www.gov.uk/coronavirus
• NHS advice: https://www.nhs.uk/conditions/coronavirus-covid-19/symptoms/
• NHS – Where to get urgent help for mental health:
https://www.nhs.uk/using-the-nhs/nhs-services/mental-health-services/where-to-get-urgent-help-for-mental-health/
• Covid-19 Workforce Wellbeing: https://www.practitionerhealth.nhs.uk/covid-19-workforce-wellbeing
• Victim Support – support for people affected by crime or traumatic events, including hate crime:
https://www.victimsupport.org.uk/
• Samaritans – Emotional support for everyone: www.samaritans.org
• Mind – Advice and support for anyone with a mental health problem: www.mind.org.uk

UK-REACH ID
This has been autocompleted. This ID is used for research purposes only. ________________________________

Your UK-REACH ID is Empty!
Please return to your user profile and use the link provided there to access the questionnaire.

Return to User Profile

For peer review only

Introduction

If you wish to go back to an earlier question, please use the Previous Page button at the bottom of each page. Please do not use the back button on your browser as that will mean that you leave the questionnaire.

For peer review only

Section 1/10: Background information

0% complete

.

These questions ask for some simple information about you.

There will be a separate section on your ethnic and cultural background later in the questionnaire.

For peer review only

Section 1/10: Background information

0% complete
.

Question	Response
Which of the following best describes you?	◯ Man<br>◯ Woman<br>◯ I use another term to describe my gender<br>◯ Prefer not to answer
Please enter the term you use to describe your gender, or enter "Prefer not to answer".	__________________________
What was your sex assigned at birth?	◯ Male<br>◯ Female<br>◯ Other<br>◯ Prefer not to answer
Which of the following best describes your marital status?<br><br>You will be asked more about who you live with later in the questionnaire.	◯ Single (never married or in civil partnership)<br>◯ Cohabiting with a partner<br>◯ Married (including those in civil partnerships)<br>◯ Divorced (including formerly in a civil partnership which is now legally dissolved)<br>◯ Separated (but still legally married or in a civil partnership)<br>◯ Widowed (including surviving partner from a civil partnership)<br>◯ Prefer not to answer

For peer review only

Section 2/10: Your Job

1% complete

·

There are many different types of healthcare jobs, and the study needs to know some things about your job.

Some people have more than one job or several roles within their job. These questions are principally about your main role/job. This is usually the role/job you spend most time in per week. If you have roles/jobs in both clinical and non-clinical settings, please think about the main role/job you have in a clinical setting, even if it is not the job you spend most time in each week.

You will also be asked about personal protective equipment (PPE).

For peer review only

Section 2/10: Your job

1% complete

.

For peer review only

What is your main job/role? Please choose the best fit and specify further if you wish.

If you are not currently working, please answer about your most recent role.

- ◯ Allied Health Professional - Arts therapist
- ◯ Allied Health Professional - Biomedical scientist
- ◯ Allied Health Professional - Chiropodist/Podiatrist
- ◯ Allied Health Professional - Clinical scientist
- ◯ Allied Health Professional - Dietician
- ◯ Allied Health Professional - Hearing aid dispenser
- ◯ Allied Health Professional - Occupational therapist
- ◯ Allied Health Professional - Operating department practicioner
- ◯ Allied Health Professional - Orthoptist
- ◯ Allied Health Professional - Physiotherapist
- ◯ Allied Health Professional - Practitioner psychologist
- ◯ Allied Health Professional - Prosthetist / Orthotist
- ◯ Allied Health Professional - Radiographer
- ◯ Allied Health Professional - Speech and language therapist
- ◯ Allied Health Professional - Other Allied Health Professional role (please specify)
- ◯ Ambulance - Emergency medical technician
- ◯ Ambulance - Paramedic
- ◯ Ambulance - Other ambulance role (please specify)
- ◯ Clinical support staff - OT Support Worker
- ◯ Clinical support staff - Phlebotomist
- ◯ Clinical support staff - Physiotherapy Assistant
- ◯ Clinical support staff - Radiography Assistant
- ◯ Clinical support staff - Other clinical support role (please specify)
- ◯ Dental - Clinical dental technician
- ◯ Dental - Dental Hygienist
- ◯ Dental - Dental nurse
- ◯ Dental - Dental technician
- ◯ Dental - Dentist
- ◯ Dental - Other dental role (please specify)
- ◯ Doctors - Doctor
- ◯ Medical associates - Advanced Critical Care Practitioner
- ◯ Medical associates - Anaesthesia associate
- ◯ Medical associates - Physician Associate
- ◯ Medical associates - Surgical Care Practitioner
- ◯ Medical associates - Other medical associate (please specify)
- ◯ Nursing and midwifery - Advanced Nurse Practitioner
- ◯ Nursing and midwifery - Healthcare assistant
- ◯ Nursing and midwifery - Maternity support worker
- ◯ Nursing and midwifery - Midwife
- ◯ Nursing and midwifery - Nurse
- ◯ Nursing and midwifery - Nursing Associate
- ◯ Nursing and midwifery - Other nursing and midwifery role (please specify)
- ◯ Pharmacy - Pharmacist
- ◯ Pharmacy - Pharmacy technician
- ◯ Pharmacy - Other pharmacy role (please specify)
- ◯ Optical - Dispensing optician
- ◯ Optical - Optometrist
- ◯ Other Optical role (please specify)
- ◯ Wider healthcare role - Administration
- ◯ Wider healthcare role - Catering services
- ◯ Wider healthcare role - Domestic services
- ◯ Wider healthcare role - Estates services
- ◯ Wider healthcare role - Porter
- ◯ Wider healthcare role - Other (Please specify)
- ◯ Any other role (please specify)
- ◯ Prefer not to answer

For peer review only

Please specify your wider healthcare role: __________________________________

Please specify your Allied Health Professional role: __________________________________

Please specify your ambulance role: __________________________________

Please specify your clinical support staff role: __________________________________

Please specify your dental role: __________________________________

Please specify your medical associates role: __________________________________

Please specify your nursing and midwifery role: __________________________________

Please specify your pharmacy role: __________________________________

Please specify your optical role: __________________________________

Please specify your job role: __________________________________

Please tell us about your working status now, and in the first month after the start of the UK national lockdown on 23rd March 2020:

	Not working	Working	Prefer not to answer
Now	○	○	○
In the first month after the start of the UK national lockdown on 23rd March 2020	○	○	○

Please indicate the reason(s) you are not working now (Select all that apply):

By 'shielding' we mean taking extra steps to protect yourself, by minimising interactions between yourself and others because you are at high risk of severe illness from coronavirus (COVID-19).

By 'On furlough', we mean paid by your employer through the Job Support Scheme or Job Retention Scheme and not allowed to work.

- ☐ Shielding due to own health
- ☐ Shielding due to household member's health
- ☐ On furlough
- ☐ On sick leave
- ☐ On carer's leave
- ☐ Unemployed
- ☐ Retired
- ☐ Other (please specify)
- ☐ Prefer not to answer

Please specify why you are not currently working: __________________________________

For peer review only

For peer review only - http://bmjopen.bmj.com/site/about/guidelines.xhtml

Please indicate the reason(s) you were not working at the start of the UK national lockdown on 23rd March 2020 (select all that apply):

By 'shielding' we mean taking extra steps to protect yourself, by minimising interactions between yourself and others because you are at high risk of severe illness from coronavirus (COVID-19).

By 'On furlough', we mean paid by your employer through the Job Support Scheme or Job Retention Scheme and not allowed to work.

- ☐ Shielding due to own health
- ☐ Shielding due to household member's health
- ☐ On furlough
- ☐ On sick leave
- ☐ On carer's leave
- ☐ Unemployed
- ☐ Retired
- ☐ Other (please specify)
- ☐ Prefer not to answer

Please specify why you were not working at the start of the UK national lockdown on 23rd March 2020:

____

In which of the following sectors is your current main job/ role?

If not currently working, please answer for your most recent main job/role.

Select all that apply.

- ☐ NHS
- ☐ Other public sector (e.g. local or national government)
- ☐ Private sector
- ☐ Private facility temporarily used by the NHS
- ☐ University / higher education
- ☐ Prefer not to answer

It would be helpful to us to know where in the UK your main job/role is located. Please type the first part of the postcode (e.g. W1G, CF24, BT12 or EH16). If you can't remember the number at the end, just type the first letters (e.g. CF, BT or EH).

If not currently working, please answer for your most recent main job/role.

Please note: We cannot directly identify your place of work from the first part of the postcode, but it does give a good indication of geographical location, such that place of work may be inferred.

You may enter "Prefer not to answer" if you do not wish to provide this information.

____

In which of the following sectors was your main job/role in the first month after the start of the UK national lockdown on 23rd March 2020?

Select all that apply.

- ☐ NHS
- ☐ Other public sector (e.g. local or national government)
- ☐ Private sector
- ☐ Private facility temporarily used by the NHS
- ☐ University / higher education
- ☐ Prefer not to answer

For peer review only

What is your current or most recent grade?	◯ Doctor in training post - Foundation level<br>◯ Doctor in training post - Core level<br>◯ Doctor in training post - Specialty level<br>◯ Locally employed / trust doctor - Foundation level<br>◯ Locally employed / trust doctor - Core level<br>◯ Locally employed / trust doctor - Specialty level<br>◯ GP<br>◯ Consultant<br>◯ SAS<br>◯ Other (please specify)<br>◯ Prefer not to answer
Please specify your current or most recent grade:	__________________________________
What was your grade at the start of the UK national lockdown on 23rd March 2020?	◯ Doctor in training post - Foundation level<br>◯ Doctor in training post - Core level<br>◯ Doctor in training post - Specialty level<br>◯ Locally employed / trust doctor - Foundation level<br>◯ Locally employed / trust doctor - Core level<br>◯ Locally employed / trust doctor - Specialty level<br>◯ GP<br>◯ Consultant<br>◯ SAS<br>◯ Other (please specify)<br>◯ Prefer not to answer
Please specify your grade:	__________________________________

For peer review only

What is your current or most recent specialty?

- ◯ Acute internal medicine
- ◯ Allergy
- ◯ Anaesthetics
- ◯ Audio vestibular medicine
- ◯ Aviation and space medicine
- ◯ Cardio-thoracic surgery
- ◯ Cardiology
- ◯ Chemical pathology
- ◯ Child and adolescent psychiatry
- ◯ Child mental health
- ◯ Clinical genetics
- ◯ Clinical neurophysiology
- ◯ Clinical oncology
- ◯ Clinical pharmacology and therapeutics
- ◯ Clinical radiology
- ◯ Community child health
- ◯ Community sexual and reproductive health
- ◯ Congenital cardiac surgery
- ◯ Cytopathology
- ◯ Dermatology
- ◯ Diagnostic neuropathology
- ◯ Emergency medicine
- ◯ Endocrinology and diabetes mellitus
- ◯ Forensic histopathology
- ◯ Forensic psychiatry
- ◯ Gastroenterology
- ◯ General (internal) medicine
- ◯ General practice
- ◯ General psychiatry
- ◯ General surgery
- ◯ Genitourinary medicine
- ◯ Geriatric medicine
- ◯ Gynaecological oncology
- ◯ Haematology
- ◯ Hepatology
- ◯ Histopathology
- ◯ Immunology
- ◯ Infectious diseases
- ◯ Intensive care medicine
- ◯ Interventional radiology
- ◯ Liaison psychiatry
- ◯ Maternal and fetal medicine
- ◯ Medical microbiology
- ◯ Medical oncology
- ◯ Medical ophthalmology
- ◯ Medical psychotherapy
- ◯ Medical virology
- ◯ Metabolic medicine
- ◯ Neonatal medicine
- ◯ Neurology
- ◯ Neurosurgery
- ◯ Nuclear medicine
- ◯ Obstetrics and gynaecology
- ◯ Occupational medicine
- ◯ Old age psychiatry
- ◯ Ophthalmology
- ◯ Oral and maxillofacial surgery
- ◯ Otolaryngology
- ◯ Paediatric allergy, immunology and infectious diseases
- ◯ Paediatric clinical pharmacology and therapeutics
- ◯ Paediatric diabetes and endocrinology
- ◯ Paediatric emergency medicine
- ◯ Paediatric gastroenterology, hepatology and nutrition
- ◯ Paediatric inherited metabolic medicine
- ◯ Paediatric intensive care medicine
- ◯ Paediatric nephrology
- ◯ Paediatric neurodisability

For peer review only
For peer review only - http://bmjopen.bmj.com/site/about/guidelines.xhtml

◯ Paediatric neurology
◯ Paediatric oncology
◯ Paediatric palliative medicine
◯ Paediatric respiratory medicine
◯ Paediatric rheumatology
◯ Paediatric and perinatal pathology
◯ Paediatric cardiology
◯ Paediatric surgery
◯ Paediatrics
◯ Palliative medicine
◯ Pharmaceutical medicine
◯ Plastic surgery
◯ Pre-hospital emergency medicine
◯ Psychiatry of learning disability
◯ Public health medicine
◯ Rehabilitation medicine
◯ Rehabilitation psychiatry
◯ Renal medicine
◯ Reproductive medicine
◯ Respiratory medicine
◯ Rheumatology
◯ Sport and exercise medicine
◯ Stroke medicine
◯ Substance misuse psychiatry
◯ Trauma and orthopaedic surgery
◯ Tropical medicine
◯ Urogynaecology
◯ Urology
◯ Vascular surgery
◯ Prefer not to answer

For peer review only

What was your specialty in the first month after the start of the UK national lockdown on 23rd March 2020?

- ◯ Acute internal medicine
- ◯ Allergy
- ◯ Anaesthetics
- ◯ Audio vestibular medicine
- ◯ Aviation and space medicine
- ◯ Cardio-thoracic surgery
- ◯ Cardiology
- ◯ Chemical pathology
- ◯ Child and adolescent psychiatry
- ◯ Child mental health
- ◯ Clinical genetics
- ◯ Clinical neurophysiology
- ◯ Clinical oncology
- ◯ Clinical pharmacology and therapeutics
- ◯ Clinical radiology
- ◯ Community child health
- ◯ Community sexual and reproductive health
- ◯ Congenital cardiac surgery
- ◯ Cytopathology
- ◯ Dermatology
- ◯ Diagnostic neuropathology
- ◯ Emergency medicine
- ◯ Endocrinology and diabetes mellitus
- ◯ Forensic histopathology
- ◯ Forensic psychiatry
- ◯ Gastroenterology
- ◯ General (internal) medicine
- ◯ General practice
- ◯ General psychiatry
- ◯ General surgery
- ◯ Genitourinary medicine
- ◯ Geriatric medicine
- ◯ Gynaecological oncology
- ◯ Haematology
- ◯ Hepatology
- ◯ Histopathology
- ◯ Immunology
- ◯ Infectious diseases
- ◯ Intensive care medicine
- ◯ Interventional radiology
- ◯ Liaison psychiatry
- ◯ Maternal and fetal medicine
- ◯ Medical microbiology
- ◯ Medical oncology
- ◯ Medical ophthalmology
- ◯ Medical psychotherapy
- ◯ Medical virology
- ◯ Metabolic medicine
- ◯ Neonatal medicine
- ◯ Neurology
- ◯ Neurosurgery
- ◯ Nuclear medicine
- ◯ Obstetrics and gynaecology
- ◯ Occupational medicine
- ◯ Old age psychiatry
- ◯ Ophthalmology
- ◯ Oral and maxillofacial surgery
- ◯ Otolaryngology
- ◯ Paediatric allergy, immunology and infectious diseases
- ◯ Paediatric clinical pharmacology and therapeutics
- ◯ Paediatric diabetes and endocrinology
- ◯ Paediatric emergency medicine
- ◯ Paediatric gastroenterology, hepatology and nutrition
- ◯ Paediatric inherited metabolic medicine
- ◯ Paediatric intensive care medicine
- ◯ Paediatric nephrology
- ◯ Paediatric neurodisability

For peer review only

	◯ Paediatric neurology<br>◯ Paediatric oncology<br>◯ Paediatric palliative medicine<br>◯ Paediatric respiratory medicine<br>◯ Paediatric rheumatology<br>◯ Paediatric and perinatal pathology<br>◯ Paediatric cardiology<br>◯ Paediatric surgery<br>◯ Paediatrics<br>◯ Palliative medicine<br>◯ Pharmaceutical medicine<br>◯ Plastic surgery<br>◯ Pre-hospital emergency medicine<br>◯ Psychiatry of learning disability<br>◯ Public health medicine<br>◯ Rehabilitation medicine<br>◯ Rehabilitation psychiatry<br>◯ Renal medicine<br>◯ Reproductive medicine<br>◯ Respiratory medicine<br>◯ Rheumatology<br>◯ Sport and exercise medicine<br>◯ Stroke medicine<br>◯ Substance misuse psychiatry<br>◯ Trauma and orthopaedic surgery<br>◯ Tropical medicine<br>◯ Urogynaecology<br>◯ Urology<br>◯ Vascular surgery<br>◯ Prefer not to answer
What is your current or most recent NHS band?	◯ Band 1<br>◯ Band 2<br>◯ Band 3<br>◯ Band 4<br>◯ Band 5<br>◯ Band 6<br>◯ Band 7<br>◯ Band 8a<br>◯ Band 8b<br>◯ Band 8c<br>◯ Band 8d<br>◯ Band 9<br>◯ Prefer not to answer
What was your NHS band at the start of the UK national lockdown on 23rd March 2020?	◯ Band 1<br>◯ Band 2<br>◯ Band 3<br>◯ Band 4<br>◯ Band 5<br>◯ Band 6<br>◯ Band 7<br>◯ Band 8a<br>◯ Band 8b<br>◯ Band 8c<br>◯ Band 8d<br>◯ Band 9<br>◯ Prefer not to answer

For peer review only

What is your registered field of nursing?	◯ Adult<br>◯ Children's<br>◯ Learning disability<br>◯ Mental health<br>◯ Dual registration (please specify)<br>◯ Prefer not to answer
Please specify the two fields in which you practice:	__________________________________

For peer review only

Section 2/10: Your job

7% complete

.

Please indicate which areas you work in a typical week now, and which areas you worked in a typical week during the first month after the UK national lockdown on 23rd March 2020.

If you work in the same area(s) now as you were working in the first month after the UK national lockdown, please tick the “now” and “UK national lockdown” boxes.

Please leave blank any areas you have not worked in since the UK national lockdown.

	Now	UK national lockdown	Prefer not to answer
Ambulance (inc air ambulance)	☐	☐	☐
Armed forces	☐	☐	☐
Community clinical / primary care setting	☐	☐	☐
Community non-clinical settings	☐	☐	☐
Hospitals - Emergency Department	☐	☐	☐
Hospital - Intensive Care Unit	☐	☐	☐
Hospital - other inpatient setting	☐	☐	☐
Hospital - outpatients	☐	☐	☐
Hospital - other clinical setting	☐	☐	☐
Hospital - other non-clinical setting	☐	☐	☐
Hospital - public / communal areas	☐	☐	☐
Laboratory	☐	☐	☐
Maternity	☐	☐	☐
Mobile across areas	☐	☐	☐
Nursing or care home	☐	☐	☐
Prison	☐	☐	☐
Psychiatric hospital or inpatient unit	☐	☐	☐
University	☐	☐	☐
Your home	☐	☐	☐
Other (Please specify)	☐	☐	☐

Please specify the workplace for which you selected "Other": ______________________

At present, how many hours do you work in a typical week? ______________________

For peer review only

In the first month after the start of the UK national lockdown on 23rd March 2020, how many hours did you work in a typical week? ________________________________

How often do you work night shifts now, and did you typically work in the early months after the start of the UK national lockdown on 23rd March 2020?

If these are on call shifts, please answer based on how often you are actually required to work.
If you were not working during the specified time frame please select "Not applicable".
If you are answering this questionnaire on a smartphone, you may find it easier to view by rotating the screen.

	Not applicable	Never	Less than once a month	Once a month or more, but not every week	Once a week or more, but not every shift	I always work nights	Prefer not to answer
Now	○	○	○	○	○	○	○
In the early months following the start of the UK national lockdown on 23rd March 2020	○	○	○	○	○	○	○

For peer review only

Section 2/10: Your job

8% complete

.

How many people did you talk with at work last week from each of the following groups:

If you are answering this questionnaire on a smartphone, you may find it easier to view by rotating the screen.

Remotely (e.g. over the phone or via video media)

	0	1-5	6-20	21-50	51+	Prefer not to answer
Number of patients	◯	◯	◯	◯	◯	◯
Number of colleagues	◯	◯	◯	◯	◯	◯
Number of others (not patients or colleagues)	◯	◯	◯	◯	◯	◯

Face to face with social distancing

	0	1-5	6-20	21-50	51+	Prefer not to answer
Number of patients with confirmed or suspected COVID-19	◯	◯	◯	◯	◯	◯
Number of other patients	◯	◯	◯	◯	◯	◯
Number of colleagues	◯	◯	◯	◯	◯	◯
Number of others (not patients or colleagues)	◯	◯	◯	◯	◯	◯

With physical contact

	0	1-5	6-20	21-50	51+	Prefer not to answer
Number of patients with confirmed or suspected COVID-19	◯	◯	◯	◯	◯	◯
Number of other patients	◯	◯	◯	◯	◯	◯
Number of colleagues	◯	◯	◯	◯	◯	◯
Number of others (not patients or colleagues)	◯	◯	◯	◯	◯	◯

For peer review only

Setion 2/10: Your job

8% complete
.

How many people did you talk with at work in a typical week during the first month after the start of the UK national lockdown on 23rd March 2020 from each of the following groups:
If you are answering this questionnaire on a smartphone, you may find it easier to view by rotating the screen.

Remotely (e.g. over the phone, or via video media)

	0	1-5	6-20	21-50	51+	Prefer not to answer
Number of patients	○	○	○	○	○	○
Number of colleagues	○	○	○	○	○	○
Number of others (not patients or colleagues)	○	○	○	○	○	○

Face to face with social distancing

	0	1-5	6-20	21-50	51+	Prefer not to answer
Number of patients with confirmed or suspected COVID-19	○	○	○	○	○	○
Number of other patients	○	○	○	○	○	○
Number of colleagues	○	○	○	○	○	○
Number of others (not patients or colleagues)	○	○	○	○	○	○

With physical contact

	0	1-5	6-20	21-50	51+	Prefer not to answer
Number of patients with confirmed or suspected COVID-19	○	○	○	○	○	○
Number of other patients	○	○	○	○	○	○
Number of colleagues	○	○	○	○	○	○
Number of others (not patients or colleagues)	○	○	○	○	○	○

For peer review only

Section 2/10: Your job

9% complete

.

When working in your main role/job, how long do you spend travelling to and from work per day?

You may select 'Not at all' if you work exclusively from home.

If you are answering this questionnaire on a smartphone, you may find it easier to view by rotating the screen.

	Not at all	Less than 10 minutes	10 minutes to 1 hour	1 to 2 hours	Over 2 hours	Prefer not to answer
Typical working day over the past month	○	○	○	○	○	○
Typical working day during the first month after the UK national lockdown on 23rd March 2020	○	○	○	○	○	○

Which of the following modes of transport do you use to commute on a typical working day over the past month?
Please select all that apply.

- ☐ Car, alone or with member of household
- ☐ Car share, with a small pool of people outside of household
- ☐ Taxi or private hire vehicle
- ☐ Public transport (e.g. bus, train, tram, underground)
- ☐ Motorcycle, scooter or moped
- ☐ Bicycle
- ☐ On foot
- ☐ Other

Please enter the mode of transport for which you selected 'Other': ______________________

Which of the following modes of transport did you use to commute on a typical working day, during the first month after the start of the UK national lockdown on 23 March 2020?
Please select all that apply.

- ☐ Car, alone or with member of household
- ☐ Car share, with a small pool of people outside of household
- ☐ Taxi or private hire vehicle
- ☐ Public transport (e.g. bus, train, tram, underground)
- ☐ Motorcycle, scooter or moped
- ☐ Bicycle
- ☐ On foot
- ☐ Other

Please enter the mode of transport for which you selected 'Other': ______________________

For peer review only

Section 2/10: Your job

11% complete

.

This question is about your access to appropriate personal protective equipment (PPE) at work.

If you have more than one role or job, please consider your access to personal protective equipment (PPE) in general, across all of your jobs.

	Not applicable	Not at all	Rarely	Some of the time	Yes, most of the time	Yes, all of the time	Prefer not to answer
At present, do you have access to appropriate personal protective equipment (PPE) at work?	○	○	○	○	○	○	○
In the first month after the start of the UK national lockdown on 23rd March 2020, did you have access to appropriate personal protective equipment (PPE) at work?	○	○	○	○	○	○	○

This question is about any training you may have received in the use of personal protective equipment (PPE) in your main job/role.

Select all that apply.

	Not applicable	Formal training in person	Formal training online	Informal training	No training	Prefer not to answer
Have you received training in the use of personal protective equipment (PPE) for your current work?	☐	☐	☐	☐	☐	☐
In the first month after the start of the UK national lockdown on 23rd March 2020, did you receive training in the use of personal protective equipment (PPE) for your work?	☐	☐	☐	☐	☐	☐

This question is about your exposure to aerosol-generating procedures at work.

Aerosol generating procedures include intubation, extubation, bronchoscopy, non-invasive ventilation, respiratory suctioning, and dental procedures using high speed drills.

For peer review only

	Not applicable	Never	Once a month or less	A few times a month	Once a week	A few times a week	Every day	Prefer not to answer
At present, how often are you in a room where aerosol-generating procedures are performed?	○	○	○	○	○	○	○	○
In the first month after the start of the UK national lockdown on 23rd March 2020, how often were you in a room where aerosol-generating procedures are performed?	○	○	○	○	○	○	○	○

For peer review only

Section 2/10: Your job

13% complete

.

Have you been offered an NHS COVID-19 risk assessment at work?

- ◯ Not applicable
- ◯ No, I have not heard of COVID-19 risk assessments
- ◯ No, I have not been offered a COVID-19 risk assessment
- ◯ Yes, I have been offered a COVID-19 risk assessment but I chose not to complete it
- ◯ Yes, I have been offered a COVID-19 risk assessment and I completed it
- ◯ Prefer not to answer

Did your work change as a result of the NHS COVID-19 risk assessment result? (Select the best answer)

- ◯ No, because it did not need to
- ◯ No, because I did not want it to
- ◯ No, but I did want it to
- ◯ Yes, different duties (e.g. advised not to conduct aerosol-generating procedures)
- ◯ Yes, reduced patient contact
- ◯ Yes, removed patient contact
- ◯ Yes, advised to shield
- ◯ Yes, other (please specify)
- ◯ Prefer not to answer

Please specify how your work changed as a result of the NHS COVID-19 risk assessment result: __________________________________

Thinking about where you work in your current main job/role, to what extent do you agree with the following statements?
If you are answering this questionnaire on a smartphone, you may find it easier to view by rotating the screen.

	Strongly disagree	Disagree	Neither agree or disagree	Agree	Strongly agree	Not applicable	Prefer not to answer
I would feel secure raising concerns about unsafe clinical practice	◯	◯	◯	◯	◯	◯	◯
I am confident that my organisation would address my concern	◯	◯	◯	◯	◯	◯	◯

For peer review only

Section 2/10: Your job

14% complete

.

During the UK national lockdown that began on 23rd March 2020, were you redeployed to a different role because of the pandemic?	◯ No<br>◯ Yes, I was redeployed but continued to work in my role prior to the UK national lockdown more than half the time<br>◯ Yes, I was redeployed but continued to work in my role prior to the UK national lockdown for less than half the time<br>◯ Yes, I was redeployed all of the time<br>◯ Prefer not to answer
Compared to your role before the start of UK national lockdown on 23 March 2020, how much direct patient contact is there or was there in your redeployed role?	◯ Much less<br>◯ Somewhat less<br>◯ About the same<br>◯ Somewhat more<br>◯ Much more<br>◯ Prefer not to answer
Did you have any of the following in your redeployment?<br><br>Select all that apply	☐ Formal training face to face<br>☐ Formal training online<br>☐ Informal training<br>☐ No training<br>☐ Prefer not to answer
Did you have any of the following types of supervision in your redeployment?<br>Select all that apply	☐ Formal supervision<br>☐ Informal supervision<br>☐ No supervision<br>☐ Prefer not to answer

For peer review only

Section 3/10: Your ethnic, cultural and national identity and background

17% complete

.

Ethnicity relates to a range of different factors. Most studies of ethnicity in healthcare workers use the standard UK Census question on ethnicity with its broad but limited ethnic groupings.

We will start with the UK Census ethnicity question but we recognise its limitations in describing the subtleties of how people vary in relation to migration, social identity, and experiences of them and their families. We hope this more detailed information will help in understanding how COVID-19 affects different groups.

This section asks about:

• Ethnic group and place of birth of you and your parents
• English and other languages spoken
• The role of religion in your life
• Your sense of identity
• The ethnicity of those you work with

Please remember that if you prefer not to answer any question then simply click on Prefer not to answer, but remember that there are good reasons for asking these questions

For peer review only

Section 3/10: Your ethnic, cultural and national identity and background

17% complete
.

What is your ethnic group?

Select the one that best describes your ethnic group or background.
The categories are the ethnic groups used in the UK National Census.

- ◯ Asian/Asian British - Indian
- ◯ Asian/Asian British - Pakistani
- ◯ Asian/Asian British - Bangladeshi
- ◯ Asian/Asian British - Chinese
- ◯ Asian/Asian British - Any other Asian background
- ◯ Black/African/Caribbean/Black British - African
- ◯ Black/African/Caribbean/Black British - Caribbean
- ◯ Black/African/Caribbean/Black British - Any other Black/African/Caribbean background
- ◯ Mixed/Multiple ethnic groups - White and Black Caribbean
- ◯ Mixed/Multiple ethnic groups - White and Black African
- ◯ Mixed/Multiple ethnic groups - White and Asian
- ◯ Mixed/Multiple ethnic groups - Any other Mixed/multiple ethnic background
- ◯ White - English/Welsh/Scottish/Northern Irish/British
- ◯ White - Irish
- ◯ White - Gypsy or Irish Traveller
- ◯ White - Any other white background
- ◯ Other ethnic group - Arab
- ◯ Other ethnic group - Any other ethnic background
- ◯ Prefer not to say

Please specify your ethnic group: ______________________________

Were you born in the UK?

- ◯ No
- ◯ Yes
- ◯ Prefer not to answer

For peer review only

In which country were you born?

◯ Afghanistan
◯ Albania
◯ Algeria
◯ Andorra
◯ Angola
◯ Antigua and Barbuda
◯ Argentina
◯ Armenia
◯ Australia
◯ Austria
◯ Azerbaijan
◯ Bahamas, The
◯ Bahrain
◯ Bangladesh
◯ Barbados
◯ Belarus
◯ Belgium
◯ Belize
◯ Benin
◯ Bhutan
◯ Bolivia
◯ Bosnia and Herzegovina
◯ Botswana
◯ Brazil
◯ Brunei
◯ Bulgaria
◯ Burkina Faso
◯ Burundi
◯ Cambodia
◯ Cameroon
◯ Canada
◯ Cape Verde
◯ Central African Republic
◯ Chad
◯ Chile
◯ China
◯ Colombia
◯ Comoros
◯ Congo
◯ Congo (Democratic Republic)
◯ Costa Rica
◯ Croatia
◯ Cuba
◯ Cyprus
◯ Czechia
◯ Denmark
◯ Djibouti
◯ Dominica
◯ Dominican Republic
◯ East Timor
◯ Ecuador
◯ Egypt
◯ El Salvador
◯ Equatorial Guinea
◯ Eritrea
◯ Estonia
◯ Eswatini
◯ Ethiopia
◯ Fiji
◯ Finland
◯ France
◯ Gabon
◯ Gambia, The
◯ Georgia
◯ Germany
◯ Ghana
◯ Greece
◯ Grenada
◯ Guatemala

For peer review only

◯ Guinea Republic of Guinea
◯ Guinea-Bissau
◯ Guyana
◯ Haiti
◯ Honduras
◯ Hungary
◯ Iceland
◯ India
◯ Indonesia
◯ Iran
◯ Iraq
◯ Ireland
◯ Israel
◯ Italy
◯ Ivory Coast
◯ Jamaica
◯ Japan
◯ Jordan
◯ Kazakhstan
◯ Kenya
◯ Kiribati
◯ Korea (North)
◯ Korea (South)
◯ Kosovo
◯ Kuwait
◯ Kyrgyzstan
◯ Laos
◯ Latvia
◯ Lebanon
◯ Lesotho
◯ Liberia
◯ Libya
◯ Liechtenstein
◯ Lithuania
◯ Luxembourg
◯ Madagascar
◯ Malawi
◯ Malaysia
◯ Maldives
◯ Mali
◯ Malta
◯ Marshall Islands
◯ Mauritania
◯ Mauritius
◯ Mexico
◯ Micronesia
◯ Moldova
◯ Monaco
◯ Mongolia
◯ Montenegro
◯ Morocco
◯ Mozambique
◯ Myanmar (Burma)
◯ Namibia
◯ Nauru
◯ Nepal
◯ Netherlands
◯ New Zealand
◯ Nicaragua
◯ Niger
◯ Nigeria
◯ North Macedonia
◯ Norway
◯ Oman
◯ Pakistan
◯ Palau
◯ Panama
◯ Papua New Guinea
◯ Paraguay
◯ Peru
◯ Philippines

For peer review only

◯ Poland
◯ Portugal
◯ Qatar
◯ Romania
◯ Russia
◯ Rwanda
◯ St Kitts and Nevis
◯ St Lucia
◯ St Vincent
◯ Samoa
◯ San Marino
◯ Sao Tome and Principe
◯ Saudi Arabia
◯ Senegal
◯ Serbia
◯ Seychelles
◯ Sierra Leone
◯ Singapore
◯ Slovakia
◯ Slovenia
◯ Solomon Islands
◯ Somalia
◯ South Africa
◯ South Sudan
◯ Spain
◯ Sri Lanka
◯ Sudan
◯ Suriname
◯ Sweden
◯ Switzerland
◯ Syria
◯ Taiwan
◯ Tajikistan
◯ Tanzania
◯ Thailand
◯ Togo
◯ Tonga
◯ Trinidad and Tobago
◯ Tunisia
◯ Turkey
◯ Turkmenistan
◯ Tuvalu
◯ Uganda
◯ Ukraine
◯ United Arab Emirates
◯ United States
◯ Uruguay
◯ Uzbekistan
◯ Vanuatu
◯ Vatican City
◯ Venezuela
◯ Vietnam
◯ Yemen
◯ Zambia
◯ Zimbabwe
◯ Other (Please specify)
◯ Prefer not to answer

Please specify the country in which you were born: ____________________

In which year did you move to the UK?

If you are unsure, please give your best estimate. ____________________ ((Please enter in format YYYY, e.g. 1967))

For peer review only

For which countries do you hold a passport?

We have provided boxes to select up to three nationalities. Please leave the boxes for nationality 2 and nationality 3 blank if they do not apply to you.

For peer review only

Nationality 1:

◯ United Kingdom ◯ Afghanistan
◯ Albania ◯ Algeria ◯ Andorra
◯ Angola ◯ Antigua and Barbuda
◯ Argentina ◯ Armenia
◯ Australia ◯ Austria
◯ Azerbaijan ◯ Bahamas, The
◯ Bahrain ◯ Bangladesh
◯ Barbados ◯ Belarus
◯ Belgium ◯ Belize ◯ Benin
◯ Bhutan ◯ Bolivia ◯ Bosnia and Herzegovina ◯ Botswana
◯ Brazil ◯ Brunei ◯ Bulgaria
◯ Burkina Faso ◯ Burundi
◯ Cambodia ◯ Cameroon
◯ Canada ◯ Cape Verde
◯ Central African Republic
◯ Chad ◯ Chile ◯ China
◯ Colombia ◯ Comoros
◯ Congo ◯ Congo (Democratic Republic)
◯ Costa Rica ◯ Croatia
◯ Cuba ◯ Cyprus ◯ Czechia
◯ Denmark ◯ Djibouti
◯ Dominica ◯ Dominican Republic
◯ East Timor ◯ Ecuador
◯ Egypt ◯ El Salvador
◯ Equatorial Guinea ◯ Eritrea
◯ Estonia ◯ Eswatini
◯ Ethiopia ◯ Fiji ◯ Finland
◯ France ◯ Gabon ◯ Gambia, The
◯ Georgia ◯ Germany
◯ Ghana ◯ Greece ◯ Grenada
◯ Guatemala ◯ Guinea Republic of Guinea
◯ Guinea-Bissau ◯ Guyana
◯ Haiti ◯ Honduras ◯ Hungary
◯ Iceland ◯ India ◯ Indonesia
◯ Iran ◯ Iraq ◯ Ireland
◯ Israel ◯ Italy ◯ Ivory Coast
◯ Jamaica ◯ Japan ◯ Jordan
◯ Kazakhstan ◯ Kenya
◯ Kiribati ◯ Korea (North)
◯ Korea (South) ◯ Kosovo
◯ Kuwait ◯ Kyrgyzstan
◯ Laos ◯ Latvia ◯ Lebanon
◯ Lesotho ◯ Liberia
◯ Libya ◯ Liechtenstein
◯ Lithuania ◯ Luxembourg
◯ Madagascar ◯ Malawi
◯ Malaysia ◯ Maldives
◯ Mali ◯ Malta ◯ Marshall Islands
◯ Mauritania ◯ Mauritius
◯ Mexico ◯ Micronesia
◯ Moldova ◯ Monaco
◯ Mongolia ◯ Montenegro
◯ Morocco ◯ Mozambique
◯ Myanmar (Burma) ◯ Namibia
◯ Nauru ◯ Nepal ◯ Netherlands
◯ New Zealand ◯ Nicaragua
◯ Niger ◯ Nigeria ◯ North Macedonia
◯ Norway ◯ Oman ◯ Pakistan
◯ Palau ◯ Panama ◯ Papua New Guinea
◯ Paraguay ◯ Peru ◯ Philippines
◯ Poland ◯ Portugal
◯ Qatar ◯ Romania ◯ Russia
◯ Rwanda ◯ St Kitts and Nevis
◯ St Lucia ◯ St Vincent
◯ Samoa ◯ San Marino
◯ Sao Tome and Principe
◯ Saudi Arabia ◯ Senegal
◯ Serbia ◯ Seychelles

For peer review only

For peer review only - http://bmjopen.bmj.com/site/about/guidelines.xhtml

◯ Sierra Leone ◯ Singapore
◯ Slovakia ◯ Slovenia
◯ Solomon Islands ◯ Somalia
◯ South Africa ◯ South Sudan
◯ Spain ◯ Sri Lanka
◯ Sudan ◯ Suriname
◯ Sweden ◯ Switzerland
◯ Syria ◯ Taiwan ◯ Tajikistan
◯ Tanzania ◯ Thailand
◯ Togo ◯ Tonga ◯ Trinidad and Tobago
◯ Tunisia ◯ Turkey ◯ Turkmenistan
◯ Tuvalu ◯ Uganda ◯ Ukraine
◯ United Arab Emirates
◯ United States ◯ Uruguay
◯ Uzbekistan ◯ Vanuatu
◯ Vatican City ◯ Venezuela
◯ Vietnam ◯ Yemen
◯ Zambia ◯ Zimbabwe
◯ Other (Please specify)
◯ Prefer not to answer

Please specify nationality 1: ________________________________

For peer review only

Nationality 2:

◯ United Kingdom ◯ Afghanistan
◯ Albania ◯ Algeria ◯ Andorra
◯ Angola ◯ Antigua and Barbuda
◯ Argentina ◯ Armenia
◯ Australia ◯ Austria
◯ Azerbaijan ◯ Bahamas, The
◯ Bahrain ◯ Bangladesh
◯ Barbados ◯ Belarus
◯ Belgium ◯ Belize ◯ Benin
◯ Bhutan ◯ Bolivia ◯ Bosnia and Herzegovina ◯ Botswana
◯ Brazil ◯ Brunei ◯ Bulgaria
◯ Burkina Faso ◯ Burundi
◯ Cambodia ◯ Cameroon
◯ Canada ◯ Cape Verde
◯ Central African Republic
◯ Chad ◯ Chile ◯ China
◯ Colombia ◯ Comoros
◯ Congo ◯ Congo (Democratic Republic)
◯ Costa Rica ◯ Croatia
◯ Cuba ◯ Cyprus ◯ Czechia
◯ Denmark ◯ Djibouti
◯ Dominica ◯ Dominican Republic
◯ East Timor ◯ Ecuador
◯ Egypt ◯ El Salvador
◯ Equatorial Guinea ◯ Eritrea
◯ Estonia ◯ Eswatini
◯ Ethiopia ◯ Fiji ◯ Finland
◯ France ◯ Gabon ◯ Gambia, The
◯ Georgia ◯ Germany
◯ Ghana ◯ Greece ◯ Grenada
◯ Guatemala ◯ Guinea Republic of Guinea
◯ Guinea-Bissau ◯ Guyana
◯ Haiti ◯ Honduras ◯ Hungary
◯ Iceland ◯ India ◯ Indonesia
◯ Iran ◯ Iraq ◯ Ireland
◯ Israel ◯ Italy ◯ Ivory Coast
◯ Jamaica ◯ Japan ◯ Jordan
◯ Kazakhstan ◯ Kenya
◯ Kiribati ◯ Korea (North)
◯ Korea (South) ◯ Kosovo
◯ Kuwait ◯ Kyrgyzstan
◯ Laos ◯ Latvia ◯ Lebanon
◯ Lesotho ◯ Liberia
◯ Libya ◯ Liechtenstein
◯ Lithuania ◯ Luxembourg
◯ Madagascar ◯ Malawi
◯ Malaysia ◯ Maldives
◯ Mali ◯ Malta ◯ Marshall Islands
◯ Mauritania ◯ Mauritius
◯ Mexico ◯ Micronesia
◯ Moldova ◯ Monaco
◯ Mongolia ◯ Montenegro
◯ Morocco ◯ Mozambique
◯ Myanmar (Burma) ◯ Namibia
◯ Nauru ◯ Nepal ◯ Netherlands
◯ New Zealand ◯ Nicaragua
◯ Niger ◯ Nigeria ◯ North Macedonia
◯ Norway ◯ Oman ◯ Pakistan
◯ Palau ◯ Panama ◯ Papua New Guinea
◯ Paraguay ◯ Peru ◯ Philippines
◯ Poland ◯ Portugal
◯ Qatar ◯ Romania ◯ Russia
◯ Rwanda ◯ St Kitts and Nevis
◯ St Lucia ◯ St Vincent
◯ Samoa ◯ San Marino
◯ Sao Tome and Principe
◯ Saudi Arabia ◯ Senegal
◯ Serbia ◯ Seychelles

For peer review only
For peer review only - http://bmjopen.bmj.com/site/about/guidelines.xhtml

◯ Sierra Leone ◯ Singapore
◯ Slovakia ◯ Slovenia
◯ Solomon Islands ◯ Somalia
◯ South Africa ◯ South Sudan
◯ Spain ◯ Sri Lanka
◯ Sudan ◯ Suriname
◯ Sweden ◯ Switzerland
◯ Syria ◯ Taiwan ◯ Tajikistan
◯ Tanzania ◯ Thailand
◯ Togo ◯ Tonga ◯ Trinidad and Tobago
◯ Tunisia ◯ Turkey ◯ Turkmenistan
◯ Tuvalu ◯ Uganda ◯ Ukraine
◯ United Arab Emirates
◯ United States ◯ Uruguay
◯ Uzbekistan ◯ Vanuatu
◯ Vatican City ◯ Venezuela
◯ Vietnam ◯ Yemen
◯ Zambia ◯ Zimbabwe
◯ Other (Please specify)
◯ Prefer not to answer

Please specify nationality 2: ________________________________

For peer review only

Nationality 3:

◯ United Kingdom ◯ Afghanistan
◯ Albania ◯ Algeria ◯ Andorra
◯ Angola ◯ Antigua and Barbuda
◯ Argentina ◯ Armenia
◯ Australia ◯ Austria
◯ Azerbaijan ◯ Bahamas, The
◯ Bahrain ◯ Bangladesh
◯ Barbados ◯ Belarus
◯ Belgium ◯ Belize ◯ Benin
◯ Bhutan ◯ Bolivia ◯ Bosnia and Herzegovina ◯ Botswana
◯ Brazil ◯ Brunei ◯ Bulgaria
◯ Burkina Faso ◯ Burundi
◯ Cambodia ◯ Cameroon
◯ Canada ◯ Cape Verde
◯ Central African Republic
◯ Chad ◯ Chile ◯ China
◯ Colombia ◯ Comoros
◯ Congo ◯ Congo (Democratic Republic)
◯ Costa Rica ◯ Croatia
◯ Cuba ◯ Cyprus ◯ Czechia
◯ Denmark ◯ Djibouti
◯ Dominica ◯ Dominican Republic
◯ East Timor ◯ Ecuador
◯ Egypt ◯ El Salvador
◯ Equatorial Guinea ◯ Eritrea
◯ Estonia ◯ Eswatini
◯ Ethiopia ◯ Fiji ◯ Finland
◯ France ◯ Gabon ◯ Gambia, The
◯ Georgia ◯ Germany
◯ Ghana ◯ Greece ◯ Grenada
◯ Guatemala ◯ Guinea Republic of Guinea
◯ Guinea-Bissau ◯ Guyana
◯ Haiti ◯ Honduras ◯ Hungary
◯ Iceland ◯ India ◯ Indonesia
◯ Iran ◯ Iraq ◯ Ireland
◯ Israel ◯ Italy ◯ Ivory Coast
◯ Jamaica ◯ Japan ◯ Jordan
◯ Kazakhstan ◯ Kenya
◯ Kiribati ◯ Korea (North)
◯ Korea (South) ◯ Kosovo
◯ Kuwait ◯ Kyrgyzstan
◯ Laos ◯ Latvia ◯ Lebanon
◯ Lesotho ◯ Liberia
◯ Libya ◯ Liechtenstein
◯ Lithuania ◯ Luxembourg
◯ Madagascar ◯ Malawi
◯ Malaysia ◯ Maldives
◯ Mali ◯ Malta ◯ Marshall Islands
◯ Mauritania ◯ Mauritius
◯ Mexico ◯ Micronesia
◯ Moldova ◯ Monaco
◯ Mongolia ◯ Montenegro
◯ Morocco ◯ Mozambique
◯ Myanmar (Burma) ◯ Namibia
◯ Nauru ◯ Nepal ◯ Netherlands
◯ New Zealand ◯ Nicaragua
◯ Niger ◯ Nigeria ◯ North Macedonia
◯ Norway ◯ Oman ◯ Pakistan
◯ Palau ◯ Panama ◯ Papua New Guinea
◯ Paraguay ◯ Peru ◯ Philippines
◯ Poland ◯ Portugal
◯ Qatar ◯ Romania ◯ Russia
◯ Rwanda ◯ St Kitts and Nevis
◯ St Lucia ◯ St Vincent
◯ Samoa ◯ San Marino
◯ Sao Tome and Principe
◯ Saudi Arabia ◯ Senegal
◯ Serbia ◯ Seychelles

For peer review only

For peer review only - http://bmjopen.bmj.com/site/about/guidelines.xhtml

◯ Sierra Leone ◯ Singapore
◯ Slovakia ◯ Slovenia
◯ Solomon Islands ◯ Somalia
◯ South Africa ◯ South Sudan
◯ Spain ◯ Sri Lanka
◯ Sudan ◯ Suriname
◯ Sweden ◯ Switzerland
◯ Syria ◯ Taiwan ◯ Tajikistan
◯ Tanzania ◯ Thailand
◯ Togo ◯ Tonga ◯ Trinidad and Tobago
◯ Tunisia ◯ Turkey ◯ Turkmenistan
◯ Tuvalu ◯ Uganda ◯ Ukraine
◯ United Arab Emirates
◯ United States ◯ Uruguay
◯ Uzbekistan ◯ Vanuatu
◯ Vatican City ◯ Venezuela
◯ Vietnam ◯ Yemen
◯ Zambia ◯ Zimbabwe
◯ Other (Please specify)
◯ Prefer not to answer

Please specify nationality 3: ________________________________

What is or was your partner's ethnic group?

The following categories are the ethnic groups used in the UK National Census.

◯ Asian/Asian British - Indian
◯ Asian/Asian British - Pakistani
◯ Asian/Asian British - Bangladeshi
◯ Asian/Asian British - Chinese
◯ Asian/Asian British - Any other Asian background
◯ Black/African/Caribbean/Black British - African
◯ Black/African/Caribbean/Black British - Caribbean
◯ Black/African/Caribbean/Black British - Any other Black/African/Caribbean background
◯ Mixed/Multiple ethnic groups - White and Black Caribbean
◯ Mixed/Multiple ethnic groups - White and Black African
◯ Mixed/Multiple ethnic groups - White and Asian
◯ Mixed/Multiple ethnic groups - Any other Mixed/multiple ethnic background
◯ White - English/Welsh/Scottish/Northern Irish/British
◯ White - Irish
◯ White - Gypsy or Irish Traveller
◯ White - Any other white background
◯ Other ethnic group - Arab
◯ Other ethnic group - Any other ethnic background (please specify)
◯ Prefer not to say

Please specify your partner's ethnic group: ________________________________

For peer review only

What is or was your mother's ethnic group?<br><br>The following categories are the ethnic groups used in the UK National Census.	◯ Asian/Asian British - Indian<br>◯ Asian/Asian British - Pakistani<br>◯ Asian/Asian British - Bangladeshi<br>◯ Asian/Asian British - Chinese<br>◯ Asian/Asian British - Any other Asian background<br>◯ Black/African/Caribbean/Black British - African<br>◯ Black/African/Caribbean/Black British - Caribbean<br>◯ Black/African/Caribbean/Black British - Any other Black/African/Caribbean background<br>◯ Mixed/Multiple ethnic groups - White and Black Caribbean<br>◯ Mixed/Multiple ethnic groups - White and Black African<br>◯ Mixed/Multiple ethnic groups - White and Asian<br>◯ Mixed/Multiple ethnic groups - Any other Mixed/multiple ethnic background<br>◯ White - English/Welsh/Scottish/Northern Irish/British<br>◯ White - Irish<br>◯ White - Gypsy or Irish Traveller<br>◯ White - Any other white background<br>◯ Other ethnic group - Arab<br>◯ Other ethnic group - Any other ethnic background (please specify)<br>◯ Prefer not to say
Please specify your mother's ethnic group:	__________________________________
Was your mother born in the UK?	◯ No<br>◯ Yes<br>◯ Do not know<br>◯ Prefer not to answer

For peer review only

In which country was your mother born?

- ◯ Afghanistan
- ◯ Albania
- ◯ Algeria
- ◯ Andorra
- ◯ Angola
- ◯ Antigua and Barbuda
- ◯ Argentina
- ◯ Armenia
- ◯ Australia
- ◯ Austria
- ◯ Azerbaijan
- ◯ Bahamas, The
- ◯ Bahrain
- ◯ Bangladesh
- ◯ Barbados
- ◯ Belarus
- ◯ Belgium
- ◯ Belize
- ◯ Benin
- ◯ Bhutan
- ◯ Bolivia
- ◯ Bosnia and Herzegovina
- ◯ Botswana
- ◯ Brazil
- ◯ Brunei
- ◯ Bulgaria
- ◯ Burkina Faso
- ◯ Burundi
- ◯ Cambodia
- ◯ Cameroon
- ◯ Canada
- ◯ Cape Verde
- ◯ Central African Republic
- ◯ Chad
- ◯ Chile
- ◯ China
- ◯ Colombia
- ◯ Comoros
- ◯ Congo
- ◯ Congo (Democratic Republic)
- ◯ Costa Rica
- ◯ Croatia
- ◯ Cuba
- ◯ Cyprus
- ◯ Czechia
- ◯ Denmark
- ◯ Djibouti
- ◯ Dominica
- ◯ Dominican Republic
- ◯ East Timor
- ◯ Ecuador
- ◯ Egypt
- ◯ El Salvador
- ◯ Equatorial Guinea
- ◯ Eritrea
- ◯ Estonia
- ◯ Eswatini
- ◯ Ethiopia
- ◯ Fiji
- ◯ Finland
- ◯ France
- ◯ Gabon
- ◯ Gambia, The
- ◯ Georgia
- ◯ Germany
- ◯ Ghana
- ◯ Greece
- ◯ Grenada
- ◯ Guatemala

For peer review only

For peer review only - http://bmjopen.bmj.com/site/about/guidelines.xhtml

- ◯ Guinea Republic of Guinea
- ◯ Guinea-Bissau
- ◯ Guyana
- ◯ Haiti
- ◯ Honduras
- ◯ Hungary
- ◯ Iceland
- ◯ India
- ◯ Indonesia
- ◯ Iran
- ◯ Iraq
- ◯ Ireland
- ◯ Israel
- ◯ Italy
- ◯ Ivory Coast
- ◯ Jamaica
- ◯ Japan
- ◯ Jordan
- ◯ Kazakhstan
- ◯ Kenya
- ◯ Kiribati
- ◯ Korea (North)
- ◯ Korea (South)
- ◯ Kosovo
- ◯ Kuwait
- ◯ Kyrgyzstan
- ◯ Laos
- ◯ Latvia
- ◯ Lebanon
- ◯ Lesotho
- ◯ Liberia
- ◯ Libya
- ◯ Liechtenstein
- ◯ Lithuania
- ◯ Luxembourg
- ◯ Madagascar
- ◯ Malawi
- ◯ Malaysia
- ◯ Maldives
- ◯ Mali
- ◯ Malta
- ◯ Marshall Islands
- ◯ Mauritania
- ◯ Mauritius
- ◯ Mexico
- ◯ Micronesia
- ◯ Moldova
- ◯ Monaco
- ◯ Mongolia
- ◯ Montenegro
- ◯ Morocco
- ◯ Mozambique
- ◯ Myanmar (Burma)
- ◯ Namibia
- ◯ Nauru
- ◯ Nepal
- ◯ Netherlands
- ◯ New Zealand
- ◯ Nicaragua
- ◯ Niger
- ◯ Nigeria
- ◯ North Macedonia
- ◯ Norway
- ◯ Oman
- ◯ Pakistan
- ◯ Palau
- ◯ Panama
- ◯ Papua New Guinea
- ◯ Paraguay
- ◯ Peru
- ◯ Philippines

For peer review only

◯ Poland
◯ Portugal
◯ Qatar
◯ Romania
◯ Russia
◯ Rwanda
◯ St Kitts and Nevis
◯ St Lucia
◯ St Vincent
◯ Samoa
◯ San Marino
◯ Sao Tome and Principe
◯ Saudi Arabia
◯ Senegal
◯ Serbia
◯ Seychelles
◯ Sierra Leone
◯ Singapore
◯ Slovakia
◯ Slovenia
◯ Solomon Islands
◯ Somalia
◯ South Africa
◯ South Sudan
◯ Spain
◯ Sri Lanka
◯ Sudan
◯ Suriname
◯ Sweden
◯ Switzerland
◯ Syria
◯ Taiwan
◯ Tajikistan
◯ Tanzania
◯ Thailand
◯ Togo
◯ Tonga
◯ Trinidad and Tobago
◯ Tunisia
◯ Turkey
◯ Turkmenistan
◯ Tuvalu
◯ Uganda
◯ Ukraine
◯ United Arab Emirates
◯ United States
◯ Uruguay
◯ Uzbekistan
◯ Vanuatu
◯ Vatican City
◯ Venezuela
◯ Vietnam
◯ Yemen
◯ Zambia
◯ Zimbabwe
◯ Other (Please specify)
◯ Prefer not to answer

Please specify the country in which your mother was born: ________________________________

For peer review only

What is or was your father's ethnic group?

The following categories are the ethnic groups used in the UK National Census.

- ◯ Asian/Asian British - Indian
- ◯ Asian/Asian British - Pakistani
- ◯ Asian/Asian British - Bangladeshi
- ◯ Asian/Asian British - Chinese
- ◯ Asian/Asian British - Any other Asian background
- ◯ Black/African/Caribbean/Black British - African
- ◯ Black/African/Caribbean/Black British - Caribbean
- ◯ Black/African/Caribbean/Black British - Any other Black/African/Caribbean background
- ◯ Mixed/Multiple ethnic groups - White and Black Caribbean
- ◯ Mixed/Multiple ethnic groups - White and Black African
- ◯ Mixed/Multiple ethnic groups - White and Asian
- ◯ Mixed/Multiple ethnic groups - Any other Mixed/multiple ethnic background
- ◯ White - English/Welsh/Scottish/Northern Irish/British
- ◯ White - Irish
- ◯ White - Gypsy or Irish Traveller
- ◯ White - Any other white background
- ◯ Other ethnic group - Arab
- ◯ Other ethnic group - Any other ethnic background (please specify)
- ◯ Prefer not to say

Please specify your father's ethnic group: __________________________________

Was your father born in the UK?

- ◯ No
- ◯ Yes
- ◯ Do not know
- ◯ Prefer not to answer

For peer review only

In which country was your father born?

- ◯ Afghanistan
- ◯ Albania
- ◯ Algeria
- ◯ Andorra
- ◯ Angola
- ◯ Antigua and Barbuda
- ◯ Argentina
- ◯ Armenia
- ◯ Australia
- ◯ Austria
- ◯ Azerbaijan
- ◯ Bahamas, The
- ◯ Bahrain
- ◯ Bangladesh
- ◯ Barbados
- ◯ Belarus
- ◯ Belgium
- ◯ Belize
- ◯ Benin
- ◯ Bhutan
- ◯ Bolivia
- ◯ Bosnia and Herzegovina
- ◯ Botswana
- ◯ Brazil
- ◯ Brunei
- ◯ Bulgaria
- ◯ Burkina Faso
- ◯ Burundi
- ◯ Cambodia
- ◯ Cameroon
- ◯ Canada
- ◯ Cape Verde
- ◯ Central African Republic
- ◯ Chad
- ◯ Chile
- ◯ China
- ◯ Colombia
- ◯ Comoros
- ◯ Congo
- ◯ Congo (Democratic Republic)
- ◯ Costa Rica
- ◯ Croatia
- ◯ Cuba
- ◯ Cyprus
- ◯ Czechia
- ◯ Denmark
- ◯ Djibouti
- ◯ Dominica
- ◯ Dominican Republic
- ◯ East Timor
- ◯ Ecuador
- ◯ Egypt
- ◯ El Salvador
- ◯ Equatorial Guinea
- ◯ Eritrea
- ◯ Estonia
- ◯ Eswatini
- ◯ Ethiopia
- ◯ Fiji
- ◯ Finland
- ◯ France
- ◯ Gabon
- ◯ Gambia, The
- ◯ Georgia
- ◯ Germany
- ◯ Ghana
- ◯ Greece
- ◯ Grenada
- ◯ Guatemala

For peer review only

◯ Guinea Republic of Guinea
◯ Guinea-Bissau
◯ Guyana
◯ Haiti
◯ Honduras
◯ Hungary
◯ Iceland
◯ India
◯ Indonesia
◯ Iran
◯ Iraq
◯ Ireland
◯ Israel
◯ Italy
◯ Ivory Coast
◯ Jamaica
◯ Japan
◯ Jordan
◯ Kazakhstan
◯ Kenya
◯ Kiribati
◯ Korea (North)
◯ Korea (South)
◯ Kosovo
◯ Kuwait
◯ Kyrgyzstan
◯ Laos
◯ Latvia
◯ Lebanon
◯ Lesotho
◯ Liberia
◯ Libya
◯ Liechtenstein
◯ Lithuania
◯ Luxembourg
◯ Madagascar
◯ Malawi
◯ Malaysia
◯ Maldives
◯ Mali
◯ Malta
◯ Marshall Islands
◯ Mauritania
◯ Mauritius
◯ Mexico
◯ Micronesia
◯ Moldova
◯ Monaco
◯ Mongolia
◯ Montenegro
◯ Morocco
◯ Mozambique
◯ Myanmar (Burma)
◯ Namibia
◯ Nauru
◯ Nepal
◯ Netherlands
◯ New Zealand
◯ Nicaragua
◯ Niger
◯ Nigeria
◯ North Macedonia
◯ Norway
◯ Oman
◯ Pakistan
◯ Palau
◯ Panama
◯ Papua New Guinea
◯ Paraguay
◯ Peru
◯ Philippines

For peer review only

- ◯ Poland
- ◯ Portugal
- ◯ Qatar
- ◯ Romania
- ◯ Russia
- ◯ Rwanda
- ◯ St Kitts and Nevis
- ◯ St Lucia
- ◯ St Vincent
- ◯ Samoa
- ◯ San Marino
- ◯ Sao Tome and Principe
- ◯ Saudi Arabia
- ◯ Senegal
- ◯ Serbia
- ◯ Seychelles
- ◯ Sierra Leone
- ◯ Singapore
- ◯ Slovakia
- ◯ Slovenia
- ◯ Solomon Islands
- ◯ Somalia
- ◯ South Africa
- ◯ South Sudan
- ◯ Spain
- ◯ Sri Lanka
- ◯ Sudan
- ◯ Suriname
- ◯ Sweden
- ◯ Switzerland
- ◯ Syria
- ◯ Taiwan
- ◯ Tajikistan
- ◯ Tanzania
- ◯ Thailand
- ◯ Togo
- ◯ Tonga
- ◯ Trinidad and Tobago
- ◯ Tunisia
- ◯ Turkey
- ◯ Turkmenistan
- ◯ Tuvalu
- ◯ Uganda
- ◯ Ukraine
- ◯ United Arab Emirates
- ◯ United States
- ◯ Uruguay
- ◯ Uzbekistan
- ◯ Vanuatu
- ◯ Vatican City
- ◯ Venezuela
- ◯ Vietnam
- ◯ Yemen
- ◯ Zambia
- ◯ Zimbabwe
- ◯ Other (Please specify)
- ◯ Prefer not to answer

Please specify the country in which your father was born: ________________________________

Were your grandparents born in the UK?

For peer review only

	NOT born in the UK	Born in the UK	Do not know	Prefer not to answer
Your mother's mother	◯	◯	◯	◯
Your mother's father	◯	◯	◯	◯
Your father's mother	◯	◯	◯	◯
Your father's father	◯	◯	◯	◯

For peer review only

Section 3/10: Your ethnic, cultural and national identity and background

24% complete

.

How old were you when you first learned English?

- ◯ Always spoken it
- ◯ 2-5 years
- ◯ 6-10 years
- ◯ 11-17 years
- ◯ 18+
- ◯ Prefer not to answer

What language or languages do you speak most often at home?

If you only speak one language at home, please leave the box for "Language 2" blank.

For peer review only

Language 1	◯ English<br>◯ Afrikaans<br>◯ Akan<br>◯ Arabic<br>◯ Bengali<br>◯ Bulgarian<br>◯ Burmese<br>◯ Cebuano<br>◯ Chinese<br>◯ Czech<br>◯ Dutch<br>◯ French<br>◯ German<br>◯ Greek<br>◯ Gujarati<br>◯ Hausa<br>◯ Hindi<br>◯ Hungarian<br>◯ Igbo<br>◯ Irish<br>◯ Italian<br>◯ Kurdish<br>◯ Latvian<br>◯ Lithuanian<br>◯ Malay<br>◯ Malayalam<br>◯ Maltese<br>◯ Maori<br>◯ Ndebele<br>◯ Nepali<br>◯ Persian/Farsi<br>◯ Polish<br>◯ Portuguese<br>◯ Punjabi<br>◯ Romanian<br>◯ Russian<br>◯ Shona<br>◯ Sinhala<br>◯ Slovakian<br>◯ Somali<br>◯ Spanish<br>◯ Swahili<br>◯ Swedish<br>◯ Tagalog<br>◯ Tamil<br>◯ Turkish<br>◯ Twi<br>◯ Ukrainian<br>◯ Urdu<br>◯ Welsh<br>◯ Xhosa<br>◯ Yoruba<br>◯ Zulu<br>◯ Other (Please specify)<br>◯ Prefer not to answer
Please specify language 1:	__________________________________

For peer review only

Language 2	◯ English<br>◯ Afrikaans<br>◯ Akan<br>◯ Arabic<br>◯ Bengali<br>◯ Bulgarian<br>◯ Burmese<br>◯ Cebuano<br>◯ Chinese<br>◯ Czech<br>◯ Dutch<br>◯ French<br>◯ German<br>◯ Greek<br>◯ Gujarati<br>◯ Hausa<br>◯ Hindi<br>◯ Hungarian<br>◯ Igbo<br>◯ Irish<br>◯ Italian<br>◯ Kurdish<br>◯ Latvian<br>◯ Lithuanian<br>◯ Malay<br>◯ Malayalam<br>◯ Maltese<br>◯ Maori<br>◯ Ndebele<br>◯ Nepali<br>◯ Persian/Farsi<br>◯ Polish<br>◯ Portuguese<br>◯ Punjabi<br>◯ Romanian<br>◯ Russian<br>◯ Shona<br>◯ Sinhala<br>◯ Slovakian<br>◯ Somali<br>◯ Spanish<br>◯ Swahili<br>◯ Swedish<br>◯ Tagalog<br>◯ Tamil<br>◯ Turkish<br>◯ Twi<br>◯ Ukrainian<br>◯ Urdu<br>◯ Welsh<br>◯ Xhosa<br>◯ Yoruba<br>◯ Zulu<br>◯ Other (Please specify)<br>◯ Prefer not to answer
Please specify language 2:	__________________________

For peer review only

What language was spoken in your home when you were a child?<br>If more than one language was spoken, please give them all.	☐ English<br>☐ Afrikaans<br>☐ Akan<br>☐ Arabic<br>☐ Bengali<br>☐ Bulgarian<br>☐ Burmese<br>☐ Cebuano<br>☐ Chinese<br>☐ Czech<br>☐ Dutch<br>☐ French<br>☐ German<br>☐ Greek<br>☐ Gujarati<br>☐ Hausa<br>☐ Hindi<br>☐ Hungarian<br>☐ Igbo<br>☐ Irish<br>☐ Italian<br>☐ Kurdish<br>☐ Latvian<br>☐ Lithuanian<br>☐ Malay<br>☐ Malayalam<br>☐ Maltese<br>☐ Maori<br>☐ Ndebele<br>☐ Nepali<br>☐ Persian/Farsi<br>☐ Polish<br>☐ Portuguese<br>☐ Punjabi<br>☐ Romanian<br>☐ Russian<br>☐ Shona<br>☐ Sinhala<br>☐ Slovakian<br>☐ Somali<br>☐ Spanish<br>☐ Swahili<br>☐ Swedish<br>☐ Tagalog<br>☐ Tamil<br>☐ Turkish<br>☐ Twi<br>☐ Ukrainian<br>☐ Urdu<br>☐ Welsh<br>☐ Xhosa<br>☐ Yoruba<br>☐ Zulu<br>☐ Other (Please specify)<br>☐ Prefer not to answer
Please specify the language spoken in your home when you were a child for which you selected "Other":	__________________________

For peer review only

Section 3/10: Your ethnic, cultural and national identity and background

26% complete

.

What is your religion?

This is the question that was asked in the 2011 UK Census.

- ◯ No religion
- ◯ Christian (including Church of England, Catholic, Protestant and all other Christian denominations)
- ◯ Buddhist
- ◯ Hindu
- ◯ Jewish
- ◯ Muslim
- ◯ Sikh
- ◯ Any other religion (please specify)
- ◯ Prefer not to answer

Please specify your religion: __________________________________

How important is religion to you in your everyday life?

- ◯ Not at all important
- ◯ Fairly important
- ◯ Very important
- ◯ Extremely important
- ◯ Prefer not to answer

How important was religion in your upbringing?

- ◯ Not at all important
- ◯ Fairly important
- ◯ Very important
- ◯ Extremely important
- ◯ Prefer not to answer

How often would you usually attend a holy place or a place of worship outside your home?

- ◯ Never
- ◯ On festive occasions only
- ◯ Once every few months
- ◯ About once a month
- ◯ Most weeks
- ◯ More than once a week
- ◯ Prefer not to answer

For peer review only

Section 3/10: Your ethnic, cultural and national identity and background

28% complete

.

How important is your ethnic and cultural background to your identity?

Use the scale of 0 to 10, where 0 means 'not at all important', and 10 means 'extremely important'.

- ◯ 0 Not at all important
- ◯ 1
- ◯ 2
- ◯ 3
- ◯ 4
- ◯ 5
- ◯ 6
- ◯ 7
- ◯ 8
- ◯ 9
- ◯ 10 Extremely important
- ◯ Prefer not to answer

How important is/was your mother's ethnic and cultural background to your identity?

Use the scale of 0 to 10, where 0 means 'not at all important', and 10 means 'extremely important'.

- ◯ 0 Not at all important
- ◯ 1
- ◯ 2
- ◯ 3
- ◯ 4
- ◯ 5
- ◯ 6
- ◯ 7
- ◯ 8
- ◯ 9
- ◯ 10 Extremely important
- ◯ Prefer not to answer

How important is/was your father's ethnic and cultural background to your identity?

Use the scale of 0 to 10, where 0 means 'not at all important', and 10 means 'extremely important'.

- ◯ 0 Not at all important
- ◯ 1
- ◯ 2
- ◯ 3
- ◯ 4
- ◯ 5
- ◯ 6
- ◯ 7
- ◯ 8
- ◯ 9
- ◯ 10 Extremely important
- ◯ Prefer not to answer

How important is/was your partner's ethnic and cultural background to your identity?

Use the scale of 0 to 10, where 0 means 'not at all important', and 10 means 'extremely important'.

- ◯ 0 Not at all important
- ◯ 1
- ◯ 2
- ◯ 3
- ◯ 4
- ◯ 5
- ◯ 6
- ◯ 7
- ◯ 8
- ◯ 9
- ◯ 10 Extremely important
- ◯ Prefer not to answer

For peer review only

Section 3/10: Your ethnic, cultural and national identity and background

30% complete

.

For peer review only

In which country did you gain your primary professional qualification?<br><br>(The qualification used for registration with your professional regulator, such as the GDC, GMC, GOC, GPhC, PSNI, HCPC, NMC)	◯ Not applicable - I do not hold a professional qualification<br>◯ UK<br>◯ Australia<br>◯ Bangladesh<br>◯ Bulgaria<br>◯ Canada<br>◯ China<br>◯ Czech Republic<br>◯ Egypt<br>◯ France<br>◯ Germany<br>◯ Ghana<br>◯ Greece<br>◯ Hungary<br>◯ India<br>◯ Iran<br>◯ Iraq<br>◯ Ireland<br>◯ Italy<br>◯ Jamaica<br>◯ Kenya<br>◯ Latvia<br>◯ Libya<br>◯ Lithuania<br>◯ Malaysia<br>◯ Malta<br>◯ Mauritius<br>◯ Myanmar<br>◯ Nepal<br>◯ Netherlands<br>◯ New Zealand<br>◯ Nigeria<br>◯ Pakistan<br>◯ Philippines<br>◯ Poland<br>◯ Portugal<br>◯ Romania<br>◯ Russia<br>◯ Slovakia<br>◯ Somalia<br>◯ South Africa<br>◯ Spain<br>◯ Sri Lanka<br>◯ Sudan<br>◯ Sweden<br>◯ Syria<br>◯ Trinidad and Tobago<br>◯ Turkey<br>◯ Uganda<br>◯ Ukraine<br>◯ USA<br>◯ Zimbabwe<br>◯ Other country (please specify)<br>◯ Prefer not to answer
Please specify the country in which you gained your primary professional qualification:	__________________________________
In which year did you obtain your primary professional qualification?	__________________________________<br>((Please enter in format YYYY, e.g. 1990))

For peer review only

What is the highest level of education you have completed?	◯ Primary (up to 11 years)<br>◯ Secondary (11 to 16 years)<br>◯ Post-secondary (16-18 years)<br>◯ Other qualifications below degree level (e.g. nursing diploma)<br>◯ Undergraduate degree or equivalent (e.g. BA, BSc, medical or nursing degree)<br>◯ Masters degree or equivalent (e.g. MSc, Ma)<br>◯ Doctorate level (e.g. PhD, MD)<br>◯ Prefer not to answer
What is the highest level of education your mother has completed?	◯ Primary (up to 11 years)<br>◯ Secondary (11 to 16 years)<br>◯ Post-secondary (16-18 years)<br>◯ Other qualifications below degree level (e.g. nursing diploma)<br>◯ Undergraduate degree or equivalent (e.g. BA, BSc, medical or nursing degree)<br>◯ Masters degree or equivalent (e.g. MSc, Ma)<br>◯ Doctorate level (e.g. PhD, MD)<br>◯ Prefer not to answer
What is the highest level of education your father has completed?	◯ Primary (up to 11 years)<br>◯ Secondary (11 to 16 years)<br>◯ Post-secondary (16-18 years)<br>◯ Other qualifications below degree level (e.g. nursing diploma)<br>◯ Undergraduate degree or equivalent (e.g. BA, BSc, medical or nursing degree)<br>◯ Masters degree or equivalent (e.g. MSc, Ma)<br>◯ Doctorate level (e.g. PhD, MD)<br>◯ Prefer not to answer

For peer review only

Section 3/10: Your ethnic, cultural and national identity and background

33% complete

.

In your current main job/role, what proportion of colleagues who are senior to you are of the same ethnic group as yourself?	◯ None<br>◯ Hardly any<br>◯ Some<br>◯ About a half<br>◯ Most<br>◯ Almost all<br>◯ All<br>◯ Prefer not to answer
In your current main job/role, what proportion of your colleagues who are senior to you are White?	◯ None<br>◯ Hardly any<br>◯ Some<br>◯ About a half<br>◯ Most<br>◯ Almost all<br>◯ All<br>◯ Prefer not to answer
Thinking about where you work in your current main job/role, does your organisation act fairly with regard to career progression / promotion, regardless of ethnic background, gender, religion, sexual orientation, disability or age?	◯ Not at all<br>◯ Rarely<br>◯ Sometimes<br>◯ Mostly<br>◯ Definitely<br>◯ Prefer not to answer

For peer review only

Section 4/10: Your home and family life

35% complete

.

This section asks about your home and the people who live with you, to help us to understand more about how things outside work may affect COVID-19 risk.

For peer review only

Section 4/10: Your home and family life

35% complete

.

The following questions may refer to your household.

A household is one person living alone, or a group of people (not necessarily related) living at the same address who share cooking facilities and share a living room, sitting room or dining area.

A household can consist of a single family, more than one family or no families in the case of a group of unrelated people.
Your household does not include anyone you may be in a support or childcare bubble with who live at a different address to you.

Do you have a “support bubble” (in England or Northern Ireland) or “extended household” (in Wales or Scotland) which includes people who usually live at a different address?	◯ No<br>◯ Yes<br>◯ Do not know<br>◯ Prefer not to answer
How many people are in this "support bubble"? Only count those who usually live at a different address.	______________________
Do you have a “childcare bubble” which includes people who usually live at a different address?<br><br>Do not include anyone already counted in the support bubble in the previous question.	◯ No<br>◯ Yes<br>◯ Do not know<br>◯ Prefer not to answer
How many people are in this "childcare bubble"?<br><br>Only count those who usually live at a different address.	______________________
Apart from you, how many other people are in your household?	◯ 0<br>◯ 1<br>◯ 2<br>◯ 3<br>◯ 4<br>◯ 5<br>◯ 6<br>◯ 7<br>◯ 8<br>◯ 9<br>◯ 10<br>◯ 11<br>◯ 12<br>◯ 13 or more people<br>◯ Prefer not to answer

For peer review only

Section 4/10: Your home and family life

40% complete

.

These questions are about each person living with you in your current household. We will ask you about each person in turn. It is your choice as to which order you put them in, but it might help to order them by age, from oldest to youngest.

Reminder: A household is one person living alone, or a group of people (not necessarily related) living at the same address who share cooking facilities and share a living room, sitting room or dining area.
A household can consist of a single family, more than one family, or no families in the case of a group of unrelated people.
Your household does not include anyone you may be in a support or childcare bubble with who live at a different address to you.

For peer review only

Section 4/10: Your home and family life

40% complete

.

Person 1 - What best describes this person's relationship to you? Please select from the list provided.

- ◯ Spouse/Partner
- ◯ Child
- ◯ Grandchild
- ◯ Parent
- ◯ Mother-in-law/Father-in-law/Your partner's parent
- ◯ Twin
- ◯ Sibling (not including your twin)
- ◯ Sister-in-law/Brother-in-law/Your partner's sibling
- ◯ Friend/Housemate
- ◯ Grandparent
- ◯ Aunt/Uncle/Your parent's sibling
- ◯ Great-Aunt/Great-Uncle/Your grandparent's sibling
- ◯ Cousin/Other familial relation
- ◯ Colleague
- ◯ Other
- ◯ Prefer not to answer

Person 1 - How old is this person?

- ◯ 0-1
- ◯ 2-4
- ◯ 5-10
- ◯ 11-16
- ◯ 17-18
- ◯ 19-24
- ◯ 25-34
- ◯ 35-44
- ◯ 45-54
- ◯ 55-64
- ◯ 65-74
- ◯ 75-84
- ◯ 85-94
- ◯ 95+
- ◯ Prefer not to answer

For peer review only

Section 4/10: Your home and family life

42% complete

.

Person 2 - What best describes this person's relationship to you?

- ◯ Spouse/Partner
- ◯ Child
- ◯ Grandchild
- ◯ Parent
- ◯ Mother-in-law/Father-in-law/Your partner's parent
- ◯ Twin
- ◯ Sibling (not including your twin)
- ◯ Sister-in-law/Brother-in-law/Your partner's sibling
- ◯ Friend/Housemate
- ◯ Grandparent
- ◯ Aunt/Uncle/Your parent's sibling
- ◯ Great-Aunt/Great-Uncle/Your grandparent's sibling
- ◯ Cousin/Other familial relation
- ◯ Colleague
- ◯ Other
- ◯ Prefer not to answer

Person 2 - How old is this person?

- ◯ 0-1
- ◯ 2-4
- ◯ 5-10
- ◯ 11-16
- ◯ 17-18
- ◯ 19-24
- ◯ 25-34
- ◯ 35-44
- ◯ 45-54
- ◯ 55-64
- ◯ 65-74
- ◯ 75-84
- ◯ 85-94
- ◯ 95+
- ◯ Prefer not to answer

For peer review only

Section 4/10: Your home and family life

44% complete

.

Person 3 - What best describes this person's relationship to you? Please select from the list provided.

- ◯ Spouse/Partner
- ◯ Child
- ◯ Grandchild
- ◯ Parent
- ◯ Mother-in-law/Father-in-law/Your partner's parent
- ◯ Twin
- ◯ Sibling (not including your twin)
- ◯ Sister-in-law/Brother-in-law/Your partner's sibling
- ◯ Friend/Housemate
- ◯ Grandparent
- ◯ Aunt/Uncle/Your parent's sibling
- ◯ Great-Aunt/Great-Uncle/Your grandparent's sibling
- ◯ Cousin/Other familial relation
- ◯ Colleague
- ◯ Other
- ◯ Prefer not to answer

Person 3 - How old is this person?

- ◯ 0-1
- ◯ 2-4
- ◯ 5-10
- ◯ 11-16
- ◯ 17-18
- ◯ 19-24
- ◯ 25-34
- ◯ 35-44
- ◯ 45-54
- ◯ 55-64
- ◯ 65-74
- ◯ 75-84
- ◯ 85-94
- ◯ 95+
- ◯ Prefer not to answer

For peer review only

Section 4/10: Your home and family life

46% complete

.

Person 4 - What best describes this person's relationship to you? Please select from the list provided.

- ◯ Spouse/Partner
- ◯ Child
- ◯ Grandchild
- ◯ Parent
- ◯ Mother-in-law/Father-in-law/Your partner's parent
- ◯ Twin
- ◯ Sibling (not including your twin)
- ◯ Sister-in-law/Brother-in-law/Your partner's sibling
- ◯ Friend/Housemate
- ◯ Grandparent
- ◯ Aunt/Uncle/Your parent's sibling
- ◯ Great-Aunt/Great-Uncle/Your grandparent's sibling
- ◯ Cousin/Other familial relation
- ◯ Colleague
- ◯ Other
- ◯ Prefer not to answer

Person 4 - How old is this person?

- ◯ 0-1
- ◯ 2-4
- ◯ 5-10
- ◯ 11-16
- ◯ 17-18
- ◯ 19-24
- ◯ 25-34
- ◯ 35-44
- ◯ 45-54
- ◯ 55-64
- ◯ 65-74
- ◯ 75-84
- ◯ 85-94
- ◯ 95+
- ◯ Prefer not to answer

For peer review only

Section 4/10: Your home and family life

47% complete

.

Person 5 - What best describes this person's relationship to you? Please select from the list provided.

- ◯ Spouse/Partner
- ◯ Child
- ◯ Grandchild
- ◯ Parent
- ◯ Mother-in-law/Father-in-law/Your partner's parent
- ◯ Twin
- ◯ Sibling (not including your twin)
- ◯ Sister-in-law/Brother-in-law/Your partner's sibling
- ◯ Friend/Housemate
- ◯ Grandparent
- ◯ Aunt/Uncle/Your parent's sibling
- ◯ Great-Aunt/Great-Uncle/Your grandparent's sibling
- ◯ Cousin/Other familial relation
- ◯ Colleague
- ◯ Other
- ◯ Prefer not to answer

Person 5 - How old is this person?

- ◯ 0-1
- ◯ 2-4
- ◯ 5-10
- ◯ 11-16
- ◯ 17-18
- ◯ 19-24
- ◯ 25-34
- ◯ 35-44
- ◯ 45-54
- ◯ 55-64
- ◯ 65-74
- ◯ 75-84
- ◯ 85-94
- ◯ 95+
- ◯ Prefer not to answer

For peer review only

Section 4/10: Your home and family life

49% complete

.

Person 6 - What best describes this person's relationship to you? Please select from the list provided.

- ◯ Spouse/Partner
- ◯ Child
- ◯ Grandchild
- ◯ Parent
- ◯ Mother-in-law/Father-in-law/Your partner's parent
- ◯ Twin
- ◯ Sibling (not including your twin)
- ◯ Sister-in-law/Brother-in-law/Your partner's sibling
- ◯ Friend/Housemate
- ◯ Grandparent
- ◯ Aunt/Uncle/Your parent's sibling
- ◯ Great-Aunt/Great-Uncle/Your grandparent's sibling
- ◯ Cousin/Other familial relation
- ◯ Colleague
- ◯ Other
- ◯ Prefer not to answer

Person 6 - How old is this person?

- ◯ 0-1
- ◯ 2-4
- ◯ 5-10
- ◯ 11-16
- ◯ 17-18
- ◯ 19-24
- ◯ 25-34
- ◯ 35-44
- ◯ 45-54
- ◯ 55-64
- ◯ 65-74
- ◯ 75-84
- ◯ 85-94
- ◯ 95+
- ◯ Prefer not to answer

For peer review only

Section 4/10: Your home and family life

51% complete

.

Person 7 - What best describes this person's relationship to you? Please select from the list provided.	◯ Spouse/Partner<br>◯ Child<br>◯ Grandchild<br>◯ Parent<br>◯ Mother-in-law/Father-in-law/Your partner's parent<br>◯ Twin<br>◯ Sibling (not including your twin)<br>◯ Sister-in-law/Brother-in-law/Your partner's sibling<br>◯ Friend/Housemate<br>◯ Grandparent<br>◯ Aunt/Uncle/Your parent's sibling<br>◯ Great-Aunt/Great-Uncle/Your grandparent's sibling<br>◯ Cousin/Other familial relation<br>◯ Colleague<br>◯ Other<br>◯ Prefer not to answer
Person 7 - How old is this person?	◯ 0-1<br>◯ 2-4<br>◯ 5-10<br>◯ 11-16<br>◯ 17-18<br>◯ 19-24<br>◯ 25-34<br>◯ 35-44<br>◯ 45-54<br>◯ 55-64<br>◯ 65-74<br>◯ 75-84<br>◯ 85-94<br>◯ 95+<br>◯ Prefer not to answer

For peer review only

Section 4/10: Your home and family life

53% complete

.

Person 8 - What best describes this person's relationship to you? Please select from the list provided.	◯ Spouse/Partner<br>◯ Child<br>◯ Grandchild<br>◯ Parent<br>◯ Mother-in-law/Father-in-law/Your partner's parent<br>◯ Twin<br>◯ Sibling (not including your twin)<br>◯ Sister-in-law/Brother-in-law/Your partner's sibling<br>◯ Friend/Housemate<br>◯ Grandparent<br>◯ Aunt/Uncle/Your parent's sibling<br>◯ Great-Aunt/Great-Uncle/Your grandparent's sibling<br>◯ Cousin/Other familial relation<br>◯ Colleague<br>◯ Other<br>◯ Prefer not to answer
Person 8 - How old is this person?	◯ 0-1<br>◯ 2-4<br>◯ 5-10<br>◯ 11-16<br>◯ 17-18<br>◯ 19-24<br>◯ 25-34<br>◯ 35-44<br>◯ 45-54<br>◯ 55-64<br>◯ 65-74<br>◯ 75-84<br>◯ 85-94<br>◯ 95+<br>◯ Prefer not to answer

For peer review only

Section 4/10: Your home and family life

54% complete

.

Person 9 - What best describes this person's relationship to you? Please select from the list provided.

- ◯ Spouse/Partner
- ◯ Child
- ◯ Grandchild
- ◯ Parent
- ◯ Mother-in-law/Father-in-law/Your partner's parent
- ◯ Twin
- ◯ Sibling (not including your twin)
- ◯ Sister-in-law/Brother-in-law/Your partner's sibling
- ◯ Friend/Housemate
- ◯ Grandparent
- ◯ Aunt/Uncle/Your parent's sibling
- ◯ Great-Aunt/Great-Uncle/Your grandparent's sibling
- ◯ Cousin/Other familial relation
- ◯ Colleague
- ◯ Other
- ◯ Prefer not to answer

Person 9 - How old is this person?

- ◯ 0-1
- ◯ 2-4
- ◯ 5-10
- ◯ 11-16
- ◯ 17-18
- ◯ 19-24
- ◯ 25-34
- ◯ 35-44
- ◯ 45-54
- ◯ 55-64
- ◯ 65-74
- ◯ 75-84
- ◯ 85-94
- ◯ 95+
- ◯ Prefer not to answer

For peer review only

Section 4/10: Your home and family life

56% complete

.

Person 10 - What best describes this person's relationship to you? Please select from the list provided.	◯ Spouse/Partner<br>◯ Child<br>◯ Grandchild<br>◯ Parent<br>◯ Mother-in-law/Father-in-law/Your partner's parent<br>◯ Twin<br>◯ Sibling (not including your twin)<br>◯ Sister-in-law/Brother-in-law/Your partner's sibling<br>◯ Friend/Housemate<br>◯ Grandparent<br>◯ Aunt/Uncle/Your parent's sibling<br>◯ Great-Aunt/Great-Uncle/Your grandparent's sibling<br>◯ Cousin/Other familial relation<br>◯ Colleague<br>◯ Other<br>◯ Prefer not to answer
Person 10 - How old is this person?	◯ 0-1<br>◯ 2-4<br>◯ 5-10<br>◯ 11-16<br>◯ 17-18<br>◯ 19-24<br>◯ 25-34<br>◯ 35-44<br>◯ 45-54<br>◯ 55-64<br>◯ 65-74<br>◯ 75-84<br>◯ 85-94<br>◯ 95+<br>◯ Prefer not to answer

For peer review only

Section 4/10: Your home and family life

58% complete

.

Person 11 - What best describes this person's relationship to you? Please select from the list provided.

- ◯ Spouse/Partner
- ◯ Child
- ◯ Grandchild
- ◯ Parent
- ◯ Mother-in-law/Father-in-law/Your partner's parent
- ◯ Twin
- ◯ Sibling (not including your twin)
- ◯ Sister-in-law/Brother-in-law/Your partner's sibling
- ◯ Friend/Housemate
- ◯ Grandparent
- ◯ Aunt/Uncle/Your parent's sibling
- ◯ Great-Aunt/Great-Uncle/Your grandparent's sibling
- ◯ Cousin/Other familial relation
- ◯ Colleague
- ◯ Other
- ◯ Prefer not to answer

Person 11 - How old is this person?

- ◯ 0-1
- ◯ 2-4
- ◯ 5-10
- ◯ 11-16
- ◯ 17-18
- ◯ 19-24
- ◯ 25-34
- ◯ 35-44
- ◯ 45-54
- ◯ 55-64
- ◯ 65-74
- ◯ 75-84
- ◯ 85-94
- ◯ 95+
- ◯ Prefer not to answer

For peer review only

Section 4/10: Your home and family life

60% complete

.

Person 12 - What best describes this person's relationship to you? Please select from the list provided.

- ◯ Spouse/Partner
- ◯ Child
- ◯ Grandchild
- ◯ Parent
- ◯ Mother-in-law/Father-in-law/Your partner's parent
- ◯ Twin
- ◯ Sibling (not including your twin)
- ◯ Sister-in-law/Brother-in-law/Your partner's sibling
- ◯ Friend/Housemate
- ◯ Grandparent
- ◯ Aunt/Uncle/Your parent's sibling
- ◯ Great-Aunt/Great-Uncle/Your grandparent's sibling
- ◯ Cousin/Other familial relation
- ◯ Colleague
- ◯ Other
- ◯ Prefer not to answer

Person 12 - How old is this person?

- ◯ 0-1
- ◯ 2-4
- ◯ 5-10
- ◯ 11-16
- ◯ 17-18
- ◯ 19-24
- ◯ 25-34
- ◯ 35-44
- ◯ 45-54
- ◯ 55-64
- ◯ 65-74
- ◯ 75-84
- ◯ 85-94
- ◯ 95+
- ◯ Prefer not to answer

For peer review only

Section 4/10: Your home and family life

62% complete

.

If you live with more than 12 people, please state the relationship to you and ages for the others in this box separated by a comma, e.g.:

Aunt 56, Colleague 25

For peer review only

Section 4/10: Your home and family life

62% complete

.

Apart from yourself, how many people in your household travel to work using public transport?

____
(Enter the number of people)

Apart from yourself, how many people in your household work in jobs that often bring them into close physical contact (within 2 metres) with others?

____
(Enter the number of people)

Some examples include: bus driver, carer, cleaner, doctor, supermarket checkout worker, teacher.

For peer review only

In which year did you move to your current address?

◯ 2021
◯ 2020
◯ 2019
◯ 2018
◯ 2017
◯ 2016
◯ 2015
◯ 2014
◯ 2013
◯ 2012
◯ 2011
◯ 2010
◯ 2009
◯ 2008
◯ 2007
◯ 2006
◯ 2005
◯ 2004
◯ 2003
◯ 2002
◯ 2001
◯ 2000
◯ 1999
◯ 1998
◯ 1997
◯ 1996
◯ 1995
◯ 1994
◯ 1993
◯ 1992
◯ 1991
◯ 1990
◯ 1989
◯ 1988
◯ 1987
◯ 1986
◯ 1985
◯ 1984
◯ 1983
◯ 1982
◯ 1981
◯ 1980
◯ 1979
◯ 1978
◯ 1977
◯ 1976
◯ 1975
◯ 1974
◯ 1973
◯ 1972
◯ 1971
◯ 1970
◯ 1969
◯ 1968
◯ 1967
◯ 1966
◯ 1965
◯ 1964
◯ 1963
◯ 1962
◯ 1961
◯ 1960
◯ 1959
◯ 1958
◯ 1957
◯ 1956
◯ 1955
◯ 1954
◯ 1953

For peer review only

	◯ 1952<br>◯ 1951<br>◯ 1950<br>◯ 1949<br>◯ 1948<br>◯ 1947<br>◯ 1946<br>◯ 1945<br>◯ 1944<br>◯ 1943<br>◯ 1942<br>◯ 1941<br>◯ 1940<br>◯ 1939<br>◯ 1938<br>◯ 1937<br>◯ 1936<br>◯ 1935<br>◯ 1934<br>◯ 1933<br>◯ 1932<br>◯ 1931<br>◯ 1930<br>◯ 1929<br>◯ 1928<br>◯ 1927<br>◯ 1926<br>◯ 1925<br>◯ 1924<br>◯ 1923<br>◯ 1922<br>◯ 1921<br>◯ 1920<br>◯ Prefer not to answer
In which month did you move to your current address?	◯ Jan<br>◯ Feb<br>◯ Mar<br>◯ Apr<br>◯ May<br>◯ Jun<br>◯ Jul<br>◯ Aug<br>◯ Sep<br>◯ Oct<br>◯ Nov<br>◯ Dec<br>◯ Prefer not to answer
What type of accommodation are you currently living in?	◯ Detached house<br>◯ Semi-detached house<br>◯ Terraced house<br>◯ Flat or apartment<br>◯ Hostel<br>◯ Mobile home or caravan<br>◯ Sheltered house<br>◯ Homeless<br>◯ Other (please specify)<br>◯ Prefer not to answer
Please specify what type of accommodation you live in:	________________________________

For peer review only

Is your current accommodation provided by or linked to your employer, e.g. hospital staff accommodation?	◯ No<br>◯ Yes<br>◯ Prefer not to answer
How many rooms are in your accommodation (not including the kitchen and bathroom(s))?	__________________________
Do you share any of the following rooms with people you do not consider to be a part of your household? You may select more than one answer. If you do not share any of the rooms listed, please select 'None'.	☐ None<br>☐ Kitchen<br>☐ Bathroom<br>☐ Living room, sitting room or dining area<br>☐ Prefer not to answer
Does your accommodation include shared communal areas such as hallways, stairwells or lifts?	◯ No<br>◯ Yes<br>◯ Prefer not to answer
Does your accommodation have a safe outdoor space (e.g., a garden or yard) where you can exercise or relax?	◯ No<br>◯ Yes<br>◯ Prefer not to answer
Is your garden/yard shared with other households or private?	◯ Shared<br>◯ Private<br>◯ Prefer not to answer

For peer review only

Section 5/10: Your friends and social network

63% complete

·

This section asks questions about your friends, social relationships and social networks.

For peer review only

Section 5/10: Your friends and social network

63% complete

.

How many people outside those in your household did you talk with in the last week (but not as a part of your work)
If you are answering this questionnaire on a smartphone, you may find it easier to view by rotating the screen.

	0	1-5	6-20	21-50	51+	Prefer not to answer
Remotely (e.g. over the phone, social media or via video media)	◯	◯	◯	◯	◯	◯
Face-to-face with social distancing	◯	◯	◯	◯	◯	◯
With physical contact (e.g. handshake/hug/kiss, etc)	◯	◯	◯	◯	◯	◯

What proportion of your friends are of the same ethnic group as yourself?

◯ None
◯ Hardly any
◯ Some
◯ About a half
◯ Most
◯ Almost all
◯ All
◯ Prefer not to answer

For peer review only

Section 6/10: Harassment and discrimination

64% complete
.

People from any background can be harassed or discriminated against for many reasons.

This section asks about your experiences of discrimination and harassment. Depending on your experiences, you may find some of the questions upsetting or difficult. You can stop at any time and return later if you wish, or you can choose not to answer a question.

You can find information about sources of support on our website and from the organisations below:
• Victim Support – support for people affected by crime or traumatic events, including hate crime: https://www.victimsupport.org.uk/
• Samaritans – Emotional support for everyone: www.samaritans.org
• Mind – Advice and support for anyone with a mental health problem: www.mind.org.uk

For peer review only

Section 6/10: Harassment and discrimination

64% complete

.

In your day-to-day life, how often do any of the following things happen to you?

If you are answering this questionnaire on a smartphone, you may find it easier to view by rotating the screen.

	Almost everyday	At least once a week	A few times a month	A few times a year	Less than once a year	Never	Prefer not to answer
You are treated with less courtesy than other people are.	○	○	○	○	○	○	○
You are treated with less respect than other people are.	○	○	○	○	○	○	○
You receive poorer service than other people at restaurants or shops.	○	○	○	○	○	○	○
People act as if they think you are not smart.	○	○	○	○	○	○	○
People act as if they are afraid of you.	○	○	○	○	○	○	○
People act as if they think you are dishonest.	○	○	○	○	○	○	○
People act as if they're better than you are.	○	○	○	○	○	○	○
You are called names or insulted.	○	○	○	○	○	○	○
You are threatened or harassed.	○	○	○	○	○	○	○

For peer review only

What do you think are the reasons for these experiences?
Please select all that apply.

- ☐ Your national origins
- ☐ Your gender
- ☐ Your ethnicity
- ☐ Your age
- ☐ Your religion
- ☐ Your height
- ☐ Your weight
- ☐ Your health or disability
- ☐ Your dress
- ☐ Some other aspect of your physical appearance
- ☐ Your sexual orientation
- ☐ Your education or income level
- ☐ Your language or accent
- ☐ Your social class
- ☐ Other (please specify)
- ☐ Prefer not to answer

Please specify what you think is the main reason for these experiences: __________________________________

For peer review only - http://bmjopen.bmj.com/site/about/guidelines.xhtml

In the last 12 months have you personally experienced discrimination at work from any of the following? Select all that apply.	☐ Patients / service users, their relatives or other members of the public<br>☐ Manager / team leader or other colleagues<br>☐ I have not experienced discrimination at work in the last 12 months<br>☐ I have not worked in the last 12 months<br>☐ Prefer not to answer
On what grounds have you experienced discrimination at work?	☐ Your national origins<br>☐ Your gender<br>☐ Your ethnicity<br>☐ Your age<br>☐ Your religion<br>☐ Your height<br>☐ Your weight<br>☐ Your health or disability<br>☐ Your dress<br>☐ Some other aspect of your physical appearance<br>☐ Your sexual orientation<br>☐ Your education or income level<br>☐ Your language or accent<br>☐ Your social class<br>☐ Other (please specify)<br>☐ Prefer not to answer
Please specify the grounds on which you have experienced discrimination at work:	__________________________________
Did you make a complaint about the discrimination at work?	○ No<br>○ No - but I did consider it<br>○ Yes - informally<br>○ Yes, I made a formal complaint<br>○ Prefer not to answer

For peer review only

Section 7/10: Your health

67% complete
·

This section asks about your overall physical and mental health. It will ask about:

• Height and weight
• Cigarette, vaping and alcohol usage
• Exercise and physical activity
• Overall health and specific health problems
• Mental health issues

For peer review only

For peer review only - http://bmjopen.bmj.com/site/about/guidelines.xhtml

Section 7/10: Your health

67% complete

.

What is your current height?	◯ Enter height in centimetres<br>◯ Enter height in feet and inches<br>◯ Do not know<br>◯ Prefer not to answer
Please enter your current height to the nearest centimetre:	__________________________
Feet	__________________________
Inches	__________________________
What is your current weight?	◯ Enter weight in kilograms<br>◯ Enter weight in stones and pounds<br>◯ Do not know<br>◯ Prefer not to answer
Please enter your current weight in kilograms:	__________________________
Stones	__________________________
Pounds	__________________________

For peer review only

Section 7/10: Your health

68% complete

.

Do you or have you ever smoked tobacco?	◯ Never<br>◯ Ex-smoker<br>◯ Current smoker<br>◯ Prefer not to answer
Do you currently use an e-cigarette or vape?	◯ Yes<br>◯ No<br>◯ Prefer not to answer
How often do you have a drink containing alcohol?	◯ Never<br>◯ Monthly or less<br>◯ 2-4 times per month<br>◯ 2-3 times per week<br>◯ 4+ times per week<br>◯ Prefer not to answer
How many units of alcohol do you drink in a typical week?<br>If you are unsure, see the guide below.<br><br>Pint of standard strength (3.6%) lager/beer/cider<br>2 units<br>Pint of higher strength (5.2%) lager/beer/cider<br>3 units<br>Medium (175ml) glass of wine<br>2 units<br>Large (250ml) glass of wine<br>3 units<br>Bottle (275ml) of alcopop1<br>1.5 units<br>Single shot (25ml) of spirits (e.g. vodka, whisky, gin, rum)<br>1 unit	◯ 0<br>◯ 1-7<br>◯ 8-14<br>◯ 15-21<br>◯ 22-28<br>◯ 29-35<br>◯ 36-50<br>◯ 51+<br>◯ Prefer not to answer<br>(Select the number of units)

For peer review only

Section 7/10: Your health

71% complete

.

Think about a typical week at work over the past month. Please consider the type and amount of physical activity involved in your work.

Please select one option only.

- ◯ I spend most of my time at work sitting
- ◯ I spend most of my time at work standing or walking. However, my work does not require much intense physical effort
- ◯ My work involves definite physical effort including handling of heavy objects and use of tools
- ◯ My work involves vigorous physical activity including handling of very heavy objects
- ◯ I am not in employment
- ◯ Prefer not to answer

During the last week, about how many hours did you spend on each of the following activities?
If you are answering this questionnaire on a smartphone, you may find it easier to view by rotating the screen.

	None	Less than 1 hour	1 - 3 hours	3 hours +	Prefer not to answer
Physical exercise such as swimming, jogging, aerobics, football, tennis, gym workout etc.	◯	◯	◯	◯	◯
Cycling, including cycling to work and during leisure time	◯	◯	◯	◯	◯
Walking, including walking to work, shopping, for pleasure etc.	◯	◯	◯	◯	◯
Housework/Childcare	◯	◯	◯	◯	◯
Gardening/DIY	◯	◯	◯	◯	◯

How would you describe your usual walking pace?
Please select one option only.

- ◯ Slow pace
- ◯ Steady average pace
- ◯ Brisk pace
- ◯ Fast pace
- ◯ Prefer not to answer

For peer review only

Section 7/10: Your health

72% complete

.

Has your lifestyle changed since the beginning of the COVID-19 pandemic?

Select 'This has not changed' if an option does not apply, e.g. you still do not smoke.
If you are answering this questionnaire on a smartphone, you may find it easier to view by rotating the screen.

	I do this more often	This has not changed	I do this less often	Prefer not to answer
Smoking	○	○	○	○
Drinking alcohol	○	○	○	○
Eating healthy food	○	○	○	○
Physical activity (including walking and cycling)	○	○	○	○

For peer review only

Section 7/10: Your health

73% complete

.

Last year, in 2019, how many times did you have a consultation with your GP about your own health?	◯ 0<br>◯ 1<br>◯ 2<br>◯ 3-5<br>◯ 6-10<br>◯ 11-20<br>◯ 21+<br>◯ Prefer not to answer
Last year, in 2019, how many days did you spend as a hospital inpatient?	◯ 0<br>◯ 1<br>◯ 2<br>◯ 3-5<br>◯ 6-10<br>◯ 11-20<br>◯ 21+<br>◯ Prefer not to answer
Did you have a flu vaccine last winter (2019-2020)?	◯ No<br>◯ Yes<br>◯ Do not know<br>◯ Prefer not to answer
Have you had a flu vaccine for this winter (2020-2021)?	◯ No<br>◯ Yes<br>◯ Do not know<br>◯ Prefer not to answer
Have you been contacted by letter or text message to say you are at severe risk from COVID-19 due to an underlying health condition and should be shielding?	◯ No<br>◯ Yes<br>◯ Do not know<br>◯ Prefer not to answer
Do you currently take any of these medications/supplements?<br><br>Please select all that apply. If you do not take any of these, please select "None of these".	☐ Ibuprofen / Nurofen, any other type of non-steroidal anti-inflammatory<br>☐ Vitamin D<br>☐ ACE-inhibitor (e.g. ramipril, lisinopril)<br>☐ Sartan (e.g. losartan, valsartan, candesartan)<br>☐ Entresto (sucubitril/valsartan)<br>☐ Metformin<br>☐ None of these<br>☐ Prefer not to answer

For peer review only

Are you, or do you, currently have any of the following?

Please select all that apply. If none apply to you, please select "None of the above".

- ☐ Pregnant
- ☐ Organ transplant
- ☐ Diabetes (Type I or II)
- ☐ Heart disease or heart problems
- ☐ Hypertension
- ☐ Overweight
- ☐ Stroke
- ☐ Kidney disease
- ☐ Liver disease
- ☐ Anaemia
- ☐ Asthma
- ☐ Other lung condition such as COPD, bronchitis or emphysema
- ☐ Cancer
- ☐ Condition affecting the brain and nerves (e.g. Dementia, Parkinson's, Multiple Sclerosis)
- ☐ A weakened immune system or reduced ability to deal with infections (as a result of a disease or treatment)
- ☐ Depression
- ☐ Anxiety
- ☐ Psychiatric disorder
- ☐ None of the above
- ☐ Prefer not to answer

For peer review only

Section 7/10: Your health

74% complete

.

Some of the following questions ask about your mental health. We would like to reassure you that your answers will be treated in accordance with strict research governance procedures, and the study has been reviewed by the Brighton and Sussex Research Ethics Committee.

If do not wish to answer a question, then please click on ‘Prefer not to answer’, or leave it blank.

For help during a mental health crisis or emergency:

Call 999 or go to A&E now if your life is at risk, or if you do not feel you can keep yourself safe.

For free confidential advice or to talk about anything that's troubling you, no matter how difficult:
Call 116 123 to talk to Samaritans at any time of day or night, or email: jo@samaritans.org for a reply within 24 hours Text "SHOUT" to 85258 to contact the Shout Crisis Text Line, or text "YM" if you're under 19 You can find a local NHS urgent mental health helpline here Urgent sources of support are also summarised at this NHS webpage. Other sources of support include: the Covid-19 Workforce Wellbeing pages, the mental health charity Mind, and Victim Support for victims of crime.

For peer review only

74% complete

.

Please select the ONE option that best describes your health TODAY.

MOBILITY

◯ I have no problems in walking about
◯ I have slight problems in walking about
◯ I have moderate problems in walking about
◯ I have severe problems in walking about
◯ I am unable to walk about

© EuroQol Research Foundation. EQ-5D™ is a trade mark of the EuroQol Research Foundation. UK (English) v2.1

For peer review only

74% complete
.

Please select the ONE option that best describes your health TODAY.

◯ I have no problems washing or dressing myself
◯ I have slight problems washing or dressing myself
◯ I have moderate problems washing or dressing myself
◯ I have severe problems washing or dressing myself
◯ I am unable to wash or dress myself

SELF-CARE

© EuroQol Research Foundation. EQ-5D™ is a trade mark of the EuroQol Research Foundation. UK (English) v2.1

For peer review only

74% complete
.

Please select the ONE option that best describes your health TODAY.

USUAL ACTIVITIES (e.g. work, study, housework, family or leisure activities)

- ◯ I have no problems doing my usual activities
- ◯ I have slight problems doing my usual activities
- ◯ I have moderate problems doing my usual activities
- ◯ I have severe problems doing my usual activities
- ◯ I am unable to do my usual activities

© EuroQol Research Foundation. EQ-5D™ is a trade mark of the EuroQol Research Foundation. UK (English) v2.1

For peer review only

74% complete
.

Please select the ONE option that best describes your health TODAY.

PAIN / DISCOMFORT	◯ I have no pain or discomfort<br>◯ I have slight pain or discomfort<br>◯ I have moderate pain or discomfort<br>◯ I have severe pain or discomfort<br>◯ I have extreme pain or discomfort

© EuroQol Research Foundation. EQ-5D™ is a trade mark of the EuroQol Research Foundation. UK (English) v2.1

For peer review only

74% complete
.

Please select the ONE option that best describes your health TODAY.

ANXIETY / DEPRESSION

- ◯ I am not anxious or depressed
- ◯ I am slightly anxious or depressed
- ◯ I am moderately anxious or depressed
- ◯ I am severely anxious or depressed
- ◯ I am extremely anxious or depressed

© EuroQol Research Foundation. EQ-5D™ is a trade mark of the EuroQol Research Foundation. UK (English) v2.1

For peer review only

74% complete
.

We would like to know how good or bad your health is TODAY.

This scale is numbered from 0 to 100.
100 means the best health you can imagine.
0 means the worst health you can imagine.

Using the slider, please indicate how your health is TODAY.

0 The worst health you can imagine | 50 | 100 The best health you can imagine

(Place a mark on the scale above)

© EuroQol Research Foundation. EQ-5D™ is a trade mark of the EuroQol Research Foundation. UK (English) v2.1

For peer review only

Section 7/10: Your health

78% complete

.

Over the last 2 weeks, how often have you been bothered by any of the following problems?

If you are answering this questionnaire on a smartphone, you may find it easier to view by rotating the screen.

	Not at all	Several days	More than half the days	Nearly every day	Prefer not to answer
Feeling nervous, anxious or on edge?	○	○	○	○	○
Not being able to stop or control worrying?	○	○	○	○	○
Little interest or pleasure in doing things	○	○	○	○	○
Feeling down, depressed, or hopeless?	○	○	○	○	○

How worried are you about your future financial situation?

○ Not at all
○ A little bit
○ Moderately
○ Quite a bit
○ Extremely
○ Prefer not to answer

These questions are about problems and complaints that people sometimes have in response to stressful life experiences. Please indicate how much you have been bothered by each problem in the past month.
If you are answering this questionnaire on a smartphone, you may find it easier to view by rotating the screen.

	Not at all	A little bit	Moderately	Quite a bit	Extremely	Prefer not to answer
Repeated, disturbing memories, thoughts, or images of a stressful experience from the past?	○	○	○	○	○	○
Feeling very upset when something reminded you of a stressful experience from the past?	○	○	○	○	○	○
Avoided activities or situations because they reminded you of a stressful experience from the past?	○	○	○	○	○	○

For peer review only

Section 7/10: Your health

80% complete

.

The following statements describe how people sometimes feel. For each statement, please indicate how often you feel this way.
If you are answering this questionnaire on a smartphone, you may find it easier to view by rotating the screen.

	Hardly ever or never	Some of the time	Often	Prefer not to answer
How often do you feel you lack companionship?	◯	◯	◯	◯
How often do you feel left out?	◯	◯	◯	◯
How often do you feel isolated from others?	◯	◯	◯	◯

Overall, how satisfied are you with your life nowadays?

Please give an answer on a scale of 0 to 10, where 0 is 'not at all' and 10 is 'completely'.

◯ 0 Not at all satisfied
◯ 1
◯ 2
◯ 3
◯ 4
◯ 5
◯ 6
◯ 7
◯ 8
◯ 9
◯ 10 Completely satisfied
◯ Prefer not to answer

For peer review only

Section 7/10: Your health

This is the end of the questions about your mental health. We have repeated the information about sources of support from the beginning of this section here.
For help during a mental health crisis or emergency:

Call 999 or go to A&E now if your life is at risk, or if you do not feel you can keep yourself safe.

For free confidential advice or to talk about anything that's troubling you, no matter how difficult:
Call 116 123 to talk to Samaritans at any time of day or night, or email: jo@samaritans.org for a reply within 24 hours Text "SHOUT" to 85258 to contact the Shout Crisis Text Line, or text "YM" if you're under 19 You can find a local NHS urgent mental health helpline here Urgent sources of support are also summarised at this NHS webpage. Other sources of support include: the Covid-19 Workforce Wellbeing pages, the mental health charity Mind, and Victim Support for victims of crime.

For peer review only

Section 8/10: Your health and COVID-19

81% complete

·

This next section asks questions about COVID-19 and your health and your feelings about the pandemic. It will ask about:

What changes you made during the UK national lockdown COVID-19 tests and whether you have had COVID-19 The chances of catching COVID-19 The possible effects of COVID-19 on society Possible vaccines for COVID-19 Attitudes towards the COVID-19 pandemic

For peer review only

Section 8/10: Your health and COVID-19

81% complete

.

Have you been in close contact with anyone with COVID-19 outside of your work in the last two weeks?

- ◯ Yes, someone in my household had/has confirmed/tested COVID-19
- ◯ Yes, someone in my household had/has suspected COVID-19
- ◯ Yes, contact with a confirmed/tested COVID-19 case outside of my household
- ◯ Yes, contact with a suspected COVID-19 case outside of my household
- ◯ No, not to my knowledge
- ◯ Prefer not to answer

Thinking back to when COVID-19 was emerging from January 2020 until the beginning of the UK national lockdown on 23rd March 2020, which of the following activities were you doing then, and which have you been doing in the past few weeks?
Please select all that apply.

	Between January 2020and March 2020	Past few weeks	Prefer not to answer
Cancelling my usual social activities	☐	☐	☐
Not going to work	☐	☐	☐
Only going shopping for essential things	☐	☐	☐
Not going to a grocery store or pharmacy	☐	☐	☐
Not leaving the house	☐	☐	☐
Wearing a face mask outside my home	☐	☐	☐
Trying to avoid physical contact with people	☐	☐	☐
Following handwashing recommendations	☐	☐	☐
Using hand sanitiser more than usual	☐	☐	☐
Following coughing and sneezing recommendations	☐	☐	☐
Using tissues more than usual	☐	☐	☐
Wearing gloves while going out of my home	☐	☐	☐
Avoiding public transport	☐	☐	☐
Avoiding going to restaurants/bars/pubs	☐	☐	☐

For peer review only

Avoiding going for walks or exercise outside	☐	☐	☐
Avoiding taking my children out of my home (if applicable)	☐	☐	☐
Prefer not to answer	☐	☐	☐

Thinking back to the months of UK national lockdown which began on 23rd March, which of these is closest to your view?

- ◯ I enjoyed nothing about the UK national lockdown
- ◯ I enjoyed a few aspects of the UK national lockdown
- ◯ I enjoyed some aspects of the UK national lockdown
- ◯ I enjoyed most aspects of the UK national lockdown
- ◯ I enjoyed almost all of the UK national lockdown
- ◯ Prefer not to answer

For peer review only

Section 8/10: Your health and COVID-19

83% complete

.

Have you ever had a test to see if you have or have had COVID-19?<br>Select all that apply.	☐ No<br>☐ Yes, A swab test (swab of your throat and/or nose) which tests for active infection<br>☐ Yes, An antibody test for COVID-19 (a blood test, or a drop of blood from your finger) which tests for past infection<br>☐ Do not know<br>☐ Prefer not to answer
What was the reason that you had the swab test?<br>Please select all that apply.	☐ Because I had symptoms<br>☐ Because I have been in contact with someone who had COVID-19<br>☐ Because of my job<br>☐ Before going into hospital as a patient (e.g. for surgery)<br>☐ It was offered by my local council (e.g. posted through my door)<br>☐ For another reason (please specify)<br>☐ Prefer not to answer
Please specify the reason you had the swab test:	__________________________________
Have you ever had a positive result from a swab test (i.e. showing that you had coronavirus when the swab was taken)?	◯ No<br>◯ Yes<br>◯ Do not know<br>◯ Prefer not to answer
When was the sample taken for the test that came back positive?<br><br>Give the latest date if you have had more than one	__________________________________
Have you had a positive result from an antibody test (i.e. showing that you had coronavirus at some time previously)?	◯ No<br>◯ Yes<br>◯ Do not know<br>◯ Prefer not to answer
When was the sample taken for the test that came back positive?<br><br>Give the latest date if you have had more than one	__________________________________

For peer review only

Have you experienced any of the following symptoms in the past two weeks?<br><br>Please select all that apply.	☐ Fever<br>☐ Cough - dry<br>☐ Cough - mucus or phlegm<br>☐ Sore throat<br>☐ Chest tightness<br>☐ Shortness of breath<br>☐ Runny nose<br>☐ Nasal congestion<br>☐ Sneezing<br>☐ Muscle or body aches<br>☐ Fatigue<br>☐ Unusual loose motions or diarrhoea<br>☐ Vomiting<br>☐ Loss of smell<br>☐ Loss of taste<br>☐ Skin rash<br>☐ None of these<br>☐ Prefer not to answer
Do you think that you currently have or have had COVID-19?	◯ No<br>◯ Unsure<br>◯ Yes, my own suspicions<br>◯ Yes, suspected by a doctor but not tested<br>◯ Yes, confirmed by a positive test<br>◯ Prefer not to answer
When were you told/when did you first think you had COVID-19?	
Month	◯ Jan<br>◯ Feb<br>◯ Mar<br>◯ Apr<br>◯ May<br>◯ Jun<br>◯ Jul<br>◯ Aug<br>◯ Sep<br>◯ Oct<br>◯ Nov<br>◯ Dec<br>◯ Prefer not to answer
Year	◯ 2021<br>◯ 2020<br>◯ 2019<br>◯ Prefer not to answer
Were you hospitalised for treatment of your COVID-19 disease?	◯ No<br>◯ Yes<br>◯ Prefer not to answer
Were you admitted to the intensive care unit (ITU/ICU)?	◯ No<br>◯ Yes<br>◯ Prefer not to answer

For peer review only

How long have you been unwell since having COVID-19?	◯ Less than 3 weeks<br>◯ 3 - 5 weeks<br>◯ More than 5 weeks but less than 3 months<br>◯ More than 3 months but less than 6 months<br>◯ More than 6 months<br>◯ Prefer not to answer
Has any health professional told you that your symptoms are likely to be the ongoing effect of COVID-19?	◯ Yes<br>◯ No<br>◯ Prefer not to answer

For peer review only

Section 8/10: Your health and COVID-19

89% complete

.

How concerned are you that you will get COVID-19 and require hospitalisation?

- ◯ Not at all concerned
- ◯ A little concerned
- ◯ Quite concerned
- ◯ Very concerned
- ◯ Prefer not to answer

How concerned are you that you might unknowingly spread COVID-19 to others?

- ◯ Not at all concerned
- ◯ A little concerned
- ◯ Quite concerned
- ◯ Very concerned
- ◯ Prefer not to answer

Do you personally know anyone who has died from COVID-19 (not including patients you have cared for as part of your work)?

Select all that apply.

- ☐ Yes, family member(s)
- ☐ Yes, friend(s)
- ☐ Yes, colleague(s)
- ☐ Yes, someone else
- ☐ No
- ☐ Prefer not to answer

Where do you get information about COVID-19?

Select all that apply.

- ☐ Friends, family, neighbours
- ☐ Colleagues
- ☐ Employer or manager
- ☐ Television
- ☐ Radio
- ☐ Newspapers or magazines
- ☐ Government or NHS posters, adverts or leaflets
- ☐ Twitter
- ☐ Other social media (e.g. Facebook, Instagram)
- ☐ UK Government website
- ☐ Welsh, Scottish or NI government website
- ☐ NHS website
- ☐ WHO website
- ☐ Other websites
- ☐ Local council
- ☐ My own GP or other healthcare workers
- ☐ Scientific journals
- ☐ Not applicable
- ☐ Prefer not to answer

For peer review only

Section 8/10: Your health and COVID-19

92% complete

.

What do you think is your personal chance of catching the coronavirus in the next month?

Please enter a value on a scale from 0 to 100, where 0 means there is no possibility that you will and 100 means that you definitely will.

____

What do you think is your personal chance of catching the coronavirus in the next six months?

Please enter a value on a scale from 0 to 100, where 0 means there is no possibility that you will and 100 means that you definitely will.

____

If you do catch coronavirus, what do you think are your chances of needing hospital treatment?
Please enter a value on a scale from 0 to 100, where 0 means there is no possibility that you will and 100 means that you definitely will.

____

What percentage of people in the UK who are hospitalised with coronavirus do you think will end up dying as a result of the disease?
Please enter a value on a scale from 0 to 100, where 0 means nobody hospitalised will die and 100 means everybody hospitalised will die.

____

For peer review only

Section 8/10: Your health and COVID-19

93% complete

.

Have you taken part in a trial of a COVID-19 vaccine?

- ◯ Yes
- ◯ No, but I would if asked
- ◯ No, but I would not if asked
- ◯ Prefer not to answer

If yes, which one? __________________________________

The following question refers to any vaccine you have been offered or may be offered that is not as part of a COVID-19 vaccine trial.

Have you had, or are you going to have, a vaccination against COVID-19?

- ◯ I have already had at least one COVID-19 vaccination
- ◯ I have not had a vaccination but have been told that I will be offered a vaccination in the near future
- ◯ I have been offered a vaccination but have decided not to have the vaccine
- ◯ I have not yet been offered a vaccination but intend to have the vaccine when offered
- ◯ I have not yet been offered a vaccination but have decided not to have a vaccine when offered
- ◯ Prefer not to answer

How many doses have you had?

- ◯ 1
- ◯ 2
- ◯ Unsure
- ◯ Prefer not to answer

Was the vaccination:

- ◯ In a hospital
- ◯ In a care home
- ◯ From a GP
- ◯ Other
- ◯ Prefer not to answer

Please specify: __________________________________

What was the date when you had your first vaccination? (if you are unsure please give your best estimate) __________________________________

Which vaccine did you receive?

- ◯ Pfizer-Biontech
- ◯ Oxford-AstraZeneca
- ◯ Moderna
- ◯ Other
- ◯ Unsure
- ◯ Prefer not to answer

Please specify: __________________________________

For peer review only

Did you consider not having the vaccination?	◯ No<br>◯ Yes, I did consider not having the vaccination<br>◯ Prefer not to answer
What would have been your reason(s) for not having the vaccination? Please select all that apply.	☐ I have allergies, needle-phobia, am immuno-compromised, or have other clinical reasons not to be vaccinated<br>☐ I am concerned about the safety or potential side-effects of a COVID-19 vaccine<br>☐ I am not convinced that COVID-19 vaccines will be effective<br>☐ Vaccines may not have been tested thoroughly in all ethnic groups<br>☐ I have had COVID-19 and therefore do not feel I need the vaccine<br>☐ I am taking part in a clinical trial of a COVID-19 vaccine<br>☐ I would prefer one of the other COVID-19 vaccines that are being developed<br>☐ I would prefer to wait until many other people have received a COVID-19 vaccine<br>☐ I do not feel that I personally am at risk from COVID-19<br>☐ I would rather the vaccine were used for other people who need it more than I do<br>☐ I do not believe in vaccinations in general<br>☐ Other reason<br>☐ Prefer not to answer
Please specify:	__________________________________
When is the vaccination likely to be?	◯ In a few days<br>◯ In the next week<br>◯ In the next two weeks<br>◯ In the next month<br>◯ Other<br>◯ Prefer not to answer
Please specify:	__________________________________
Will this vaccination be:	◯ In a hospital<br>◯ In a care home<br>◯ From a GP<br>◯ Other<br>◯ Prefer not to answer
Please specify:	__________________________________
Are you considering not having the vaccination?	◯ No<br>◯ Yes, I am considering not having the vaccination<br>◯ Prefer not to answer

For peer review only

What might be your reason(s) for not having the vaccination? Please select all that apply.	☐ I have allergies, needle-phobia, am immuno-compromised, or have other clinical reasons not to be vaccinated<br>☐ I am concerned about the safety or potential side-effects of a COVID-19 vaccine<br>☐ I am not convinced that COVID-19 vaccines will be effective<br>☐ Vaccines may not have been tested thoroughly in all ethnic groups<br>☐ I have had COVID-19 and therefore do not feel I need the vaccine<br>☐ I am taking part in a clinical trial of a COVID-19 vaccine<br>☐ I would prefer one of the other COVID-19 vaccines that are being developed<br>☐ I would prefer to wait until many other people have received a COVID-19 vaccine<br>☐ I do not feel that I personally am at risk from COVID-19<br>☐ I would rather the vaccine were used for other people who need it more than I do<br>☐ I do not believe in vaccinations in general<br>☐ Other reason<br>☐ Prefer not to answer
Please specify:	__________________________________
Was the vaccination offered by:	◯ A hospital<br>◯ A care home<br>◯ A GP<br>◯ Other<br>◯ Prefer not to answer.
Please specify:	__________________________________

For peer review only

What were your reason(s) for not having the vaccination? Please select all that apply.	☐ I have allergies, needle-phobia, am immuno-compromised, or have other clinical reasons not to be vaccinated<br>☐ I am concerned about the safety or potential side-effects of a COVID-19 vaccine<br>☐ I am not convinced that COVID-19 vaccines will be effective<br>☐ Vaccines may not have been tested thoroughly in all ethnic groups<br>☐ I have had COVID-19 and therefore do not feel I need the vaccine<br>☐ I am taking part in a clinical trial of a COVID-19 vaccine<br>☐ I would prefer one of the other COVID-19 vaccines that are being developed<br>☐ I would prefer to wait until many other people have received a COVID-19 vaccine<br>☐ I do not feel that I personally am at risk from COVID-19<br>☐ I would rather the vaccine were used for other people who need it more than I do<br>☐ I do not believe in vaccinations in general<br>☐ Other reason<br>☐ Prefer not to answer
Please specify:	__________________________________
When you are offered the vaccine, is there anything that might make you consider not having it?	○ No<br>○ Yes, I would consider not having the vaccine<br>○ Prefer not to answer
What are your reason(s) for considering not having the vaccine? Please select all that apply.	☐ I have allergies, needle-phobia, am immuno-compromised, or have other clinical reasons not to be vaccinated<br>☐ I am concerned about the safety or potential side-effects of a COVID-19 vaccine<br>☐ I am not convinced that COVID-19 vaccines will be effective<br>☐ Vaccines may not have been tested thoroughly in all ethnic groups<br>☐ I have had COVID-19 and therefore do not feel I need the vaccine<br>☐ I am taking part in a clinical trial of a COVID-19 vaccine<br>☐ I would prefer one of the other COVID-19 vaccines that are being developed<br>☐ I would prefer to wait until many other people have received a COVID-19 vaccine<br>☐ I do not feel that I personally am at risk from COVID-19<br>☐ I would rather the vaccine were used for other people who need it more than I do<br>☐ I do not believe in vaccinations in general<br>☐ Other reason<br>☐ Prefer not to answer
Please specify:	__________________________________

For peer review only

What are your reason(s) for not having the vaccination? Please select all that apply.

- ☐ I have allergies, needle-phobia, am immuno-compromised, or have other clinical reasons not to be vaccinated
- ☐ I am concerned about the safety or potential side-effects of a COVID-19 vaccine
- ☐ I am not convinced that COVID-19 vaccines will be effective
- ☐ Vaccines may not have been tested thoroughly in all ethnic groups
- ☐ I have had COVID-19 and therefore do not feel I need the vaccine
- ☐ I am taking part in a clinical trial of a COVID-19 vaccine
- ☐ I would prefer one of the other COVID-19 vaccines that are being developed
- ☐ I would prefer to wait until many other people have received a COVID-19 vaccine
- ☐ I do not feel that I personally am at risk from COVID-19
- ☐ I would rather the vaccine were used for other people who need it more than I do
- ☐ I do not believe in vaccinations in general
- ☐ Other reason
- ☐ Prefer not to answer

Please specify: ______________________________

How much do you agree with the following statements about vaccinations in general?

	1 Strongly disagree	2	3	4	5 Strongly agree	Prefer not to answer
I can rely on vaccines to stop serious infectious diseases	○	○	○	○	○	○
Although most vaccines appear to be safe, there may be problems that we have not yet discovered	○	○	○	○	○	○
Authorities promote vaccination for financial gain, not for people's health	○	○	○	○	○	○
Being exposed to diseases naturally is safer for the immune system than being exposed through vaccination	○	○	○	○	○	○

When, if at all, do you think it will be possible to vaccinate most of the population against coronavirus?

- ○ 1 month from now
- ○ 2 months from now
- ○ 3 months from now
- ○ 6 months from now
- ○ 12 months from now
- ○ 18 months from now
- ○ 2 years from now
- ○ More than 2 years
- ○ Do not know
- ○ Prefer not to answer

For peer review only

Section 8/10: Your health and COVID-19

94% complete

.

The coronavirus pandemic will have many effects in the UK, five of which are shown below. How serious do you think each of them is in terms of its consequences?

Please select the relevant number next to the statement (i.e. if you believe "Effects on economy and jobs" to be the most serious then select '1' for this effect).

You will only be able to select each number once.

If you are answering this questionnaire on a smartphone, you may find it easier to view by rotating the screen.

	1 (Most serious)	2	3	4	5 (Least serious)	Prefer not to answer
Effects on children and their education	○	○	○	○	○	○
Effects on the economy and jobs	○	○	○	○	○	○
Increasing deaths as a direct result of catching coronavirus	○	○	○	○	○	○
Increased deaths due to fewer healthcare resources to identify and treat medical conditions other than coronavirus	○	○	○	○	○	○
Increased mental health issues	○	○	○	○	○	○

When a vaccine becomes available, would you wish to receive it?

○ Definitely Yes
○ Probably Yes
○ Probably No
○ Definitely No
○ Prefer not to answer

For peer review only

Section 8/10: Your health and COVID-19

96% complete
.

Do you think the coronavirus crisis will increase or decrease the level of inequality in the UK, compared with before the pandemic?

◯ Increase a lot ◯ Increase a bit
◯ Make no difference ◯ Decrease a bit
◯ Decrease a lot ◯ Prefer not to answer

To what extent do you think the following statements about COVID-19 are true or false? Give your immediate thoughts, and don't look up the answers.

If you are answering this questionnaire on a smartphone, you may find it easier to view by rotating the screen.

	Definitely true	Probably true	Probably false	Definitely false	Prefer not to answer
A person can be infected twice with coronavirus	◯	◯	◯	◯	◯
Coronavirus is less infectious than the influenza virus	◯	◯	◯	◯	◯
Coronavirus was created in a laboratory	◯	◯	◯	◯	◯
Infection with coronavirus is equally likely in men and women	◯	◯	◯	◯	◯
Mortality from coronavirus is higher in men than women	◯	◯	◯	◯	◯
Most people in the UK have already had coronavirus without realising it	◯	◯	◯	◯	◯
The current pandemic is part of a global effort to force everyone to be vaccinated whether they want to or not	◯	◯	◯	◯	◯
The genetic material in a coronavirus is RNA, unlike that of humans which is DNA	◯	◯	◯	◯	◯
The number of people reported as dying from coronavirus is being deliberately reduced or hidden by the authorities	◯	◯	◯	◯	◯
The symptoms that most people blame on coronavirus appear to be linked to 5G network radiation	◯	◯	◯	◯	◯
There is no hard evidence that coronavirus really exists	◯	◯	◯	◯	◯

For peer review only

Section 9/10: Your approach to life in general

96% complete
.

People differ in many ways. The questions below cover a range of ways that people can differ from one another, in terms of values, attitudes, and approach to life in general.
For most of the questions there is no right or wrong answer. Do not think too hard about each answer but instead give the one that most immediately seems correct for you, being as honest as you can.

For peer review only

Section 9/10: Your approach to life in general

96% complete

.

The following questions are about how you see yourself as a person.

Please select the number which best describes how you see yourself where 1 means 'does not apply to me at all' and 7 means 'applies to me perfectly'.

I see myself as someone who...

If you are answering this questionnaire on a smartphone, you may find it easier to view by rotating the screen.

	Does not apply to me at all (1)	2	3	4	5	6	Applies to me perfectly (7)	Prefer not to answer
Is sometimes rude to others	○	○	○	○	○	○	○	○
Does a thorough job	○	○	○	○	○	○	○	○
Is talkative	○	○	○	○	○	○	○	○
Worries a lot	○	○	○	○	○	○	○	○
Is original, comes up with new ideas	○	○	○	○	○	○	○	○
Has a forgiving nature	○	○	○	○	○	○	○	○
Tends to be lazy	○	○	○	○	○	○	○	○
Is outgoing, sociable	○	○	○	○	○	○	○	○
Gets nervous easily	○	○	○	○	○	○	○	○
Values artistic, aesthetic experiences	○	○	○	○	○	○	○	○
Is considerate and kind to almost everyone	○	○	○	○	○	○	○	○
Does things efficiently	○	○	○	○	○	○	○	○
Is reserved	○	○	○	○	○	○	○	○
Is relaxed, handles stress well	○	○	○	○	○	○	○	○
Has an active imagination	○	○	○	○	○	○	○	○

For peer review only

Section 9/10: Your approach to life in general

97% complete

.

For each of the following statements, indicate the extent to which you agree or disagree by selecting a number from 1 (Strongly disagree) to 7 (strongly agree).
If you are answering this questionnaire on a smartphone, you may find it easier to view by rotating the screen.

	1 (Strongly disagree)	2	3	4	5	6	7 (Strongly agree)	Prefer not to answer
My life is determined by my own actions	○	○	○	○	○	○	○	○
I am usually able to protect my personal interests	○	○	○	○	○	○	○	○
I can pretty much determine what will happen in my life	○	○	○	○	○	○	○	○
To a great extent, my life is controlled by accidental happenings	○	○	○	○	○	○	○	○
Often there is no chance of protecting my personal interest from bad luck happenings	○	○	○	○	○	○	○	○
When I get what I want, it's usually because I'm lucky	○	○	○	○	○	○	○	○
People like myself have very little chance of protecting our personal interests where they conflict with those of strong pressure groups	○	○	○	○	○	○	○	○
My life is chiefly controlled by powerful others	○	○	○	○	○	○	○	○
I feel like what happens in my life is mostly determined by powerful people	○	○	○	○	○	○	○	○
If someone is meant to have a serious disease, they will get that disease.	○	○	○	○	○	○	○	○
My health is determined by fate.	○	○	○	○	○	○	○	○
My health is determined by something greater than myself.	○	○	○	○	○	○	○	○
I will stay healthy if I am lucky.	○	○	○	○	○	○	○	○
I like having a clear and structured mode of life	○	○	○	○	○	○	○	○

For peer review only

I would quickly become impatient and irritated if I could not find a solution to a problem immediately	◯	◯	◯	◯	◯	◯	◯	◯
Genes are more important than one's own behaviour in determining one's health	◯	◯	◯	◯	◯	◯	◯	◯

For peer review only

Section 9/10: Your approach to life in general

98% complete

.

How do you see yourself: are you generally a person who is fully prepared to take risks or do you try to avoid taking risks?

Please select a number on the scale, where the value 0 means: 'not at all willing to take risks' and the value 10 means: 'very willing to take risks'.

- ◯ 0 Not at all willing to take risks
- ◯ 1
- ◯ 2
- ◯ 3
- ◯ 4
- ◯ 5
- ◯ 6
- ◯ 7
- ◯ 8
- ◯ 9
- ◯ 10 Very willing to take risks
- ◯ Prefer not to answer

Think back to the work you were doing at the end of 2019 or in early 2020 before the UK national lockdown on 23rd March 2020. How well do the following statements describe your typical work environment at that time?

If you had more than one job, think about your main job/role. If you were not working please select “not applicable”.

If you are answering this questionnaire on a smartphone, you may find it easier to view by rotating the screen.

	Definitely disagree	Somewhat disagree	Somewhat agree	Definitely agree	Not applicable	Prefer not to answer
There was a real opportunity for me to choose the particular things I worked on	◯	◯	◯	◯	◯	◯
My work colleagues really tried hard to get to know one another	◯	◯	◯	◯	◯	◯
I had a lot of choice about the work I did	◯	◯	◯	◯	◯	◯
I was required to do too many different things	◯	◯	◯	◯	◯	◯
My coworkers were supportive and friendly towards me	◯	◯	◯	◯	◯	◯
There seemed to be too much work to get through	◯	◯	◯	◯	◯	◯

Think back to the work you were doing at the end of 2019 or early 2020 before the UK national lockdown on 23rd March 2020. How well do the following statements describe overall the way you felt about your work at that time?

If you had more than one job, think about your main job/role. If you were not working please select “Not applicable”.

If you are answering this questionnaire on a smartphone, you may find it easier to view by rotating the screen.

For peer review only

	Never	A few times a year	Once a month or less	A few times a month	Once a week	A few times a week	Every day	Not applicable	Prefer not to answer
I was exhausted in the morning at the thought of another day at work	◯	◯	◯	◯	◯	◯	◯	◯	◯
When I was working I forgot everything else around me	◯	◯	◯	◯	◯	◯	◯	◯	◯
I didn't have enough energy for family and friends during leisure time	◯	◯	◯	◯	◯	◯	◯	◯	◯
I was proud of the work that I did	◯	◯	◯	◯	◯	◯	◯	◯	◯
At work I felt bursting with energy	◯	◯	◯	◯	◯	◯	◯	◯	◯

For peer review only

Section 10/10: A few last questions

99% complete
.

The questionnaire is now almost finished, and we are very grateful to you for having completed it so far and having contributed to the UK-REACH study.
People filling in questionnaires often feel that although they have answered a lot of questions they often have not had the chance to say what they really feel about issues that have come up. There are therefore three open-ended questions on very broad issues, to do with:

- Why ethnic minorities might be more vulnerable to COVID-19
- How society might change as a result of COVID-19
- How your own future might change as a result of COVID-19

Only if you want to, please type whatever you want to say into the three boxes. You should have more than enough space, and the computer will tell you how much space you have left. Often open-ended comments such as these can provide much of interest to researchers.
Finally, there are two short questions asking for a little feedback on the questionnaire itself, about whether you think it might be useful and whether you think it was far too long.

What are your thoughts on why people from ethnic minorities working in health and care have been more severely affected by COVID-19? ________________________

How do you see society changing as a result of COVID-19? ________________________

How do you see your own future changing as a result of COVID-19? ________________________

Two very last questions:

We know that the questionnaire was long. Did you think:

- ◯ It should have asked about a lot more things
- ◯ It should have asked about some more things
- ◯ It was about the right length
- ◯ It should have been shorter
- ◯ It should have been much shorter

How useful do you think this questionnaire might be for researching and understanding COVID-19 in ethnic minorities?

- ◯ Not at all useful
- ◯ Not very useful
- ◯ Fairly useful
- ◯ Very useful
- ◯ Extremely useful

Thank you very much for your assistance in this study, which is very much appreciated.

Information about the progress of the study and the research findings will be placed on the UK-REACH website.
If you are affected by any of the issues raised in this questionnaire or are looking for information on COVID-19 (coronavirus) please visit:
• Government guidelines: www.gov.uk/coronavirus
• NHS advice: https://www.nhs.uk/conditions/coronavirus-covid-19/symptoms/
• NHS – Where to get urgent help for mental health:
https://www.nhs.uk/using-the-nhs/nhs-services/mental-health-services/where-to-get-urgent-help-for-mental-health/
• Covid-19 Workforce Wellbeing: https://www.practitionerhealth.nhs.uk/covid-19-workforce-wellbeing
• Victim Support – support for people affected by crime or traumatic events, including hate crime:
https://www.victimsupport.org.uk/
• Samaritans – Emotional support for everyone: www.samaritans.org
• Mind – Advice and support for anyone with a mental health problem: www.mind.org.uk

For peer review only - http://bmjopen.bmj.com/site/about/guidelines.xhtml

STROBE Statement—Checklist of items that should be included in reports of ***cohort studies***

	Item No	Recommendation	Page No.
**Title and abstract**	1	(*a*) Indicate the study's design with a commonly used term in the title or the abstract	1
		(*b*) Provide in the abstract an informative and balanced summary of what was done and what was found	2
**Introduction**			
Background/rationale	2	Explain the scientific background and rationale for the investigation being reported	4
Objectives	3	State specific objectives, including any prespecified hypotheses	4,5
**Methods**			
Study design	4	Present key elements of study design early in the paper	5
Setting	5	Describe the setting, locations, and relevant dates, including periods of recruitment, exposure, follow-up, and data collection	5-7
Participants	6	(*a*) Give the eligibility criteria, and the sources and methods of selection of participants. Describe methods of follow-up	5,6
		(*b*) For matched studies, give matching criteria and number of exposed and unexposed	NA
Variables	7	Clearly define all outcomes, exposures, predictors, potential confounders, and effect modifiers. Give diagnostic criteria, if applicable	8-10
Data sources/ measurement	8*	For each variable of interest, give sources of data and details of methods of assessment (measurement). Describe comparability of assessment methods if there is more than one group	8-10
Bias	9	Describe any efforts to address potential sources of bias	10
Study size	10	Explain how the study size was arrived at	6
Quantitative variables	11	Explain how quantitative variables were handled in the analyses. If applicable, describe which groupings were chosen and why	10
Statistical methods	12	(*a*) Describe all statistical methods, including those used to control for confounding	10
		(*b*) Describe any methods used to examine subgroups and interactions	10
		(*c*) Explain how missing data were addressed	NA
		(*d*) If applicable, explain how loss to follow-up was addressed	NA
		(*e*) Describe any sensitivity analyses	NA
**Results**			
Participants	13*	(a) Report numbers of individuals at each stage of study—eg numbers potentially eligible, examined for eligibility, confirmed eligible, included in the study, completing follow-up, and analysed	NA
		(b) Give reasons for non-participation at each stage	NA
		(c) Consider use of a flow diagram	NA
Descriptive data	14*	(a) Give characteristics of study participants (eg demographic, clinical, social) and information on exposures and potential confounders	NA
		(b) Indicate number of participants with missing data for each variable of interest	NA
		(c) Summarise follow-up time (eg, average and total amount)	NA
Outcome data	15*	Report numbers of outcome events or summary measures over time	NA
Main results	16	(*a*) Give unadjusted estimates and, if applicable, confounder-adjusted estimates and their precision (eg, 95% confidence interval). Make clear	NA

For peer review only - http://bmjopen.bmj.com/site/about/guidelines.xhtml

		which confounders were adjusted for and why they were included	
		(*b*) Report category boundaries when continuous variables were categorized	NA
		(*c*) If relevant, consider translating estimates of relative risk into absolute risk for a meaningful time period	NA
Other analyses	17	Report other analyses done—eg analyses of subgroups and interactions, and sensitivity analyses	NA
**Discussion**			
Key results	18	Summarise key results with reference to study objectives	NA
Limitations	19	Discuss limitations of the study, taking into account sources of potential bias or imprecision. Discuss both direction and magnitude of any potential bias	NA
Interpretation	20	Give a cautious overall interpretation of results considering objectives, limitations, multiplicity of analyses, results from similar studies, and other relevant evidence	NA
Generalisability	21	Discuss the generalisability (external validity) of the study results	NA
**Other information**			
Funding	22	Give the source of funding and the role of the funders for the present study and, if applicable, for the original study on which the present article is based	13

*Give information separately for exposed and unexposed groups.

Note: An Explanation and Elaboration article discusses each checklist item and gives methodological background and published examples of transparent reporting. The STROBE checklist is best used in conjunction with this article (freely available on the Web sites of PLoS Medicine at http://www.plosmedicine.org/, Annals of Internal Medicine at http://www.annals.org/, and Epidemiology at http://www.epidem.com/). Information on the STROBE Initiative is available at http://www.strobe-statement.org.

For peer review only
